# Construction of circRNA-miRNA-mRNA network and identification of novel potential biomarkers for non-small cell lung cancer

**DOI:** 10.1186/s12935-021-02278-z

**Published:** 2021-11-20

**Authors:** Jia Yang, Ran Hao, Yunlong Zhang, Haibin Deng, Wenjing Teng, Zhongqi Wang

**Affiliations:** 1 Department of Oncology, Longhua Hospital, Shanghai University of Traditional Chinese Medicine, 725 South of Wanping Road, Xuhui District, Shanghai, China; 2 School of Nursing, Hebei Medical University, Shijiazhuang, Hebei China; 3 Department of Oncology, Shanghai Municipal Hospital of Traditional Chinese Medicine, Shanghai University of Traditional Chinese Medicine, Shanghai, China

**Keywords:** circRNA, ceRNA network, Non-small cell lung cancer, Immune infiltration, Prognosis

## Abstract

**Background:**

The underlying circular RNAs (circRNAs)-related competitive endogenous RNA (ceRNA) mechanisms of pathogenesis and prognosis in non-small cell lung cancer (NSCLC) remain unclear.

**Methods:**

Differentially expressed circRNAs (DECs) in two Gene Expression Omnibus datasets (GSE101684 and GSE112214) were identified by utilizing R package (Limma). Circinteractome and StarBase databases were used to predict circRNA associated-miRNAs and mRNAs, respectively. Then, protein–protein interaction (PPI) network of hub genes and ceRNA network were constructed by STRING and Cytoscape. Also, analyses of functional enrichment, genomic mutation and diagnostic ROC were performed. TIMER database was used to analyze the association between immune infiltration and target genes. Kaplan–Meier analysis, cox regression and the nomogram prediction model were used to evaluate the prognostic value of target genes. Finally, the expression of potential circRNAs and target genes was validated in cell lines and tissues by quantitative real-time PCR (qRT-PCR) and Human Protein Atlas (HPA) database.

**Results:**

In this study, 15 DECs were identified between NSCLC tissues and adjacent-normal tissues in two GEO datasets. Following the qRT-PCR corroboration, 7 DECs (hsa_circ_0002017, hsa_circ_0069244, hsa_circ_026337, hsa_circ_0002346, hsa_circ_0007386, hsa_circ_0008234, hsa_circ_0006857) were dramatically downregulated in A549 and SK-MES-1 compared with HFL-1 cells. Then, 12 circRNA-sponged miRNAs were screened by Circinteractome and StarBase, especially, hsa-miR-767-3p and hsa-miR-767-5p were significantly up-regulated and relevant to the prognosis. Utilizing the miRDB and Cytoscape, 12 miRNA-target genes were found. Functional enrichment, genomic mutation and diagnostic analyses were also performed. Among them, FNBP1, AKT3, HERC1, COL4A1, TOLLIP, ARRB1, FZD4 and PIK3R1 were related to the immune infiltration via TIMER database. The expression of ARRB1, FNBP1, FZD4, and HERC1 was correlated with poor overall survival (OS) in NSCLC patients by cox regression and nomogram. Furthermore, the hub-mRNAs were validated in cell lines and tissues.

**Conclusion:**

We constructed the circRNA-miRNA-mRNA network that might provide novel insights into the pathogenesis of NSCLC and reveal promising immune infiltration and prognostic biomarkers.

**Supplementary Information:**

The online version contains supplementary material available at 10.1186/s12935-021-02278-z.

## Introduction

Lung cancer is one of the most frequent diagnosed cancer and has the highest rate of mortality [1]. There are almost 1.8 million new cases and over 1.6 million deaths of lung cancer every year. Non-small cell lung cancer (NSCLC), accounts for approximately 80–85% of lung cancer, including lung squamous cell carcinoma (LUSC), lung adenocarcinoma (LUAD), and large cell carcinoma [2]. Despite the advances treatment of targeted therapy and immunotherapy in NSCLC, the overall 5-year survival rate for patients remain poor [3]. Therefore, to reduce the enormous morbidity and mortality, there is an urgent need to explore and elucidate the novel targets and molecular mechanism of NSCLC.

Recently, accumulating evidences have indicated that circular RNA (circRNA), a new class of endogenous RNA with covalent loop and formed by reverse splicing of precursor mRNA [4], served as key role to regulate the target genes’ expression [5–7]. CircRNAs have been widely reported in pathogenesis of various cancers, suggesting circRNAs may be better predictive biomarkers and therapeutic targets [8, 9]. Particularly, circRNAs can bind the miRNA response element (MRE) and negatively regulate their activity, by acting as miRNA sponge and competing endogenous RNA (ceRNA), which play master roles in circRNA-miRNA-mRNA axis [10–12]. As far as NSCLC is concerned, hsa-circRNA-002178 was found to be upregulated and could act as a ceRNA via sponging miR-34 to induce PD1 expression in LUAD [13]. Zhou et al. identified that circ-ENO1 could promote the glycolysis, proliferation, migration and EMT of LUAD cells by acting as a ceRNA to interact with miR-22-3p and its host gene ENO1 [14]. Nevertheless, understanding of the pivotal circRNA-miRNA-mRNA ceRNA networks associated significantly with carcinogenesis and progression of NSCLC remain very limited, and novel therapeutic indicators and prognostic markers require further explored.

In the present study, we identified the differentially expressed circRNAs (DECs) between NSCLC tissues and paired adjacent normal tissues in two Gene Expression Omnibus (GEO) datasets (GSE101684 and GSE112214). Furthermore, a potential ceRNA network of circRNA-miRNA-mRNA regulatory axis in NSCLC was constructed and validated by a series of analyses in silico and experiments in vitro. Moreover, we also built a prognosis model and analyzed the association between immunocytes infiltration and ceRNA networks. This approach may shed light on the molecular mechanism of initiation and progression of NSCLC, and provide novel potential diagnostic or prognostic biomarkers.

## Materials and methods

### Data acquisition

Two NSCLC datasets (GSE101684 and GSE112214) were acquired from the NCBIs Gene Expression Omnibus (GEO; http://www.ncbi.nlm.nih.gov/geo/). There were eight samples in GSE101684 (4 tumor tissue samples vs. 4 adjacent normal tissue samples), and six samples in GSE112214 (3 tumor tissue samples vs. 3 normal tissue samples). GDC API (https://portal.gdc.cancer.gov/) [15] was used to download the gene expression data, clinical data and mutation data of NSCLC patients.

### Differential analysis for DECs

To obtain DECs related to the occurrence and development of NSCLC, R package limma (version 3.44.3) [16] was used to perform differential analysis. DECs were identified by |log2FC|> 1 and *P* < 0.05. The results were displayed by volcano plot and heatmap.

### CircBase analysis and cancer-specific circRNA database (CSCD) analysis

CircBase (http://cirbase.org/) [17] is a database which contains the sequencing data including large-scale circRNA to identify the novel circRNAs. In this study, we introduced the circBase database to obtain the mapping of candidate circRNAs and parental genes. CSCD (cancer specific circRNA database, http://gb.whu.edu.cn/CSCD/) [18] is a database of cancer-related circRNAs, which was used to obtain the structure loop diagram of candidate circRNAs.

### Circular RNA interactome (CRI) analysis

Circular RNA Interactome Database (CRI, https://circinteractome.irp.nia.nih.gov/) [19] is a network tool for investigating circRNAs and circRNA-interacting proteins and miRNAs. The predicted miRNAs that may combine with the candidate circRNAs were directly collected from the CRI database. The miRNAs predicted by CSCD database and CRI online were used as candidate DECs-binding miRNAs. Finally, the ceRNA network of circRNA-miRNA was visualized by Cytoscape (version 3.6.1) [20].

### StarBase analysis and Kaplan–Meier plotter analysis

StarBase database (http://starbase.sysu.edu.cn/) [21] is an open-access platform that can predict the ncRNA‐miRNA and miRNA‐mRNA regulatory networks and combine them to construct the ceRNA regulatory network. We utilized the StarBase V3.0 to analyze the expression level of miRNAs and target genes between lung cancer tissues (LUAD and LUSC) and normal tissues. In addition, through the Kaplan–Meier plotter database (http://kmplot.com/analysis/) [22], we evaluated the prognostic value of DECs-interacting miRNAs and miRNA-target genes in lung cancer (LUAD and LUSC) related to OS. The main data source was The Cancer Genome Atlas database (TCGA; https://tcga-data.nci.nih.gov/tcga/). *P* < 0.05 was considered statistically significant.

### Construction of PPI network and functional enrichment analysis

We used the miRDB database (http://mirdb.org/) [23] to predict the target genes of miRNAs, and used the STRING database version 11.0 (https://www.string-db.org/) [24] to construct the protein–protein interaction (PPI) network. The interacting gene pairs were downloaded from STRING database and visualized by Cytoscape (version 3.6.1) [20]. The hub gene of top 100 was obtained by MCC method of cytohubb [25] and were imported into Cytoscape [20] to construct the protein–protein interaction (PPI) network. In addition, we used R package clusterProfiler (version 3.16.1) [26] to analyze the statistical enrichment of miRNA-target genes in GO and KEGG.

### Immune infiltration analysis and ROC analysis

TIMER database (https://cistrome.shinyapps.io/timer/) [27] can be used to systematically analyze the infiltration of immune cells in tumor tissues, and provide the infiltration level of six types of immunocytes (CD4 + T cell, CD8 + T cell, B cell, macrophage, neutrophil, dendritic cell). The TIMER (Version 1) database was used to analyze the immune infiltration of miRNA target genes in lung cancer (LUAD and LUSC) samples from TCGA data.

We also drew receiver operating characteristic curves (ROC curves) of target genes in lung cancer (LUAD and LUSC) by using R-package pROC (version 1.17.0.1) [28] to explore the diagnostic potential of target genes. Besides, R package maftools (Version 2.4.12) [29] were used to evaluate the mutation frequency of miRNA target genes in lung cancer.

### Cox regression and prognostic nomogram

A univariate COX regression analysis was employed to identify the relationship between the target genes and patient’s OS by R package survival (Version 3.2.10) [30] and surviminer (Version 0.4.9) [31]. Then, multivariate COX analysis was employed to screen independent prognostic factors, and R package forestplot [32] was used to draw the forest map.

Thereafter, we constructed the nomogram to predict the survival rates of NSCLC patients by R package RMS (version 6.2.0) [33] and survival (version 3.2.10) [30]. The target genes were analyzed by DCA (version 1.1). Finally, the prognosis model could calculate the risk score predicting the 3-year, 5-year and 10-year survival rates for patients.

### Cells and culture conditions

Lung adenocarcinoma cells line (A549), lung squamous carcinoma cells line (SK-MES-1), and normal lung fibroblast cell line (HFL1) were purchased from the Institute of Biochemistry and Cell Biology, Chinese Academy of Sciences Shanghai. The A549 and SK-MES-1 were cultured in RPMI-1640 medium (Gibco, USA) supplemented with 10% fetal bovine serum (FBS) and antibiotics (100 units/mL penicillin and 100 μg/mL streptomycin). HFL1 was cultured in Ham's F-12 K (Kaigh’s) medium (Invitrogen, USA) containing 10% FBS and antibiotics. RPMI 1640 medium, fetal bovine serum (FBS), penicillin, and streptomycin were purchased from Gibco Life Technologies (Grand Island, NY, USA). All cells were incubated at 37 °C with 5% CO2 atmosphere.

### Validation by quantitative real-time PCR (qRT-PCR) and Immunohistochemistry (IHC)

We used cell lines to verify the selected DECs and target genes by qRT-PCR. RNA extraction and reverse transcription were performed on cells in logarithmic growth with Trizol reagent (Life Technologies, Carlsbad, CA), and purified RNA was reverse transcribed into cDNA using iScript cDNA synthesis kit (Bio-Rad Labs, Hercules, CA, USA). The experiment was repeated 3 times independently, and the primer sequences are shown in Table 1 and Table 2. The Human protein Atlas (HPA, version 20.1, https://www.proteinatlas.org/) [34] provides information on the tissue and cellular distribution of 24,000 human proteins. We examined the immunohistochemistry of target genes in normal lung tissues and NSCLC tissues (LUAD and LUSC).

**Table 1:** The primer sequences of circRNAs

circRNAs	Primer sequence
hsa_circ_0001320	F: GGAGGAAGGACAATTACCTG R: CTGTGAAGCAGTGTGCGAAG
hsa_circ_0006857	F: CTCCTGTGGTCAGCCTTACTG R: CTGTGGAGGGAGAGTCTTCAAC
hsa_circ_0007386	F: CCTTTCAGTTTTCGGCGTGG R: CAGGACAACGTGGAGAGAAC
hsa_circ_0008234	F: TGCTGCTGGAGGAGAACCTG R: CCCACATGCCTCTACCAATG
hsa_circ_0009043	F: CCCTCATGTTGTAGTTTTC R: CTGGAACATCTAGAGCATACC
hsa_circ_0027033	F: CATCTCAAACCTCCCACTGTC R: CTCTCCAACACCAAGGACAG
hsa_circ_0029426	F: CGTGAAACGTCATTTGACTGG R: GGTCGTTTTTCCAGTACC
hsa_circ_0043256	F: CTGTCCAGCCAGCCAGTATC R: CATGTGTAGAAGTAGATGTAC
hsa_circ_0069244	F: CTTACAGGCACAAAACGAGG R: CAGGTCCTGGATCAGCTGTC
hsa_circ_0002017	F: GGACTGATGACCAACTGCTTG R: GGATTGCTGCAGGTTCGAAT
hsa_circ_0002346	F: AACACCATTCGAACCTGCAG R: TTGGCAAAGTACAGCAACCA
hsa_circ_0004777	F: GGAGAAGAAAGAGCCTTGCC R: ACCTCTCCTTCCAGTCACTG
hsa_circ_0026337	F: TTGTTTGCGGGAAAGTACCAC R: GGAGAGGATGACTACCACGA
hsa_circ_0049271	F: GGTGGTGGTGTTGCTTATCT R: GCAACTCCACACAGCCAATC
hsa_circ_0072309	F: CCTGGATGGTGGACAATAAAAGA R: ACCCCTGTCGTTCCACTTTA

**Table 2:** The primer sequences of mRNAs

mRNAs	Primer sequence
ARRB1	F: TGATGACGACATTGTATTTGAGGAC R: AAGAAGACGAGTAAGCATCCGAGT
FNBP1	F: GGCTGGCAAGCACCATAAT R: CTTTCCACATAAAATTTCATCAGC
FZD4	F: ATTTCCACATTGCAGCCTGG R: TTGAACAAGGCCACCAAACC
HERC1	F: TAGCAAACCCTCATGACCGT R: CCAGAGACTCAAGACCAGCA

## Results

### Differential expression analysis for circRNAs

**Fig. 1:**
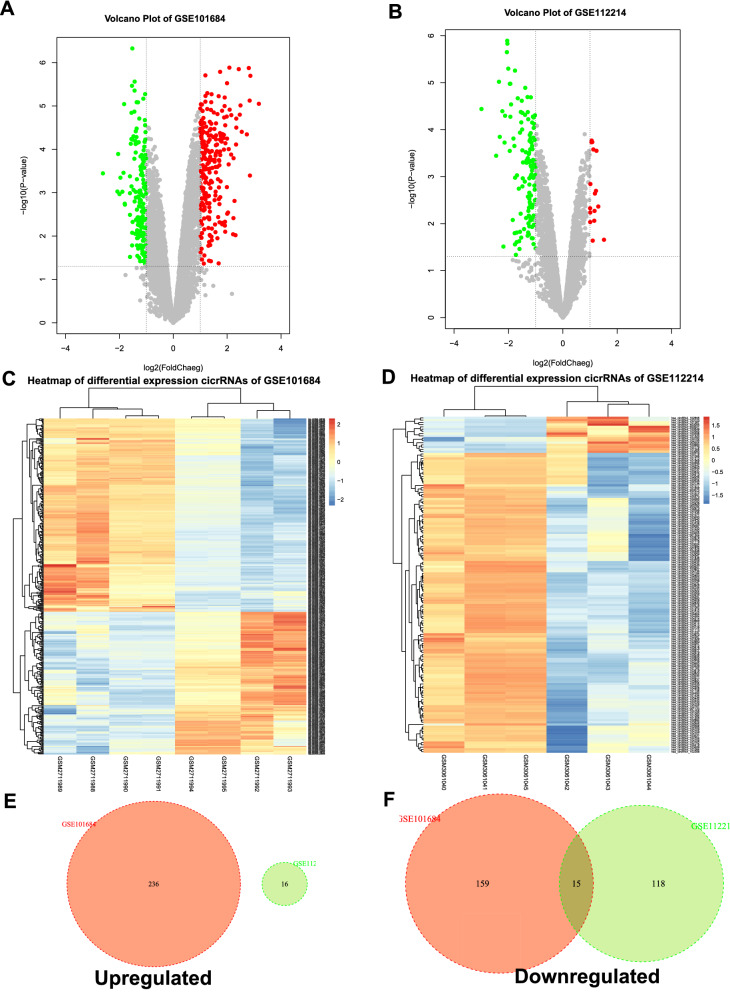
Differential expression of circRNAs in NSCLC. **A**, **B** Volcano map of DECs in GSE10684 and GSE112214. **C**, **D** Heat map of differential circRNA expression in GSE10684 and GSE112214. **E**, **F** Venn diagram of GSE10684 and GSE112214 upregulated and downregulated DECs. DECs, differentially expressed circRNAs; NSCLC, non-small cell lung cancer

**Fig. 2:**
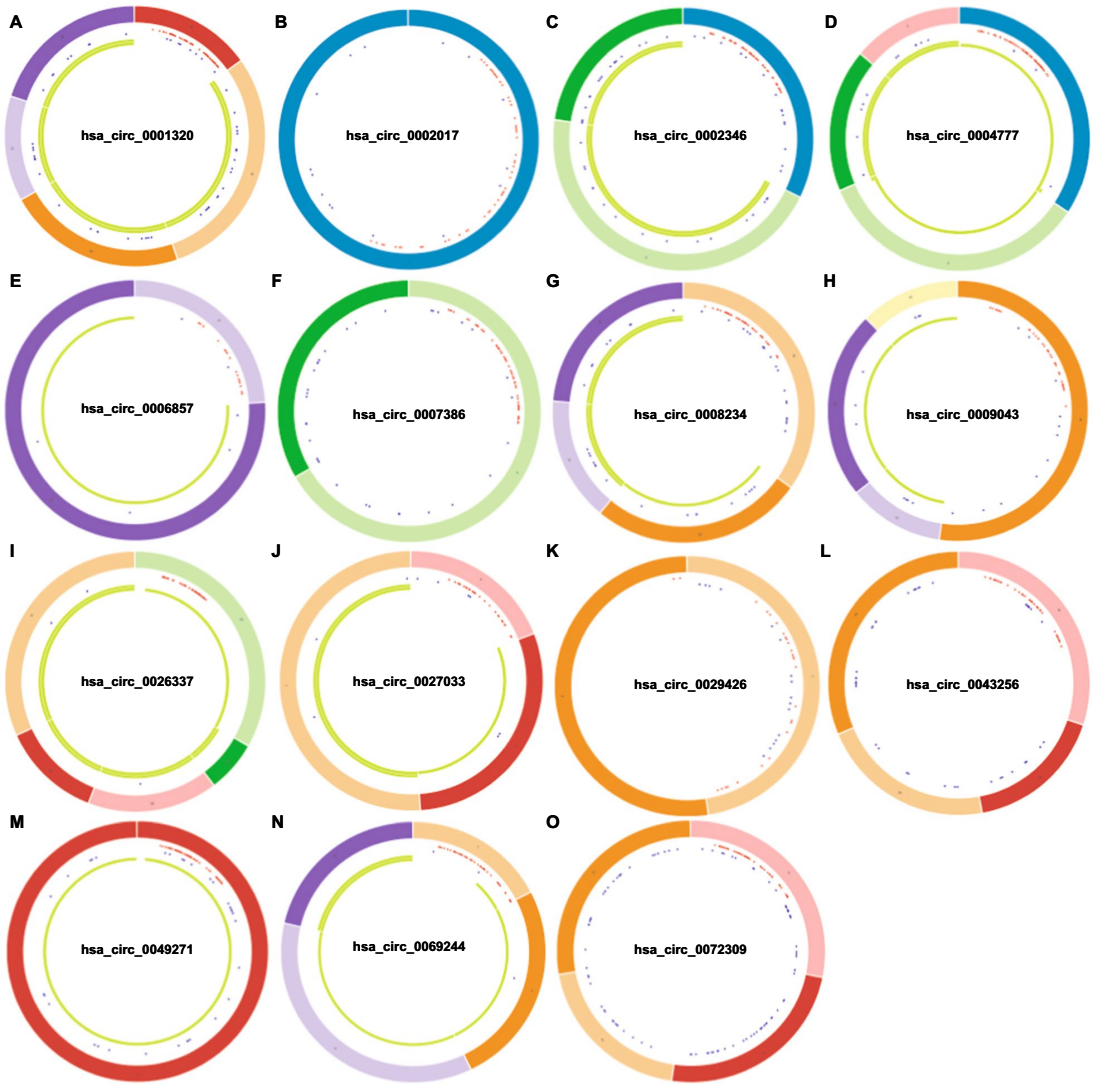
Structural patterns of fifteen DECs in NSCLC. **A**–**O** are respectively the structure of hsa_circ_0001320, hsa_circ_0002017, hsa_circ_0002346, hsa_circ_0004777, hsa_circ_0006857, hsa_circ_0007386, hsa_circ_0008234, hsa_circ_0009043, hsa_circ_0026337, hsa_circ_0027033, hsa_circ_0029426, hsa_circ_0043256, hsa_circ_0049271, hsa_circ_0069244, hsa_circ_0072309. DECs, differentially expressed circRNAs; NSCLC, non-small cell lung cancer

In order to identify the potential circRNAs related to progression and carcinogenesis of NSCLC, differential expression analysis was performed between NSCLC tissues and adjacent-normal lung tissues on two selected GEO datasets. There were 410 DECs in GSE101684, of which 236 were up-regulated, and 174 were down-regulated; there were 149 DECs in GSE112214, of which 16 were up-regulated and 133 were down-regulated, presenting by volcano plot and heatmap (Fig. 1A–D). The two datasets of up-regulated DECs had no intersection with each other (Fig. 1E), while there were fifteen down-regulated DECs from the intersection of the two datasets (Fig. 1F), which were hsa_circRNA_102761, hsa_circRNA_103415, hsa_circRNA_103414, hsa_circRNA_101083, hsa_circRNA_103820, hsa_circRNA_100432, hsa_circRNA_102442, hsa_circRNA_103606, hsa_circRNA_102683, hsa_circRNA_102046, hsa_circRNA_102677, hsa_circRNA_102678, hsa_circRNA_101066, hsa_circRNA_101213, hsa_circRNA_100850. The detailed information of these 15 candidate circRNAs from the circBase database, including circBase ID, genomic location, and parental gene, was listed in Table  3. The structure visualization of circRNAs was shown in Fig. 2.

**Table 3:** Fifteen candidate DECs associated with progression of NSCLC and their information

circRNA name	circBase ID	Parental gene	Location
hsa_circRNA_102761	hsa_circ_0009043	EXOC6B	chr2:72945231–72960247
hsa_circRNA_103415	hsa_circ_0008234	FOXP1	chr3:71090478–71102924
hsa_circRNA_103414	hsa_circ_0001320	FOXP1	chr3:71064699–71102924
hsa_circRNA_101083	hsa_circ_0027033	RBMS2	chr12:56962,758–56965639
hsa_circRNA_103820	hsa_circ_0072309	LIFR	chr5:38523520–38530768
hsa_circRNA_100432	hsa_circ_0004777	SOX13	chr1:204082042–204083733
hsa_circRNA_102442	hsa_circ_0049271	KEAP1	chr19:10610070–10610756
hsa_circRNA_103606	hsa_circ_0069244	LDB2	chr4:16587544–16760883
hsa_circRNA_102683	hsa_circ_0007386	CRIM1	chr2:36668400–36669878
hsa_circRNA_102046	hsa_circ_0043256	ACACA	chr17:35604934–35609962
hsa_circRNA_102677	hsa_circ_0002017	CRIM1	chr2:36623756–36623930
hsa_circRNA_102678	hsa_circ_0002346	CRIM1	chr2:36623756–36669878
hsa_circRNA_101066	hsa_circ_0026337	SCN8A	chr12:52180325–52188425
hsa_circRNA_101213	hsa_circ_0029426	RAN	chr12:131357380–131357465
hsa_circRNA_100850	hsa_circ_0006857	PACS1	chr11:66006274–66006748

*DECs* differentially expressed circRNAs, *NSCLC* non-small cell lung cancer

### Prediction and analysis of circRNAs-targeted miRNAs

**Fig. 3:**
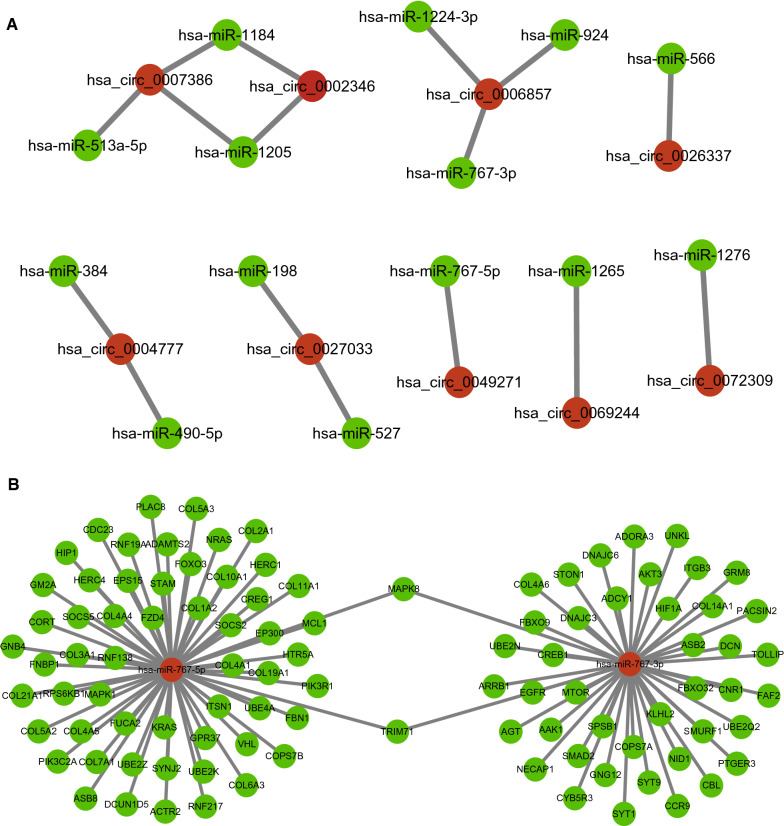
CeRNA networks of DECs-miRNAs and miRNA-target genes in NSCLC. **A** DECs-miRNA networks were constructed as shown; **B** presents the network of miRNA hsa-miR-767-3p and hsa-miR-767-5p and TOP 100 hub target genes. DECs, differentially expressed circRNAs; NSCLC, non-small cell lung cancer

Studies have shown that in the ceRNA network, circRNA can act as a miRNA sponge to regulate gene expression. Therefore, we used the CSCD database and the CRI database to predict potential binding miRNAs of DECs. A total of 14 miRNAs could be combined with the 15 down-regulated circRNAs. To better visualization, circRNA-miRNA networks were constructed as shown in Fig. 3A.

**Fig. 4:**
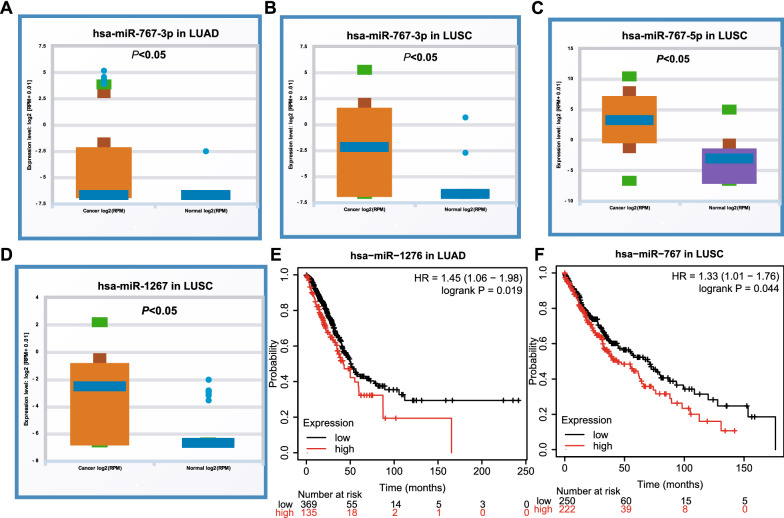
Expression and prognostic value of miRNAs in NSCLC. The expression of miRNAs was analyzed by using starBase, and hsa-miR-767-3p was were significantly upregulated in both LUAD (**A**) and LUSC tissues (**B**). hsa-miR-767-5p (**C**) and hsa-miR-1267 (**D**) were observed to be upregulated in LUSC tissues. Increased hsa-miR-1267 was relevant to the prognosis of LUAD patients (**E**), and increased hsa-miR-767 was relevant to the prognosis of LUSC patients (**F**). LUAD, lung adenocarcinoma; LUSC, lung squamous cell carcinoma

Furthermore, the expression of 14 miRNAs in NSCLC (LUAD and LUSC) was analyzed by using starBase (Fig. 4, Additional files 1, 2: Figs. S1 and S2). The results showed that compared with normal tissues, hsa-miR-767-3p, hsa-miR-767-5p and hsa-miR-278 were significantly up-regulated in NSCLC samples. In addition, the Kaplan–Meier plotter analysis was used to evaluate the prognostic value of these 14 miRNAs in NSCLC (Fig. 4, Additional files 3, 4: Figs. S3 and S4). Among the 14 miRNAs, only hsa-miR-1276 in LUAD and hsa-miR-767 in LUSC showed a negative correlation with the prognosis and expression. Therefore, only two miRNAs (hsa-miR-767-3p, hsa-miR-767-5p) were significantly up-regulated in lung cancer and were negatively associated with the survival time of NSCLC patients. They may the most potential candidates among all the circRNAs-targeted miRNAs in NSCLC.

### Prediction of miRNA target genes

The miRDB database was utilized to predict the putative target genes of hsa-miR-767-3p and hsa-miR-767-5p, and 1128 target genes were found. In order to explore the interaction between all target genes, we used STRING database to construct PPI network. A total of 4538 pairs of miRNA‐target were obtained, and the top 100 target genes in the network were defined as hub genes. Then, a miRNA-mRNA gene network composed of hsa-miR-767-3p, hsa-miR-767-5p and top 100 hub-genes was constructed by Cytoscape (Fig. 3B). In addition, the enrichment analysis for the target genes by Gene Ontology (GO) (Additional file 10: Table S1) and Kyoto Encyclopedia of Genes and Genomes (KEGG) pathway (Additional file 10: Table S2) were also performed, and indicated that they might participate in vital cancer-related biological processes, such as immune response, transforming growth factor beta signaling, cytokine‑cytokine receptor interaction, etc. (Additional file 5: Fig. S5).

**Fig. 5:**
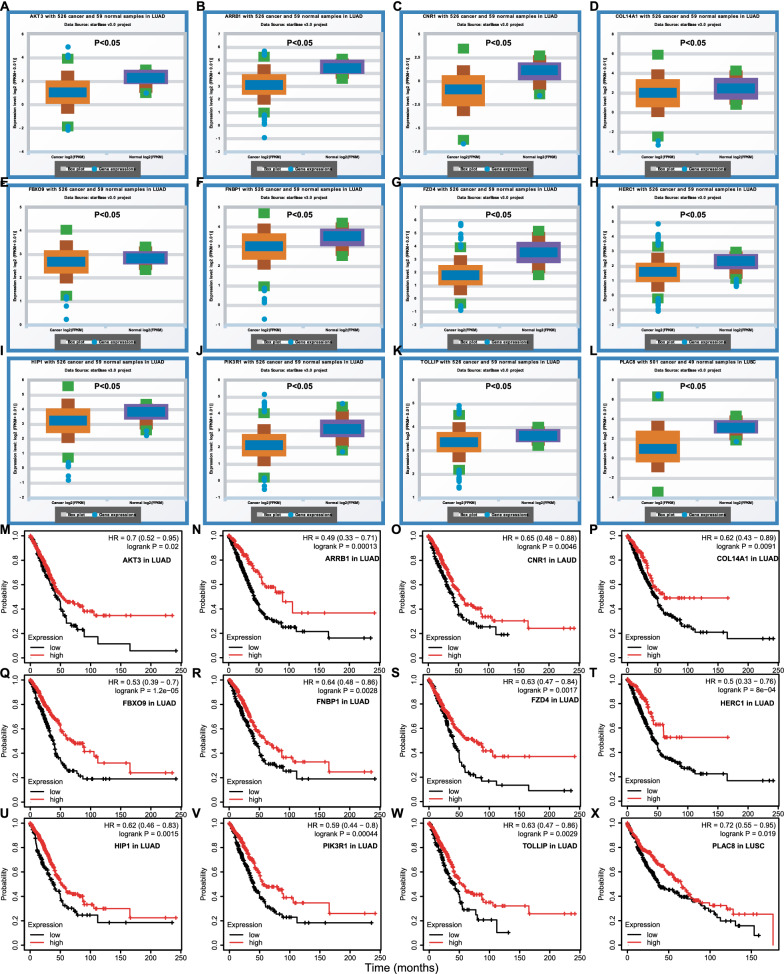
Expression and prognostic value of miRNA-target genes in NSCLC.** A**–**L** The eleven target genes (AKT3, ARRB1, CNR1, COL14A1, FBXO9, FNBP1, FZD4, HERC1, HIP1, PIK3R1, and TOLLIP) in LUAD and one target genes (PLAC8) in LUSC were significantly lower than that in normal samples, and their lower expression was significantly correlated with poor prognosis (**M**–**X**). NSCLC, non-small cell lung cancer; LUAD, lung adenocarcinoma; LUSC, lung squamous cell carcinoma

In order to improve analytic accuracy, we investigated the expression of the hub genes and their prognostic value in NSCLC (LAUD and LUSC). The eleven target genes (AKT3, ARRB1, CNR1, COL14A1, FBXO9, FNBP1, FZD4, HERC1, HIP1, PIK3R1, and TOLLIP) in LUAD and one target genes (PLAC8) in LUSC were significantly lower than that in normal samples (Fig. 5A–L), and their lower expression was significantly correlated with poor prognosis (Fig. 5M–X). Therefore, these 12 genes were most likely to be target genes regulated by the DECs through the circRNA-miRNA-mRNA ceRNA network in NSCLC.

### Immune infiltration analysis of target genes

**Fig. 6:**
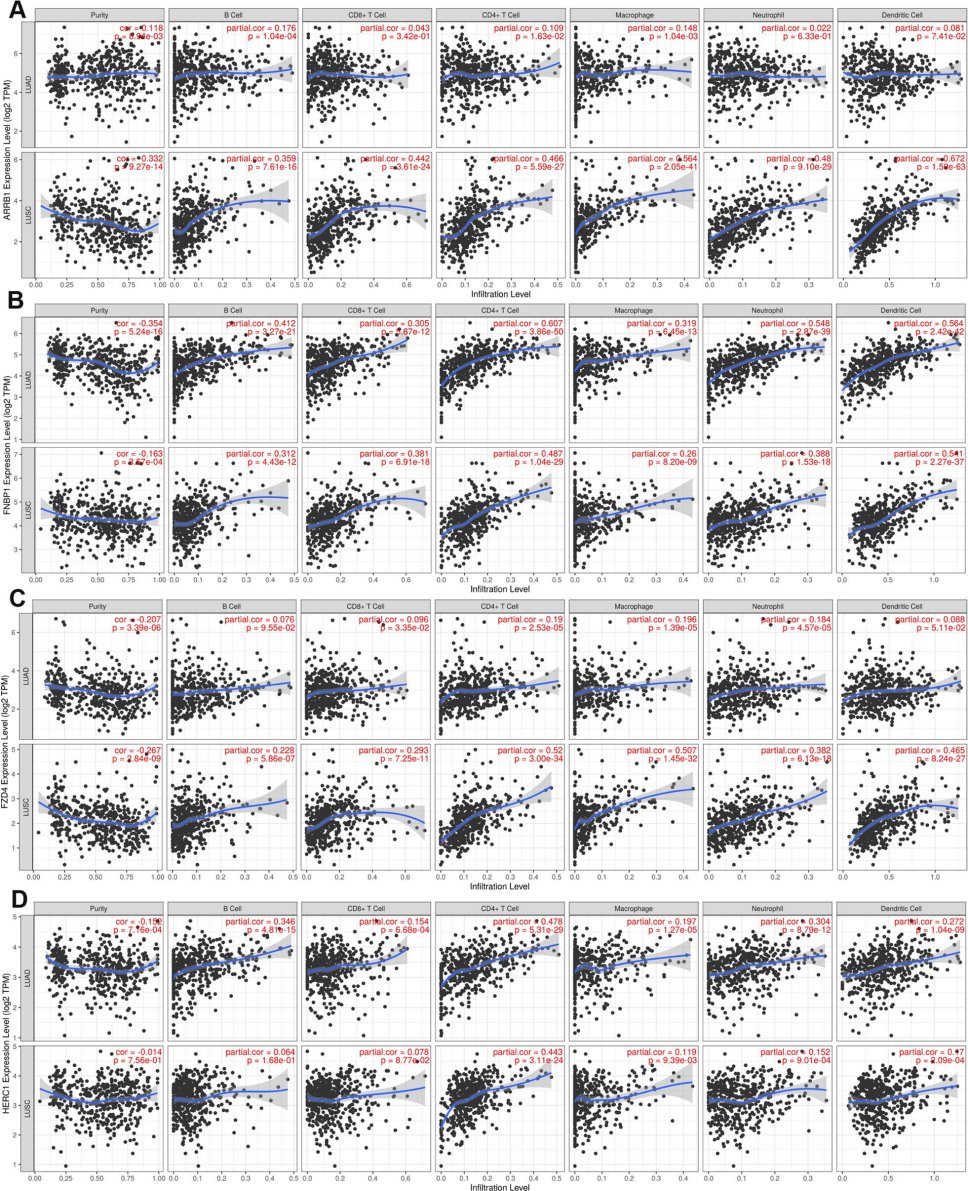
Correlation between miRNA-target genes and immune infiltration in NSCLC. The correlation between ARRB1 (**A**), FNBP1 (**B**), FZD4 (**C**), HERC1 (**D**) and immune infiltration in NSCLC was analyzed by using the TIMER database. NSCLC, non-small cell lung cancer

Since enrichment analysis suggested that the target genes may involve in immune response, we further systematically analyzed the correlation between these 12 miRNA target genes and immune infiltration in NSCLC (LUAD and LUSC) by using the TIMER database (Fig. 6, Additional files 6, 7: Figs. S6 and S7). For FNBP1 and AKT3, they were related to the infiltration of CD4 + T cells, neutrophils, macrophages, and dendritic cells. HERC1, COL4A1 and TOLLIP were only correlated to the infiltration of CD4 + T cells. ARRB1 and FZD4 were significantly associated with the infiltration of macrophages, neutrophils and dendritic cells in LUSC, while its expression was not associated with six immune cells in LUAD. Accordingly, PIK3R1 was only related to the infiltration of immune cells in LUAD. Nevertheless, the expression of other 4 genes correlation with the immune cells was modest. These results indicated that 8 miRNA target genes expression were associated with immune infiltration, which may affect the progression and prognosis of NSCLC.

### Receiver operating characteristic curve analysis of target genes

**Fig. 7:**
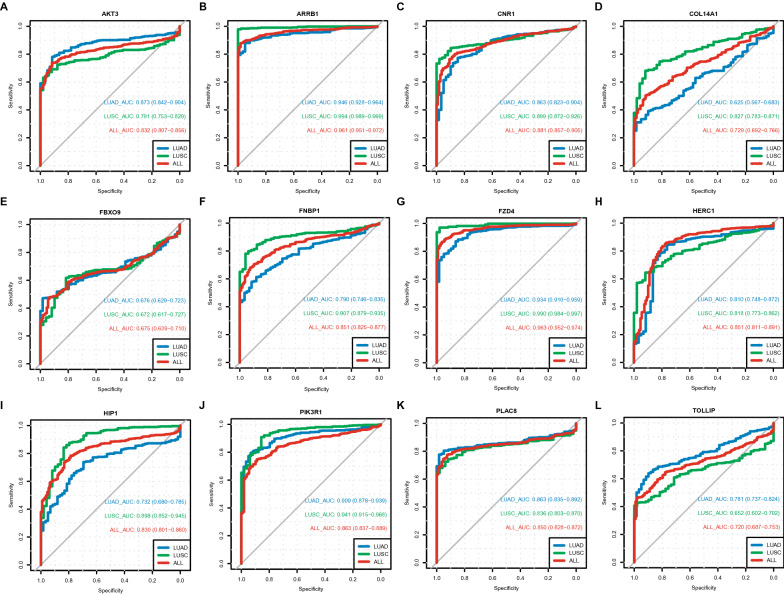
ROC curves of twelve miRNA-target genes in NSCLC. ROC analysis was performed to measure the discrimination value of twelve target genes in the diagnosis of NSCLC, and all AUC scores (**A**–**L**) were ranged from 0.675 to 0.963. NSCLC, non-small cell lung cancer

We performed ROC analysis to measure the discrimination value of 12 target genes in the diagnosis of NSCLC. As shown in Fig. 7, all AUC scores were ranged from 0.675 to 0.963, especially ARRB1(AUC = 0.961) and FZD4 (AUC = 0.963), indicating that these target genes may act as diagnostic biomarkers to distinguish LUAD or LUSC cases from normal cases.

Besides, the genomic mutation frequency analysis of 12 target genes in NSCLC patients was also performed, including the gene mutation classification, mutation type, SNV type, number of mutations per sample, and the ten most frequently mutated genes of the genome (Additional file 8: Fig. S8). The most common type of mutations were missense mutations, and no in-frame deletion mutations occurred. Among them, COL14A1 and HERC1 had the highest mutation frequency in LUAD and LUSC, respectively.

### Prognostic analysis of target genes

Univariate and multivariate Cox regression analyses were performed to evaluate the independent prognostic value of the 12 target genes in NSCLC patients with clinical features (Additional file 9: Fig. S9). The univariate COX regression revealed that 7 down-regulated genes (ARRB1, CNR1, COL4A1, FNBP1, FZD4, HERC1, and HIP1) were related to poor prognosis of NSCLC (Additional file 9: Fig. S9A). Other clinicopathological factors correlated with poor overall survival (OS) included male gender, advanced tumor stage, tumor metastasis or recurrence, history of smoking and without adjuvant treatment (Additional file 9: Fig. S9A). Subsequent multivariate Cox regression analysis indicated that only 4 target genes (ARRB1, FNBP1, FZD4, and HERC1) remained independently with OS. FZD4 and HERC1 were protective factors, while ARRB1 and FNBP1 were hazard factors (Additional file 9: Fig. S9B).

**Fig. 8:**
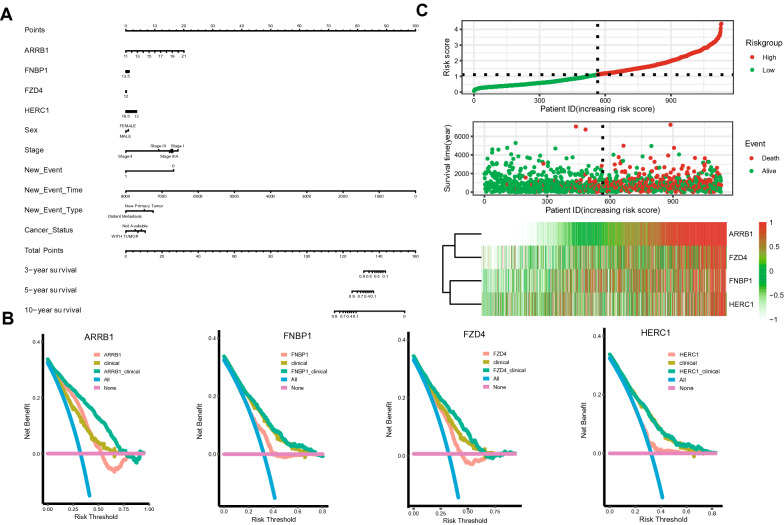
Correlation of the expression of ARRB1, FNBP1, FZD4, and HERC1 and clinical factors in NSCLC. **A** The nomogram integrated the expression of ARRB1, FNBP1, FZD4, and HERC1 and clinical factors were constructed to predict the probability of 3-, 5- and 10-year OS in NSCLC; **B** The DCA analysis also demonstrated that our nomogram has a high potential value for clinical; **C** Risk factor analysis was performed on these genes to evaluate the risk scores. NSCLC, non-small cell lung cancer; OS, overall survival

Based on the result of multivariate Cox regression, a nomogram integrated the expression of ARRB1, FNBP1, FZD4, and HERC1 and clinical factors (such as grade and stage) was constructed to predict the probability of 3-, 5- and 10-year OS in NSCLC (Fig. 8A). Furtherly, the DCA analysis also demonstrated that our nomogram has a high potential value for clinical. All the 4 genes showed prognostic impact on the OS in NSCLC, especially, ARRB1 and FZD4 (Fig. 8B). Finally, according to the prognosis model, we performed risk factor analysis on these genes. The results showed that the higher expression level of ARRB1 gene in the high-risk group, the shorter OS time in patients with NSCLC (Fig. 8C).

### Construction and validation the potential circRNA-miRNA-mRNA network in NSCLC

**Fig. 9:**
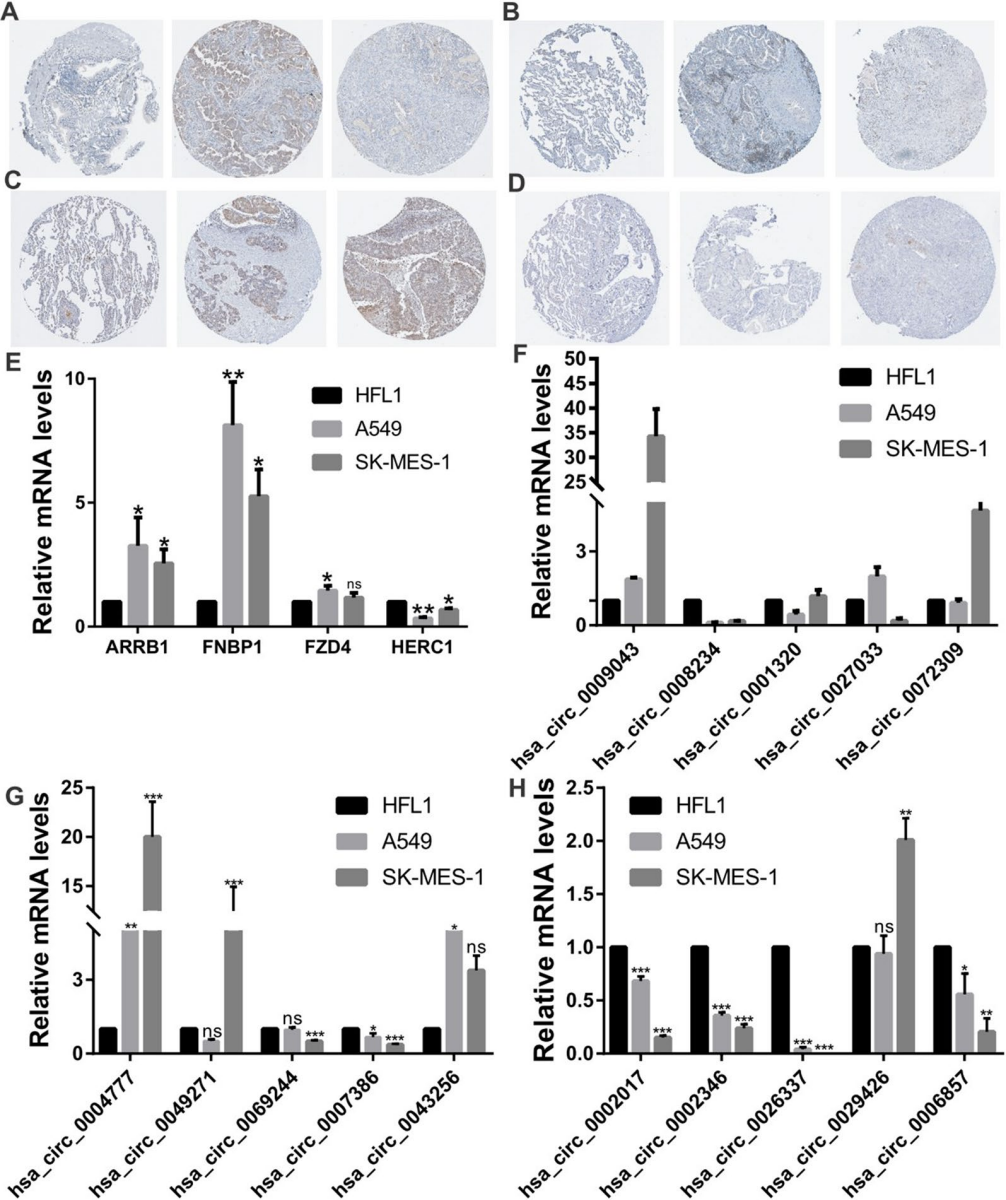
Validation of expression level of DECs and target genes in NSCLC cell lines. **A**–**E** The expression levels of four prognosis target genes (ARBB1, FNBP1, FZD4 and HERC1) were also detected by the HPA database tissues and cell lines. **F**–**H** The expression levels of fifteen DECs were detected by qRT-PCR in NSCLC cell lines. DECs, differentially expressed circRNAs; NSCLC, non-small cell lung cancer

In order to validate the expression of the candidate DECs and target genes, the qRT-PCR and IHC were performed in cell lines and HPA database tissues of NSCLC. The results showed that 7 of 15 circRNAs (hsa_circ_0002017, hsa_circ_0069244, hsa_circ_026337, hsa_circ_0002346, hsa_circ_0007386, hsa_circ_0008234, and hsa_circ_0006857), consisting with the predicted results, were dramatically downregulated in LUAD (A549) and LUSC (SK-MES-1) compared with normal lung cells (HFL1). Especially, the mRNA levels of hsa_circ_0002017, hsa_circ_0026337, hsa_circ_0002346 and hsa_circ_0008234 (*P* < 0.001) (Fig. 9F–H). Subsequently, the protein expression levels of 4 prognosis target genes (ARBB1, FNBP1, FZD4 and HERC1) were also detected by the HPA database tissues and cell lines. The results showed that the expression level of ARBB1 and FNBP1 were higher in LUAD, and FZD4 was upregulated in LUSC (Fig. 9A–D). Nevertheless, only HERC1 was significantly downregulated in both LUSC tissue and NSCLC cell lines, as predicted (Fig. 9E) (A549: *P* < 0.01; SK-MES-1: *P* < 0.05).

Above all, the circRNA-miRNA-mRNA subnetwork associated with progression and prognosis of NSCLC was constructed by 7 down-regulated circRNAs, 2 up-regulated miRNAs and 4 down-regulated mRNAs.

## Discussion

In this study, we first identified 15 differential expression circRNAs that were down-regulated in NSCLC from the intersection of two datasets. Subsequently, we explored the potential miRNA interacting with 15 circRNAs and constructed the circRNA-miRNA regulatory network. By analyzing the expression and prognostic value of the 14 miRNAs, 2 key miRNA showed the opposite expression trends to circRNAs and were related to the prognosis of NSCLC. Moreover, we predicted 12 miRNA-targeted genes, that were decreased in NSCLC and their downregulation related with poorer prognosis of NSCLC patients. Furthermore, the analysis of related pathway, immunocytes infiltration, mutations and clinical significance were also performed for the target genes. Additionally, we validated the expression level of 15 circRNAs and 4 miRNA-targeted genes in NSCLC cell lines and database tissues.

In our study, a total of 15 circRNAs were identified involved in the ceRNA network. Following the qRT-PCR showed that 7 circRNAs were dramatically down-regulated in LUSC and LUAD, which included hsa_circ_0002017, hsa_circ_0069244, hsa_circ_026337, hsa_circ_0002346, hsa_circ_0007386, hsa_circ_0008234, and hsa_circ_0006857 (Fig. 9F-10H). Among them, hsa_circ_0069244 (circLDB2) can served as a ceRNA to induce LIMCH1 expression to suppress the development of tumor and promote cisplatin sensitivity in LUAD by binding to miR-346 [35]. Hsa_circ_0002346 (circCRIM1), can acted as an independent risk factor and associated with the invasion and metastasis in LUAD by miR‐182/miR‐93 leukemia inhibitory factor receptor pathway [36]. Jiang et al. showed that hsa_circ_0008234 (circFOXP1) could sponge miR-574-5P to regulate RND3, which inhibits the progression of LUAD [37]. Nevertheless, hsa_circ_0008234 was found significantly up-regulated in cutaneous squamous cell carcinoma and gallbladder cancer to promote tumor progression and Warburg effect [38, 39]. However, to the best of our knowledge, none of the other 4 circRNAs (hsa_circ_0002017, hsa_circ_026337, hsa_circ_0007386, hsa_circ_0006857) have been reported, which need further in vitro and in vivo experiments and can serve as novel potential biomarkers for NSCLC patients.

Thus, the potential circRNA-sponged miRNAs were predicted via the intersection of two databases CSCD and CRI, 14 miRNAs were picked out. Among them, hsa-miR-767-3p and hsa-miR-767-5p were observed to be upregulated in LUSC tissues, and relevant to the prognosis of LUSC patients (Fig. 4E and F). Additionally, the level of hsa-miR-767-3p was higher in LUAD tissues than that in normal tissues (Fig. 4A). Thus, we identified hsa-miR-767-3p and hsa-miR-767-5p to be the key miRNAs related with circRNAs in NSCLC. Feng et al. revealed that miR-767-5p might be a tumor drive through targeting MAPK4 to protect against multiple myeloma [40], which was consistent with our findings. However, it was reported that miR-767-3p was downregulation in LUAD tissue and cell lines, suppressed the expression of CLDN18, and inhibited the LUAD cell proliferation [41]. Xu et al. found that hsa_circ_0018818 knockdown inhibited NSCLC tumorigenesis by functioning the miR-767-3p/Nidogen 1 signaling axis [42]. We speculated the mechanisms of miRNAs may vary among different cancers.

To further elucidate the downstream mechanism of the circRNA-miRNA-mRNA network, we predicted the target genes of hsa-miR-767-3p and hsa-miR-767-5p by miRDB database, and a total of 1128 genes were obtained. Based on the ceRNA hypothesis, the expression of target genes should have the opposite trend with miRNAs. At the end, 12 downregulated hub genes were identified from the PPI network according to node degree by utilizing the cytoscape. All the above 12 genes predicted the poor prognosis of NSCLC patients (Fig. 5M–X). Furthermore, function enrichment analysis indicated that these target mRNAs were mainly involved in the process of tumorigenesis and immune response. By using the TIMER database, immune infiltration analysis showed that 8 of 12 miRNA-target genes expression were significantly associated with the infiltration degree of CD4 + T cells, CD8 + T cells, DCs, B cells, neutrophils, and macrophages, which may affect the pathogenesis of NSCLC (Fig. 6).

Subsequently, a nomogram model combining target genes with other clinicopathological parameters was performed. The actual values for 1-, 3-, 5-year OS have a consistent with predicted values based on the calibration plot. Particularly, utilizing the DCA and factor analysis, we identified that ARRB1 showed the strongest impact on the prognosis outcome of NSCLC patients (Fig. 8B). Previous studies demonstrated that ARRB1 acted as a scaffold protein which can promote the progression of NSCLC and served as a promising biomarker in LUAD [43–45]. Another hub gene from the circRNA-miRNA-mRNA network was FZD4. FZD4 is coupled to the β-catenin signaling pathway, which leads to the activation of Wnt target genes, inhibition of GSK-3 kinase [46]. Prior researches have indicated that FZD4 may regulate the tumor microenvironment and the infiltration of immunocytes through the activation of Wnt/β-catenin pathway. When we further validated the 4 prognostic hub genes in cell lines and database tissues of NSCLC, only HERC1 shows a significantly decreased in LUSC and LUAD, in accordance with TCGA (Fig. 9E). It may relate to the inter-heterogeneity in different studies and samples. Similar to this research, Rossi et al. have also revealed that HERC1 can regulate cell migration and invasion, and has an inverse correlation with breast cancer patients OS [47]. In short, the mRNAs in circRNA‐related ceRNA networks may have key roles in the progression of NSCLC patients.

However, several limitations should be considered. First, the current findings need to be confirmed in a prospective study with larger sample sizes. Second, it was not convincing enough to portrait it as potential biomarkers with limited experimental evidence. Thus, further in vivo validation using an animal model and functional experiments need to be performed to reveal its underlying mechanism in NSCLC.

## Conclusion

In conclusion, we constructed a potential ceRNA network of circRNA-miRNA-mRNA in NSCLC based on GEO datasets mining, a series of bioinformatic analyses and validation experiments in vitro. In addition, the expression of hub genes was related with the prognosis outcome and immunocytes infiltration of NSCLC patients. These findings identified potential diagnostic or prognostic biomarkers and provide novel insights into the underlying mechanisms of carcinogenesis and progression of NSCLC.

## Supplementary Information

**Additional file 1:Fig. S1** Expression level of miRNAs in LUAD (**a-m**). The expression of miRNAs was analyzed by using starBase in LUAD. LUAD, lung adenocarcinoma.

**Additional file 2:Fig. S2** Expression level of miRNAs in LUSC (**a-k**). The expression of miRNAs was analyzed by using starBase in LUSC. LUSC, lung squamous cell carcinoma.

**Additional file 3:Fig. S3** The overall survival (OS) curves of miRNAs in LUAD. LUAD, lung adenocarcinoma.

**Additional file 4:** **Fig. S4** The overall survival (OS) curves of miRNAs in LUSC. LUSC, lung squamous cell carcinoma.

**Additional file 5:** **Fig. S5** Funrichment analysis of top 100 hub-genes in NSCLC. GO (**a**) and KEGG (**b**) pathway were performed to explore the potential biological mechanisms, and EGFR signaling pathway was associated with hub-genes (**c**). NSCLC, non-small cell lung cancer; GO, gene ontology; KEGG, kyoto encyclopedia of genes and genomes.

**Additional file 6:** **Fig. S6** Correlation between miRNA-target genes and immune infiltration in NSCLC. The correlation between AKT3 (**a**), CNR1 (**b**), COL14A1 (**c**), FBXO9 (**d**) and immune infiltration in NSCLC was analyzed by using the TIMER database. NSCLC, non-small cell lung cancer.

**Additional file 7:** **Fig. S7** Correlation between miRNA-target genes and immune infiltration in NSCLC. The correlation between HIP1 (**a**), PIK3R1 (**b**), PLAC8 (**c**), TOLLIP (**d**) and immune infiltration in NSCLC was analyzed by using the TIMER database. NSCLC, non-small cell lung cancer.

**Additional file 8:** **Fig. S8** Frequencies of mutations in miRNA-target gene. **l**. The overall situation of mutations in LUAD patients in TCGA. **b**. The overall situation of mutations in LUSC patients in TCGA. **C**. The mutation frequency of 12 target genes in NSCLC. NSCLC, non-small cell lung cancer; LUAD, lung adenocarcinoma; LUSC, lung squamous cell carcinoma.

**Additional file 9:** **Fig. S9** Cox regression analyses of miRNA-target gene. The univariate COX regression (**a**) and multivariate COX regression (**b**) were used to evaluate the independent prognostic value of the twelve target genes in NSCLC patients with clinical features. NSCLC, non-small cell lung cancer.

**Additional file 10:** **Table S1.** Results of GO enrichment. **Table S2.** Results of KEGG enrichment.

## Data Availability

The datasets generated and/or analyzed during the current study are available in GEO (http:// www.ncbi.nlm.nih.gov/geo) and GDC API (https://portal.gdc.cancer.gov/) website.

## Abbreviations

ceRNA: Competitive endogenous RNA
circRNAs: Circular RNAs
DECs: Differentially expressed circRNAs
HPA: Human Protein Atlas
GEO: Gene Expression Omnibus
LUAD: Lung adenocarcinoma
LUSC: Lung squamous cell carcinoma
MRE: MiRNA response element
NSCLC: Non-small cell lung cancer
qRT-PCR: Quantitative real-time PCR

## References

[1] Jemal A, Siegel R, Xu J, Ward E (2010). Cancer statistics, 2010. CA Cancer J Clin.

[2] Herbst RS, Morgensztern D, Boshoff C (2018). The biology and management of non-small cell lung cancer. Nature.

[3] Zhan J, Wang P, Li S, Song J, He H, Wang Y, Liu Z, Wang F, Bai H, Fang W (2019). HOXB13 networking with ABCG1/EZH2/Slug mediates metastasis and confers resistance to cisplatin in lung adenocarcinoma patients. Theranostics.

[4] Memczak S, Jens M, Elefsinioti A, Torti F, Krueger J, Rybak A, Maier L, Mackowiak SD, Gregersen LH, Munschauer M (2013). Circular RNAs are a large class of animal RNAs with regulatory potency. Nature.

[5] Chan JJ, Tay Y (2018). Noncoding RNA: RNA regulatory networks in cancer. Int J Mol Sci.

[6] Tay Y, Rinn J, Pandolfi PP (2014). The multilayered complexity of ceRNA crosstalk and competition. Nature.

[7] Hansen TB, Jensen TI, Clausen BH, Bramsen JB, Finsen B, Damgaard CK, Kjems J (2013). Natural RNA circles function as efficient microRNA sponges. Nature.

[8] Tang X, Ren H, Guo M, Qian J, Yang Y, Gu C (2021). Review on circular RNAs and new insights into their roles in cancer. Comput Struct Biotechnol J.

[9] Chen B, Huang S (2018). Circular RNA: an emerging non-coding RNA as a regulator and biomarker in cancer. Cancer Lett.

[10] Kristensen LS, Andersen MS, Stagsted LVW, Ebbesen KK, Hansen TB, Kjems J (2019). The biogenesis, biology and characterization of circular RNAs. Nat Rev Genet.

[11] Chen LL (2020). The expanding regulatory mechanisms and cellular functions of circular RNAs. Nat Rev Mol Cell Biol.

[12] Cheng Z, Yu C, Cui S, Wang H, Jin H, Wang C, Li B, Qin M, Yang C, He J (2019). circTP63 functions as a ceRNA to promote lung squamous cell carcinoma progression by upregulating FOXM1. Nat Commun.

[13] Wang J, Zhao X, Wang Y, Ren F, Sun D, Yan Y, Kong X, Bu J, Liu M, Xu S (2020). circRNA-002178 act as a ceRNA to promote PDL1/PD1 expression in lung adenocarcinoma. Cell Death Dis.

[14] Zhou J, Zhang S, Chen Z, He Z, Xu Y, Li Z (2019). CircRNA-ENO1 promoted glycolysis and tumor progression in lung adenocarcinoma through upregulating its host gene ENO1. Cell Death Dis.

[15] Tomczak K, Czerwińska P, Wiznerowicz M (2015). The Cancer Genome Atlas (TCGA): an immeasurable source of knowledge. Contemp Oncol.

[16] Ritchie ME, Phipson B, Wu D, Hu Y, Law CW, Shi W, Smyth GK (2015). Limma powers differential expression analyses for RNA-sequencing and microarray studies. Nucleic Acids Res.

[17] Glažar P, Papavasileiou P, Rajewsky N (2014). circBase: a database for circular RNAs. RNA.

[18] Xia S, Feng J, Chen K, Ma Y, Gong J, Cai F, Jin Y, Gao Y, Xia L, Chang H (2018). CSCD: a database for cancer-specific circular RNAs. Nucleic Acids Res.

[19] Dudekula DB, Panda AC, Grammatikakis I, De S, Abdelmohsen K, Gorospe M (2016). CircInteractome: a web tool for exploring circular RNAs and their interacting proteins and microRNAs. RNA Biol.

[20] Shannon P, Markiel A, Ozier O, Baliga NS, Wang JT, Ramage D, Amin N, Schwikowski B, Ideker T (2003). Cytoscape: a software environment for integrated models of biomolecular interaction networks. Genome Res.

[21] Li JH, Liu S, Zhou H, Qu LH, Yang JH (2014). starBase v20: decoding miRNA-ceRNA, miRNA-ncRNA and protein-RNA interaction networks from large-scale CLIP-Seq data. Nucleic Acids Res..

[22] Nagy Á, Munkácsy G, Győrffy B (2021). Pancancer survival analysis of cancer hallmark genes. Sci Rep.

[23] Chen Y, Wang X (2020). miRDB: an online database for prediction of functional microRNA targets. Nucleic Acids Res.

[24] Szklarczyk D, Gable AL, Lyon D, Junge A, Wyder S, Huerta-Cepas J, Simonovic M, Doncheva NT, Morris JH, Bork P (2019). STRING v11: protein–protein association networks with increased coverage, supporting functional discovery in genome-wide experimental datasets. Nucleic Acids Res.

[25] Chin CH, Chen SH, Wu HH, Ho CW, Ko MT, Lin CY (2014). cytoHubba: identifying hub objects and sub-networks from complex interactome. BMC Syst Biol.

[26] Yu G, Wang LG, Han Y, He QY (2012). clusterProfiler: an R package for comparing biological themes among gene clusters. OMICS.

[27] Li T, Fan J, Wang B, Traugh N, Chen Q, Liu JS, Li B, Liu XS (2017). TIMER: a web server for comprehensive analysis of tumor-infiltrating immune cells. Cancer Res.

[28] Robin X, Turck N, Hainard A, Tiberti N, Lisacek F, Sanchez JC, Müller M (2011). pROC: an open-source package for R and S+ to analyze and compare ROC curves. BMC Bioinformatics.

[29] Mayakonda A, Lin DC, Assenov Y, Plass C, Koeffler HP (2018). Maftools: efficient and comprehensive analysis of somatic variants in cancer. Genome Res.

[30] Laajala TD, Murtojärvi M, Virkki A, Aittokallio T (2018). ePCR: an R-package for survival and time-to-event prediction in advanced prostate cancer, applied to real-world patient cohorts. Bioinformatics.

[31] Li G, Gao Y, Li K, Lin A, Jiang Z (2020). Genomic analysis of biomarkers related to the prognosis of acute myeloid leukemia. Oncol Lett.

[32] Pimple U (2020). Dataset on plot inventories of species diversity and structural parameters of natural and rehabilitated mangrove forest in the Trat province of Thailand. Data Brief.

[33] Ito K, Murphy D (2013). Application of ggplot2 to Pharmacometric Graphics. CPT Pharmacometrics Syst Pharmacol.

[34] Uhlén M, Fagerberg L, Hallström BM, Lindskog C, Oksvold P, Mardinoglu A, Sivertsson Å, Kampf C, Sjöstedt E, Asplund A (2015). Tissue-based map of the human proteome. Science.

[35] Wang Y, Li L, Zhang W, Zhang G (2021). Circular RNA circLDB2 functions as a competing endogenous RNA to suppress development and promote cisplatin sensitivity in non-squamous non-small cell lung cancer. Thorac Cancer.

[36] Wang L, Liang Y, Mao Q, Xia W, Chen B, Shen H, Xu L, Jiang F, Dong G (2019). Circular RNA circCRIM1 inhibits invasion and metastasis in lung adenocarcinoma through the microRNA (miR)-182/miR-93-leukemia inhibitory factor receptor pathway. Cancer Sci.

[37] Jiang W, He Y, Ma Z, Zhang Y, Zhang C, Zheng N, Tang X (2021). hsa_circ_0008234 inhibits the progression of lung adenocarcinoma by sponging miR-574-5p. Cell Death Discov.

[38] Cai L, Wang Y, Wu J, Wu G (2021). Hsa_circ_0008234 facilitates proliferation of cutaneous squamous cell carcinoma through targeting miR-127-5p to regulate ADCY7. Arch Dermatol Res.

[39] Wang S, Zhang Y, Cai Q, Ma M, Jin LY, Weng M, Zhou D, Tang Z, Wang JD, Quan Z (2019). Circular RNA FOXP1 promotes tumor progression and Warburg effect in gallbladder cancer by regulating PKLR expression. Mol Cancer.

[40] Feng Y, Zhang L, Wu J, Khadka B, Fang Z, Gu J, Tang B, Xiao R, Pan G, Liu J (2019). CircRNA circ_0000190 inhibits the progression of multiple myeloma through modulating miR-767-5p/MAPK4 pathway. J Exp Clin Cancer Res.

[41] Wan YL, Dai HJ, Liu W, Ma HT (2018). miR-767-3p inhibits growth and migration of lung adenocarcinoma cells by regulating CLDN18. Oncol Res.

[42] Xu X, Zhou X, Gao C, Cui Y (2020). Hsa_circ_0018818 knockdown suppresses tumorigenesis in non-small cell lung cancer by sponging miR-767-3p. Aging.

[43] Shen H, Wang L, Zhang J, Dong W, Zhang T, Ni Y, Cao H, Wang K, Li Y, Wang Y (2017). ARRB1 enhances the chemosensitivity of lung cancer through the mediation of DNA damage response. Oncol Rep.

[44] Ma Z, Yu YR, Badea CT, Kovacs JJ, Xiong X, Comhair S, Piantadosi CA, Rajagopal S (2019). Vascular endothelial growth factor receptor 3 regulates endothelial function through β-arrestin 1. Circulation.

[45] Qiu C, Zheng C, Zhu L, Qu X, Shen H, Du J (2015). β-arrestin1 over-expression is associated with an unfavorable prognosis in lung adenocarcinomas and correlated with vascular endothelial growth factor. Int J Clin Exp Pathol.

[46] Yang S, Wu Y, Xu TH, de Waal PW, He Y, Pu M, Chen Y, DeBruine ZJ, Zhang B, Zaidi SA (2018). Crystal structure of the Frizzled 4 receptor in a ligand-free state. Nature.

[47] Rossi FA, CalvoRoitberg EH, Enriqué Steinberg JH, Joshi MU, Espinosa JM, Rossi M (2021). HERC1 regulates breast cancer cells migration and invasion. Cancers.

