# Supplementary figures and images for: Construction of circRNA-miRNA-mRNA network and identification of novel potential biomarkers for non-small cell lung cancer
Source: Cancer Cell Int. 2021 Nov 20;21:611. doi: 10.1186/s12935-021-02278-z (PMC8605517; doi:10.1186/s12935-021-02278-z)

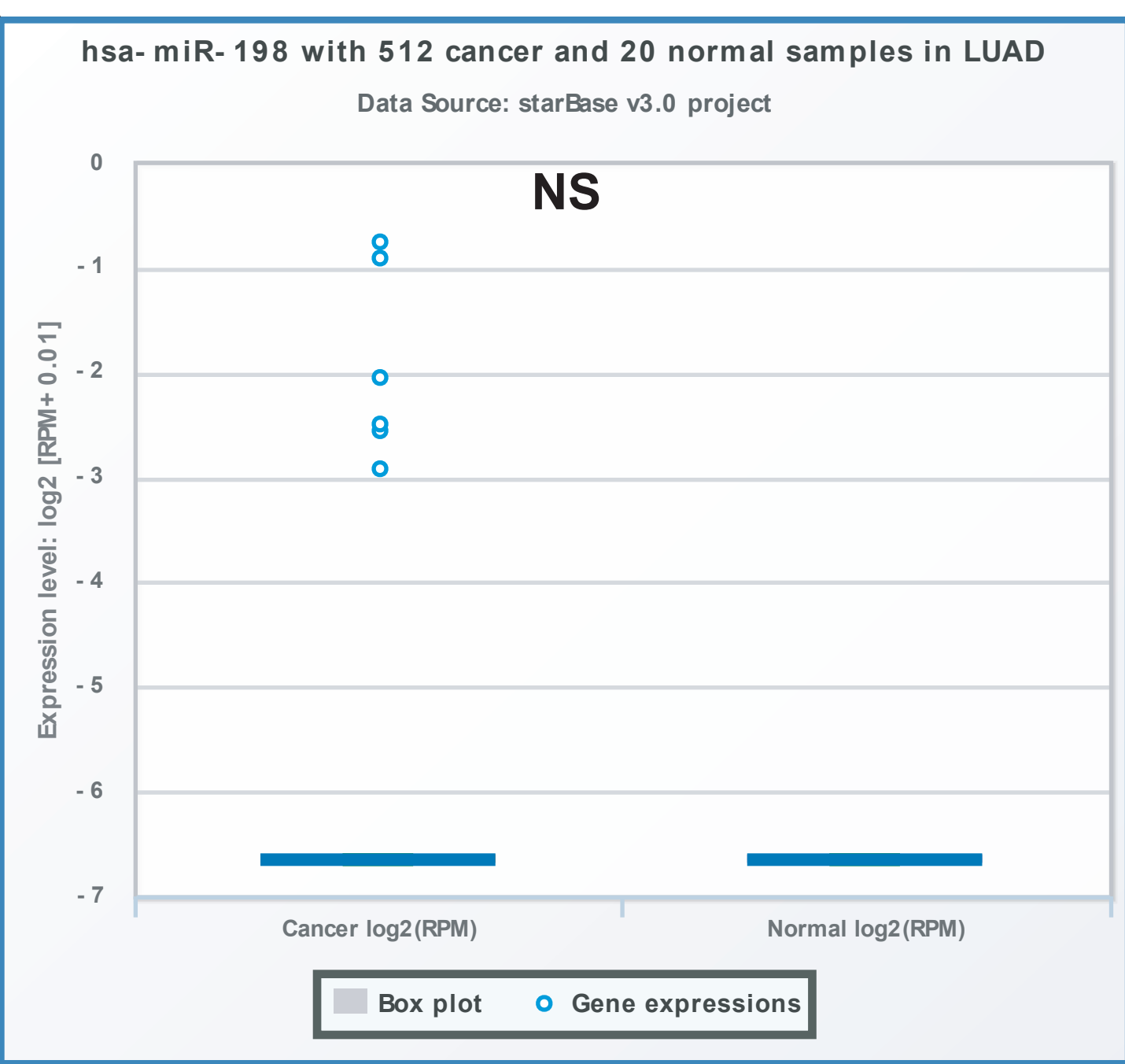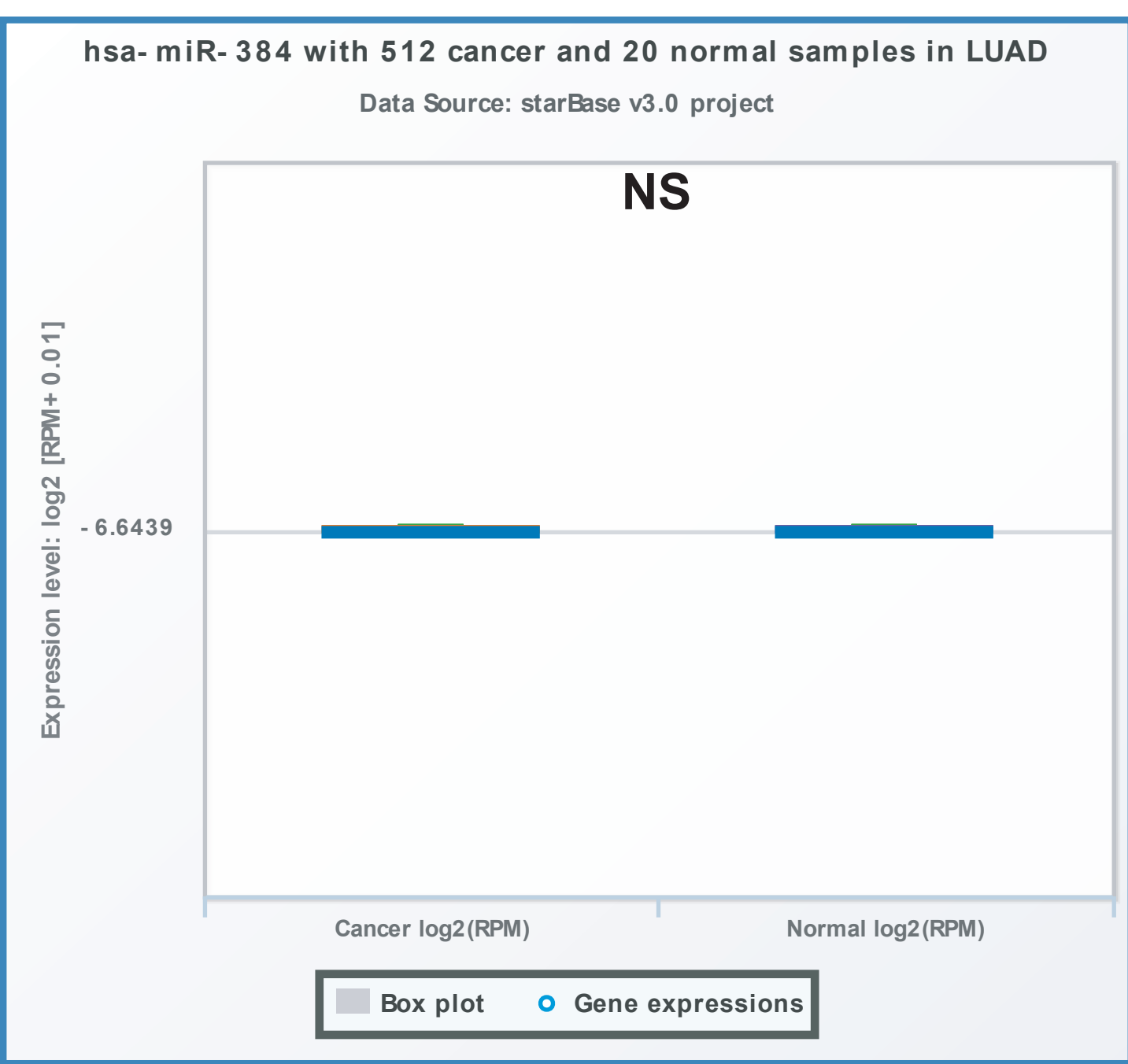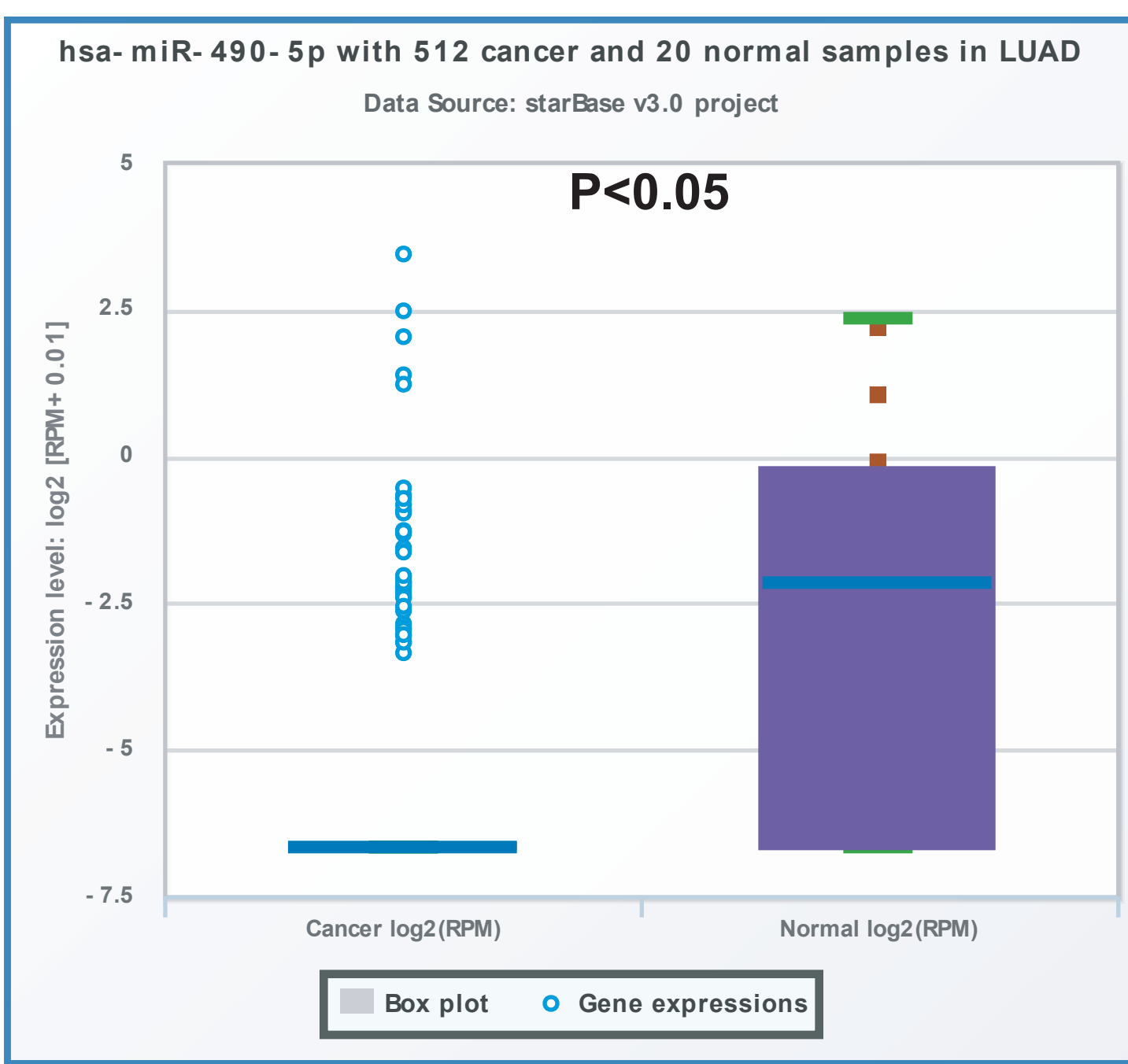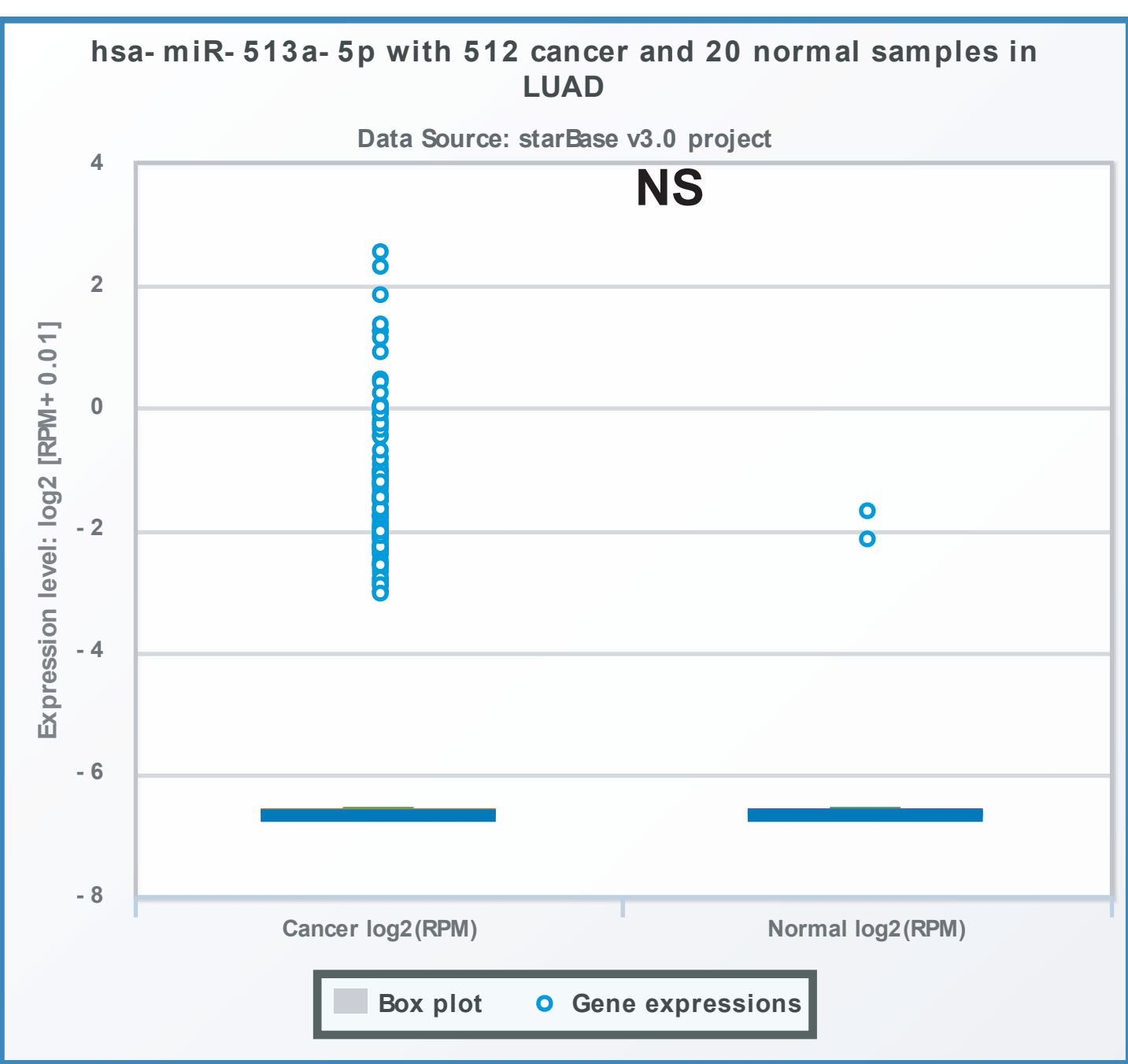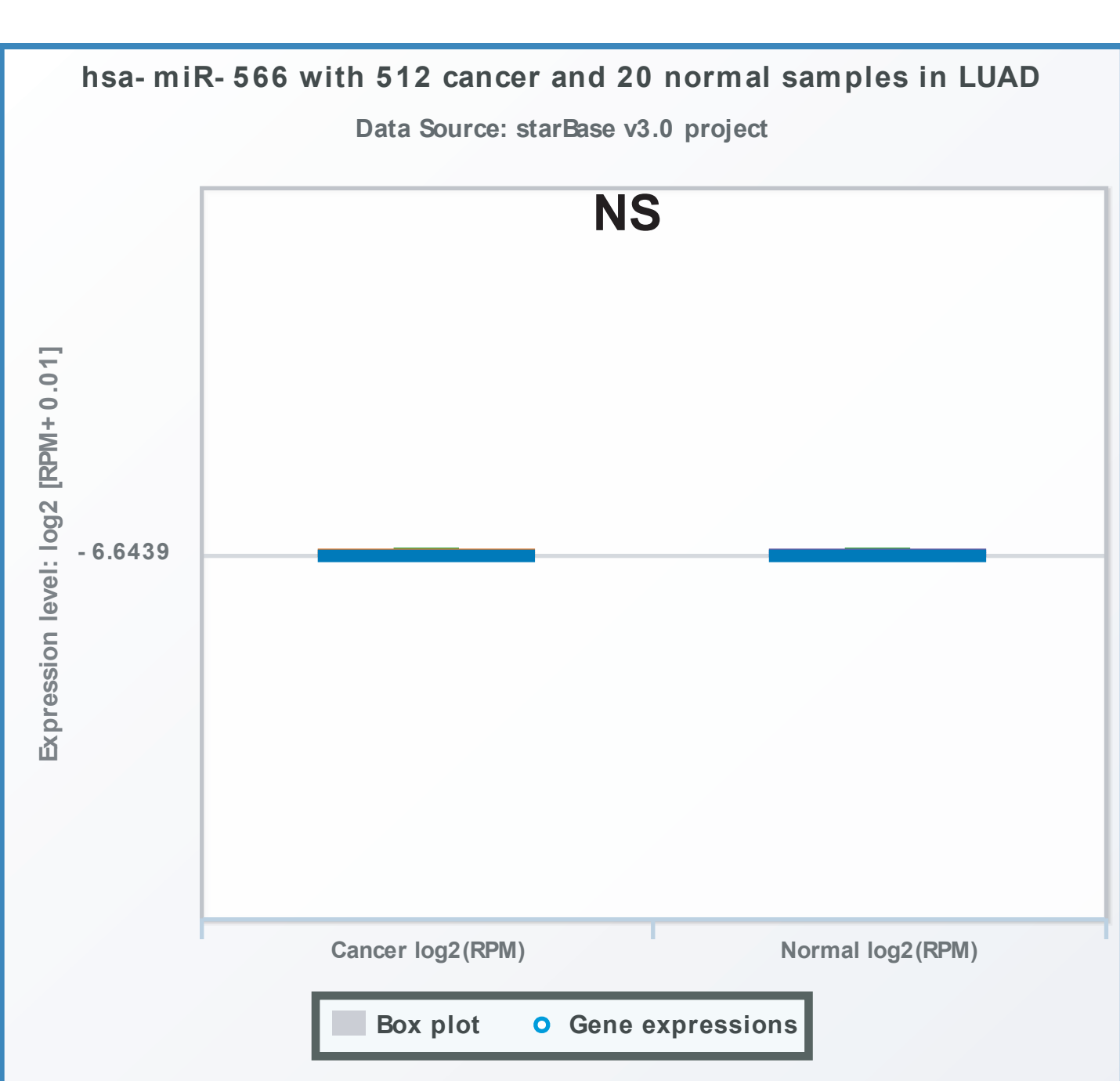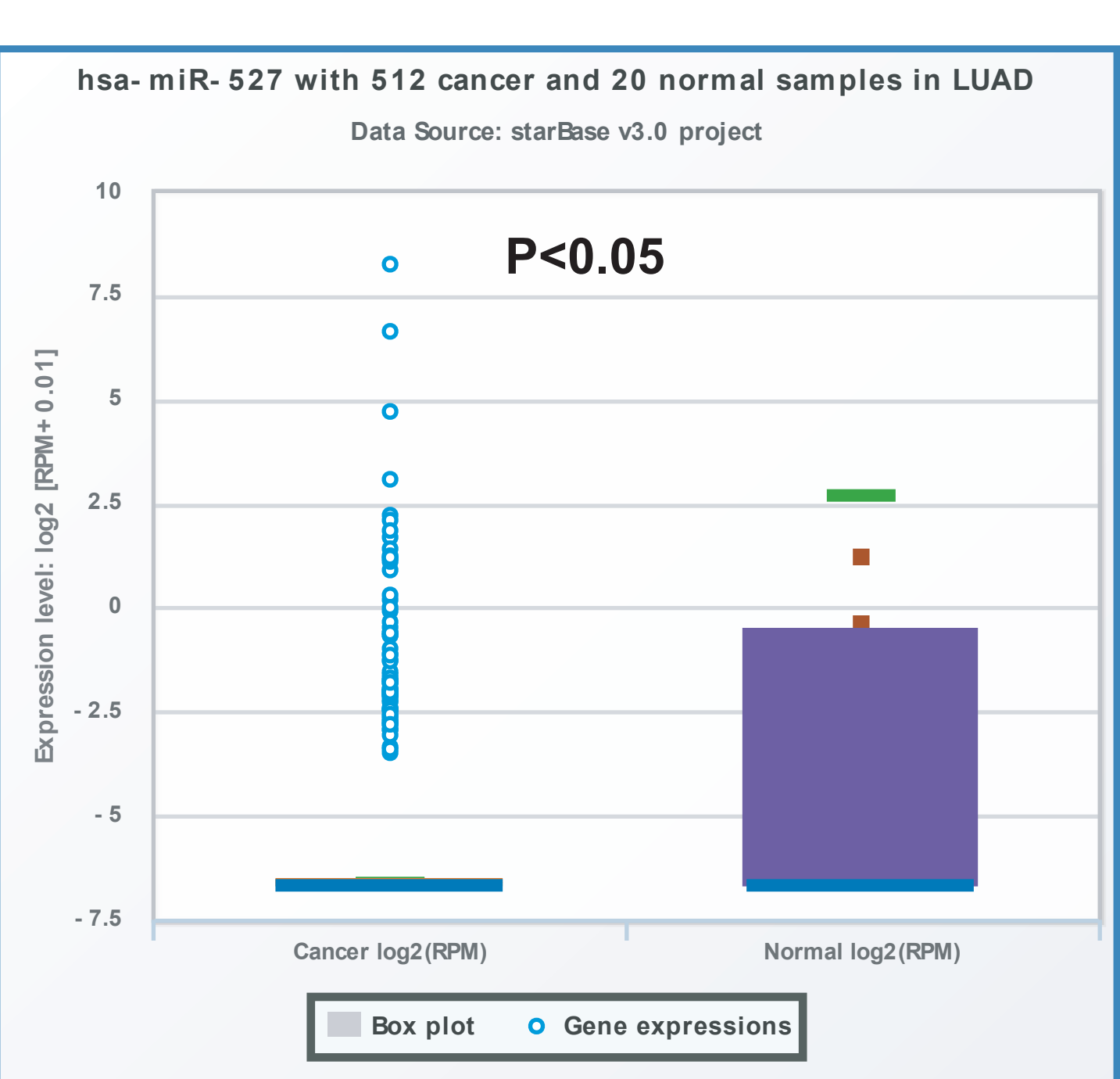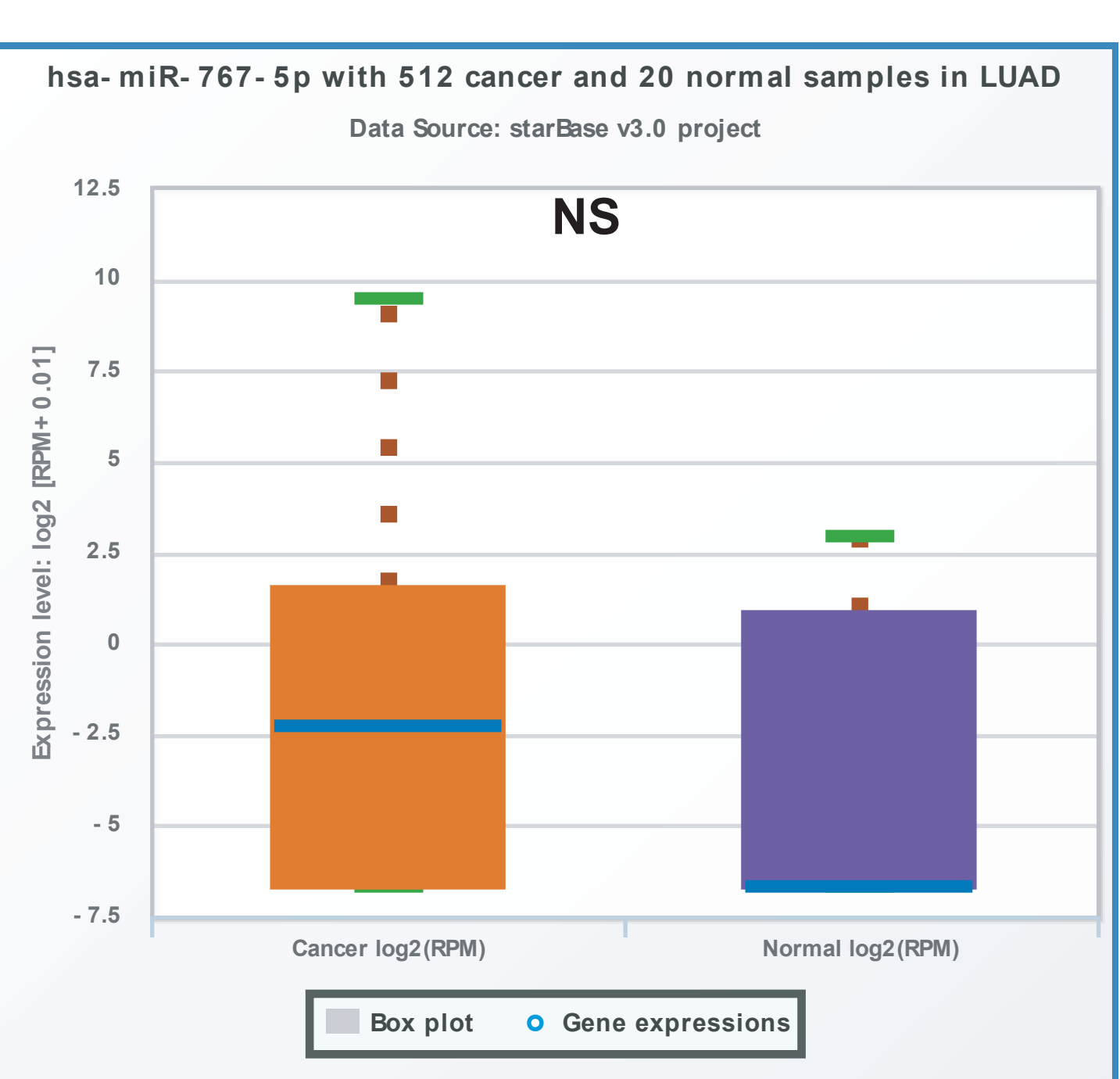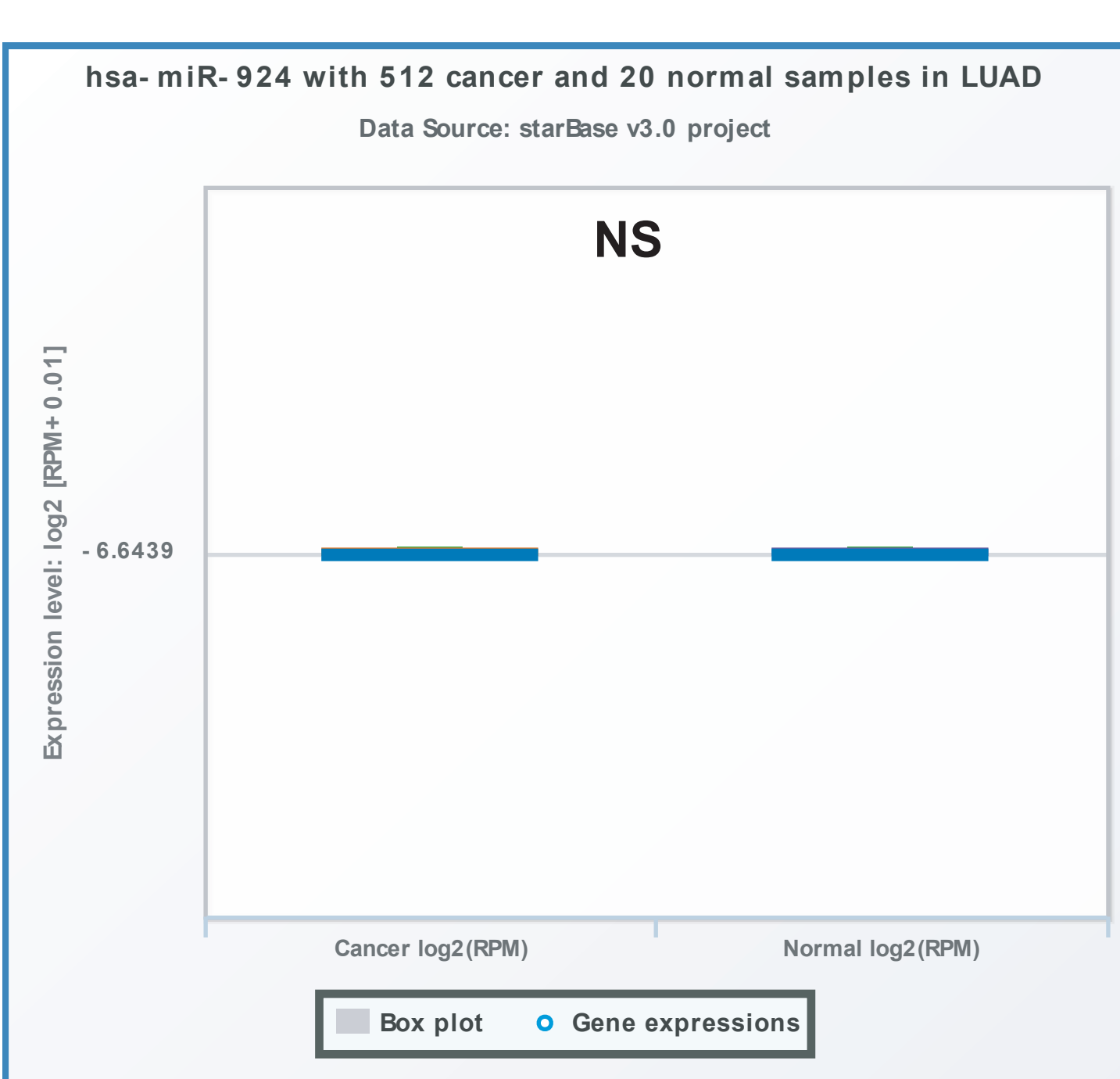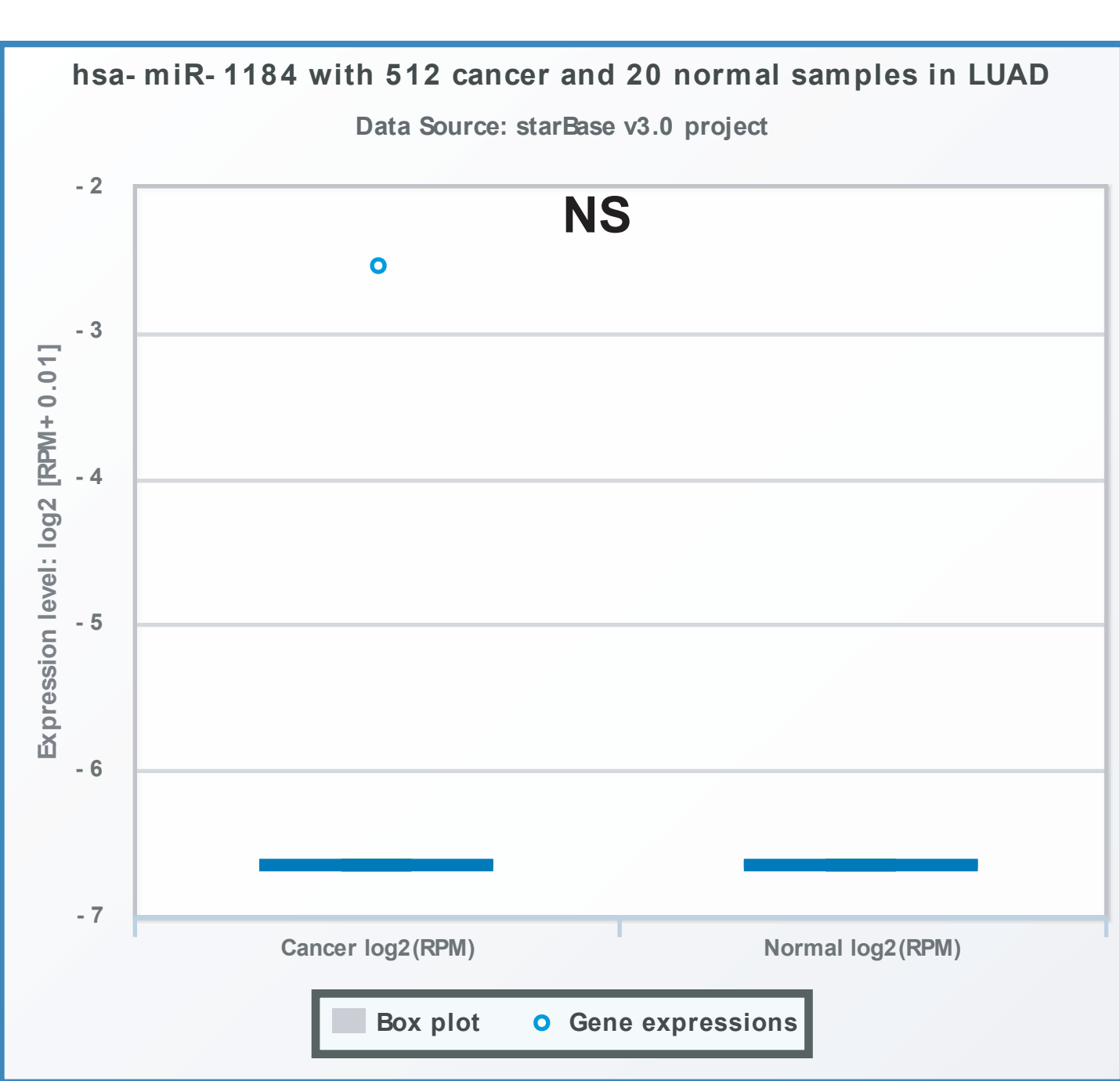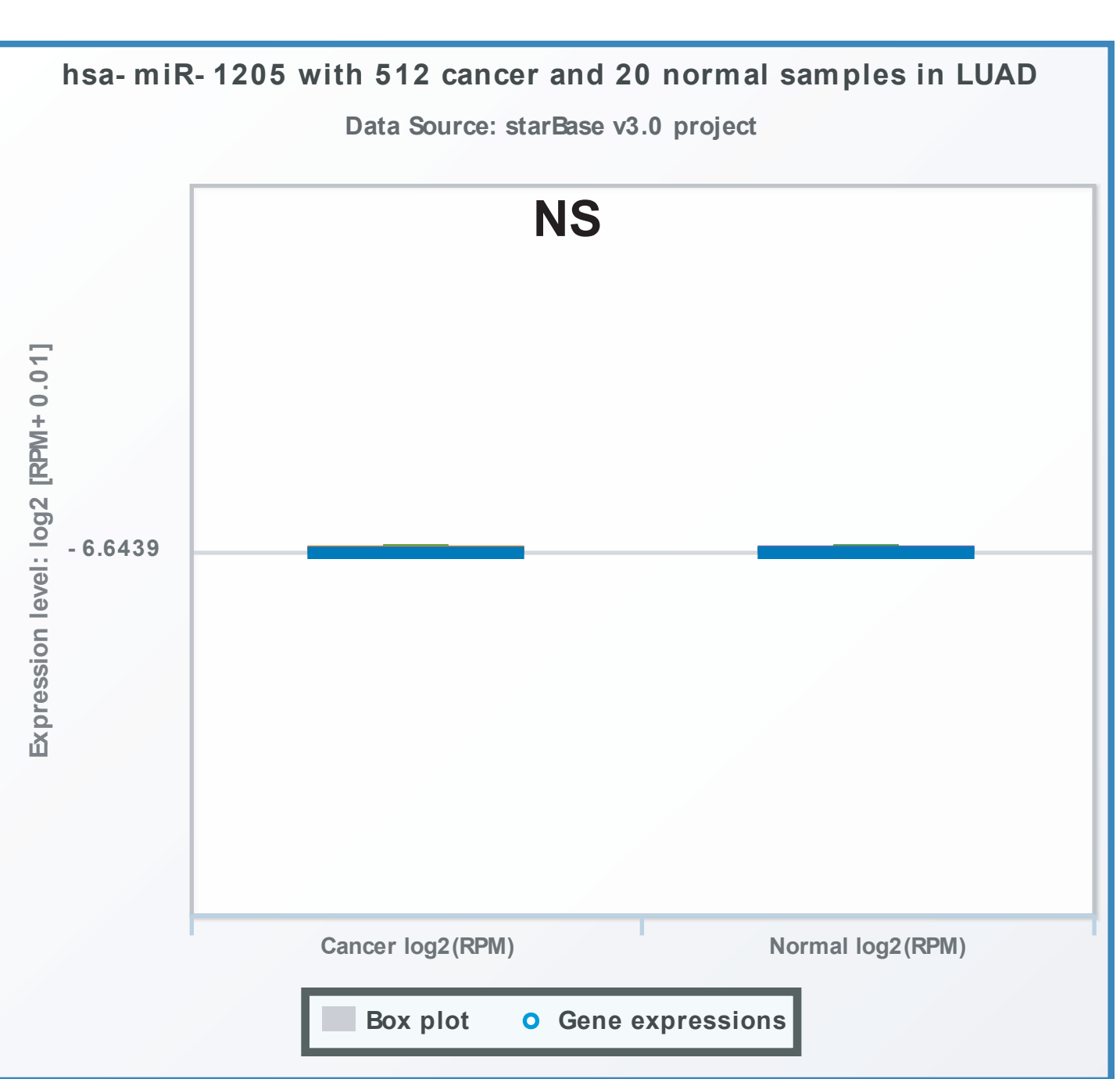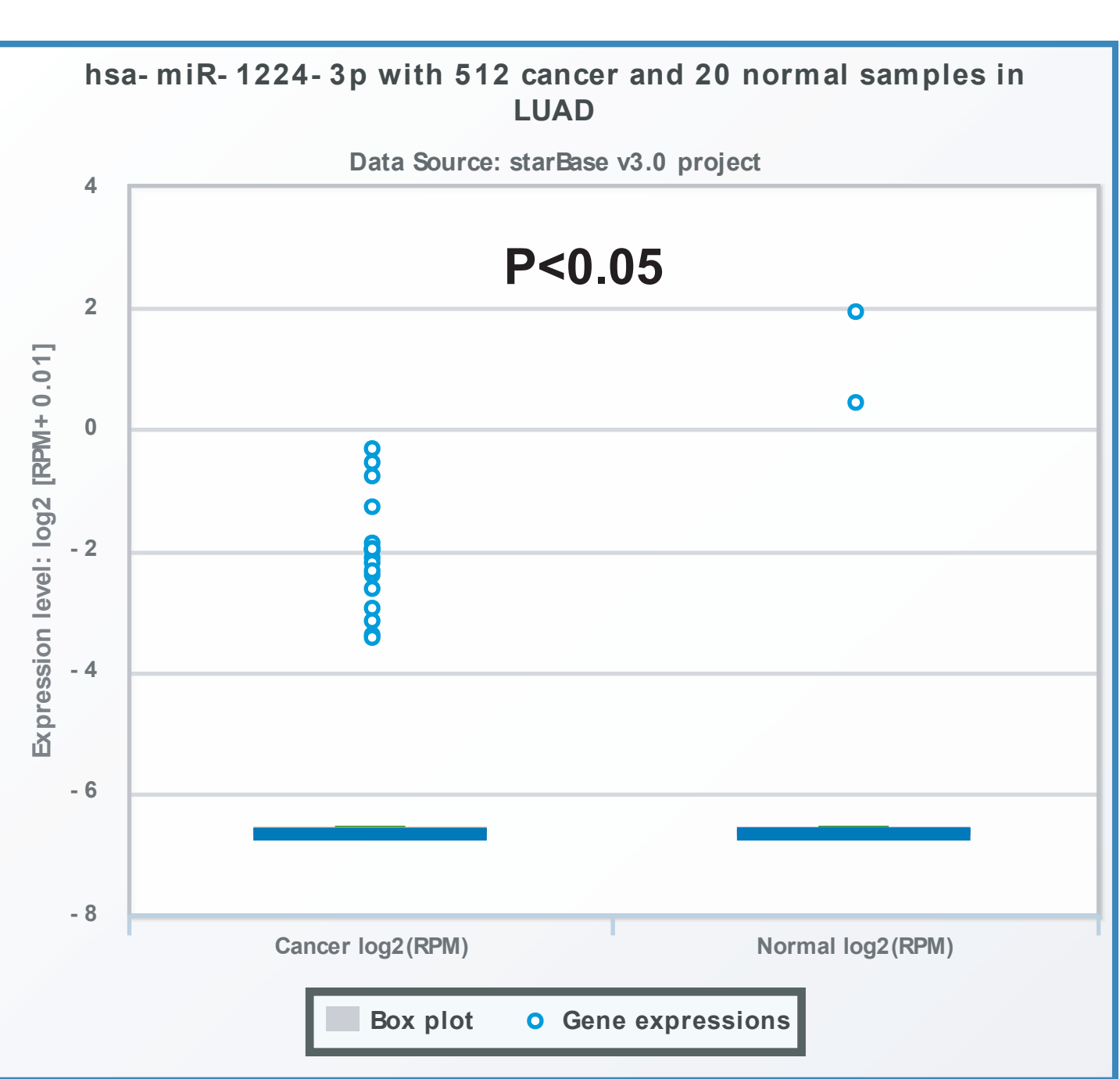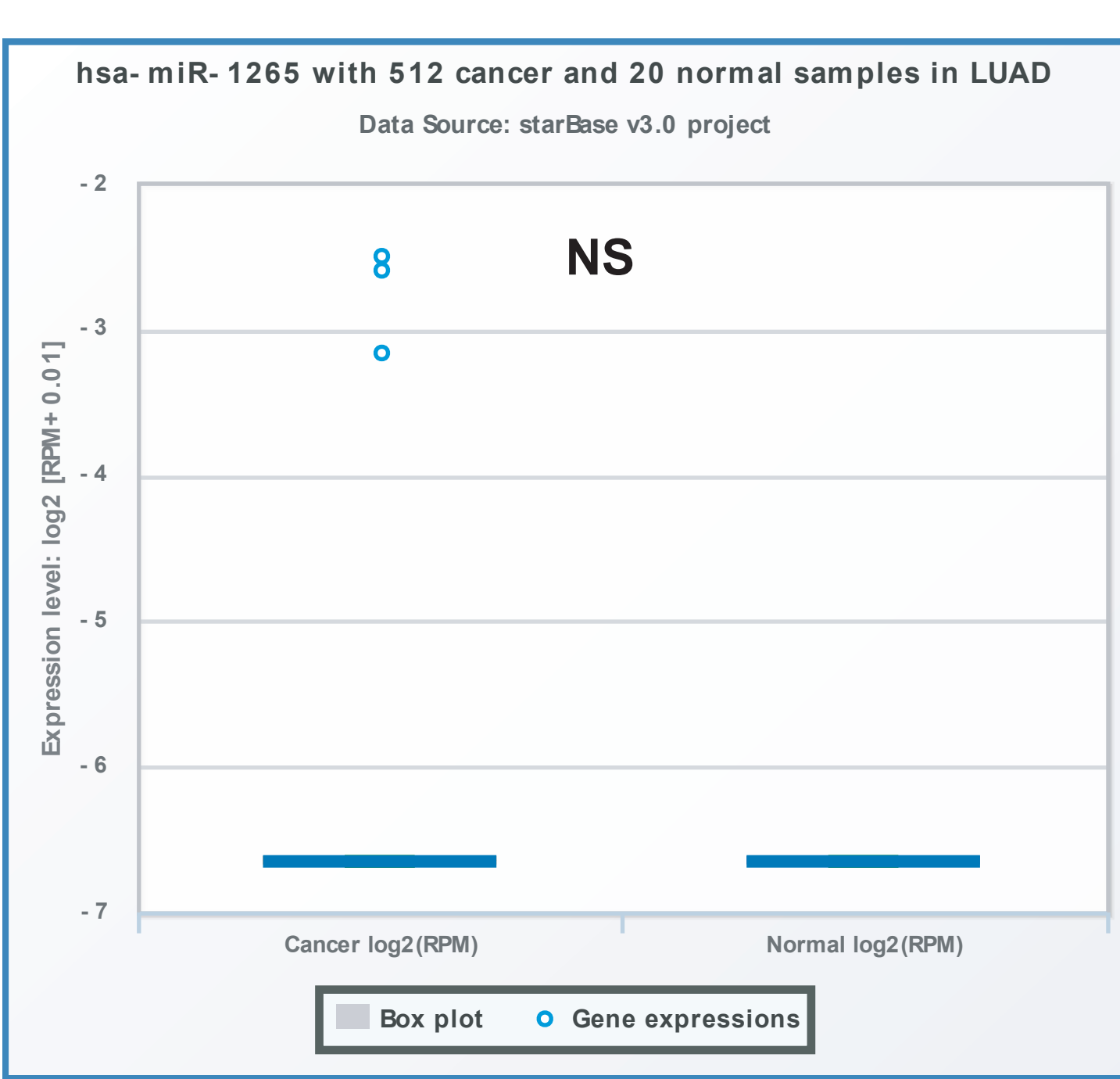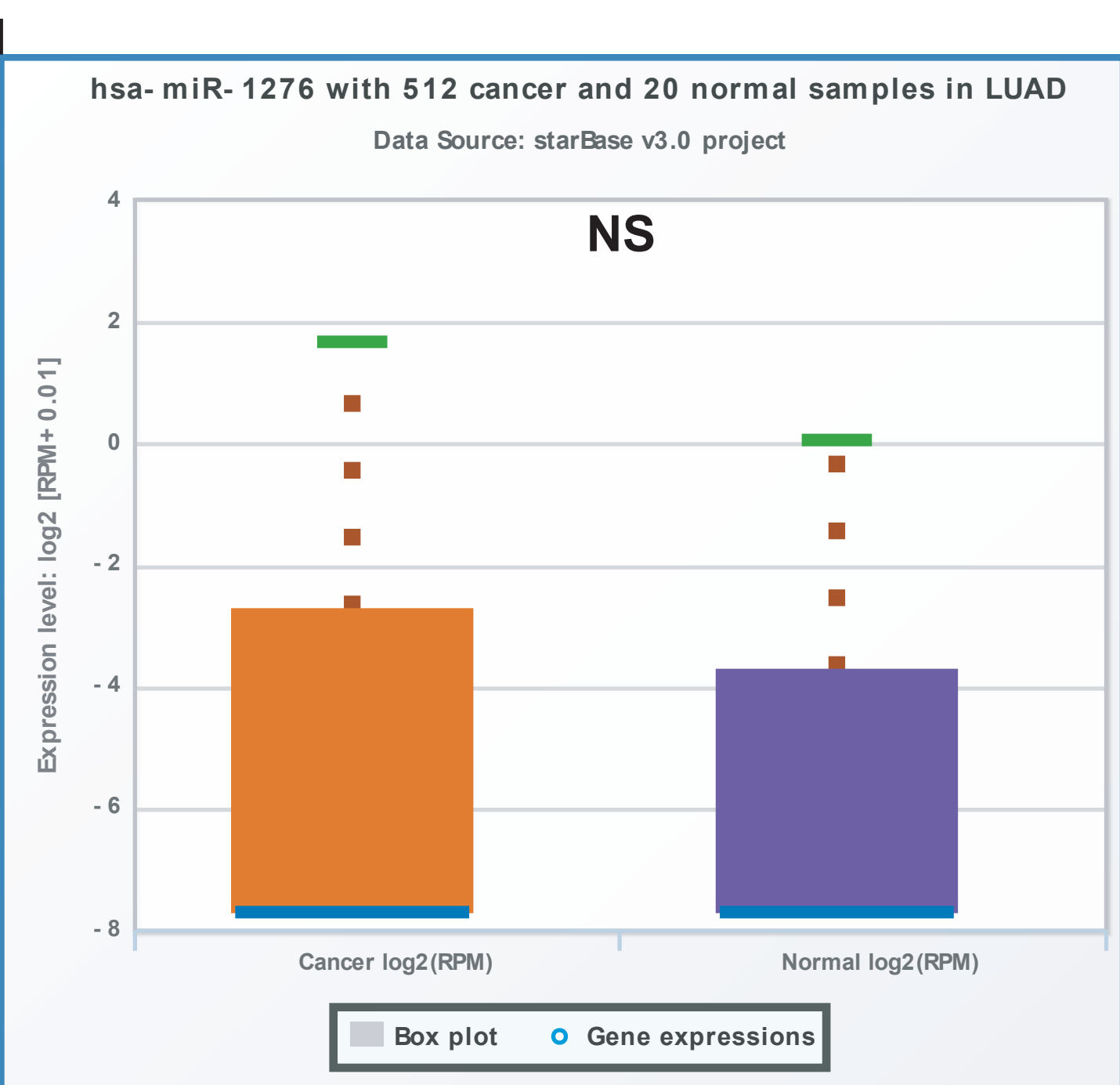

Supplement: Supplementary file 1 — Additional file 1:Fig. S1 Expression level of miRNAs in LUAD (a-m). The expression of miRNAs was analyzed by using starBase in LUAD. LUAD, lung adenocarcinoma. [file 12935_2021_2278_MOESM1_ESM.pdf]

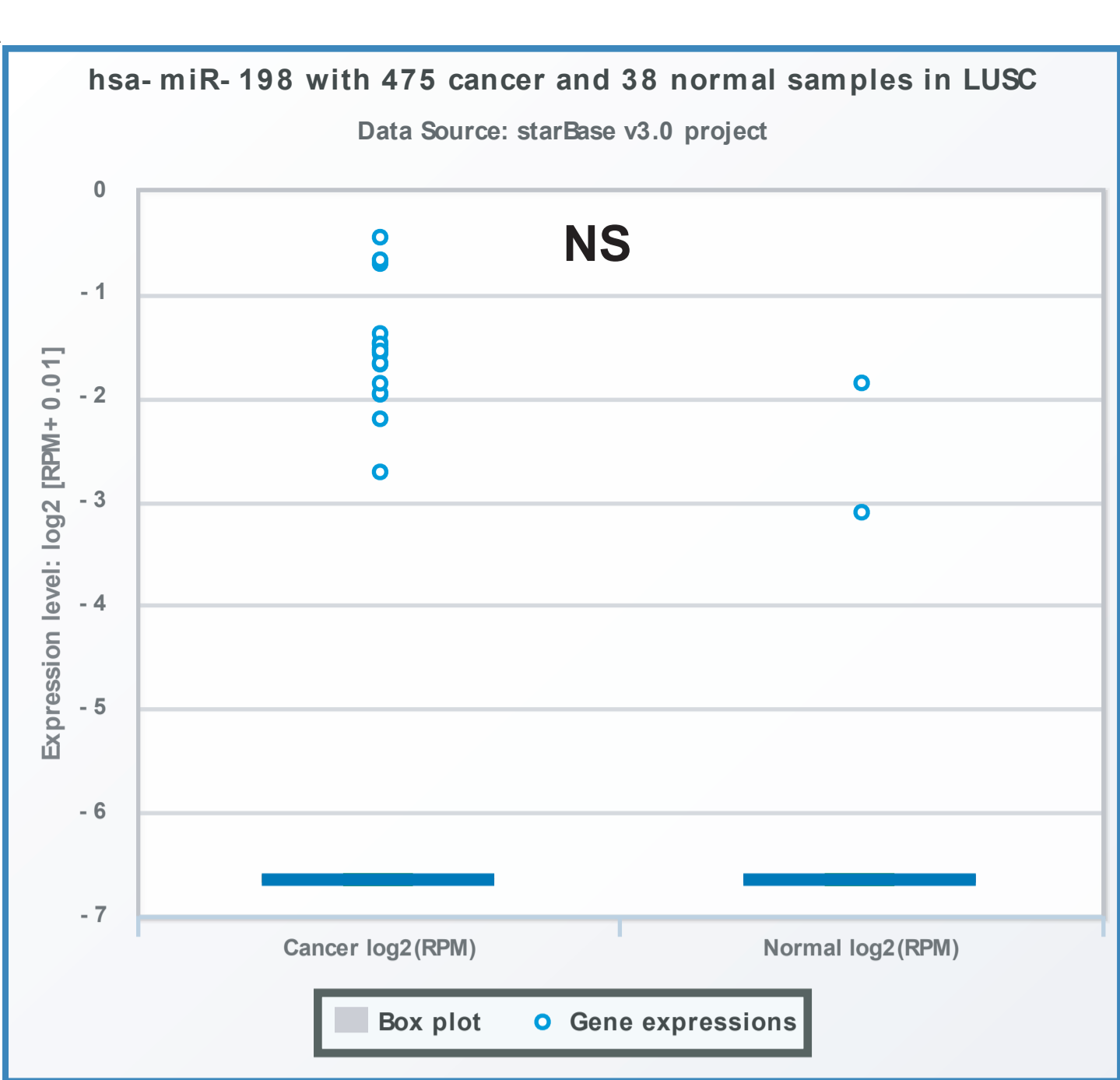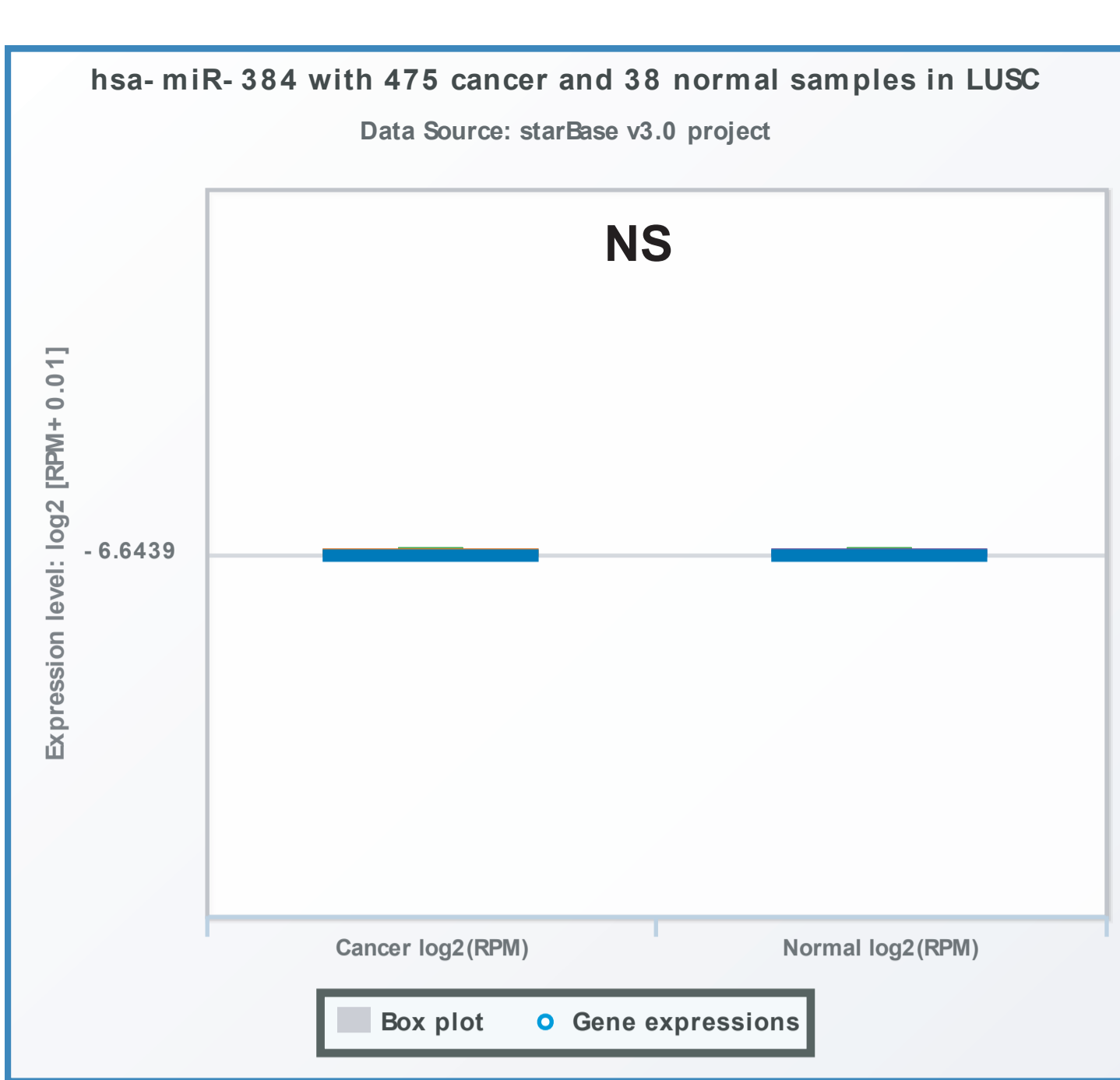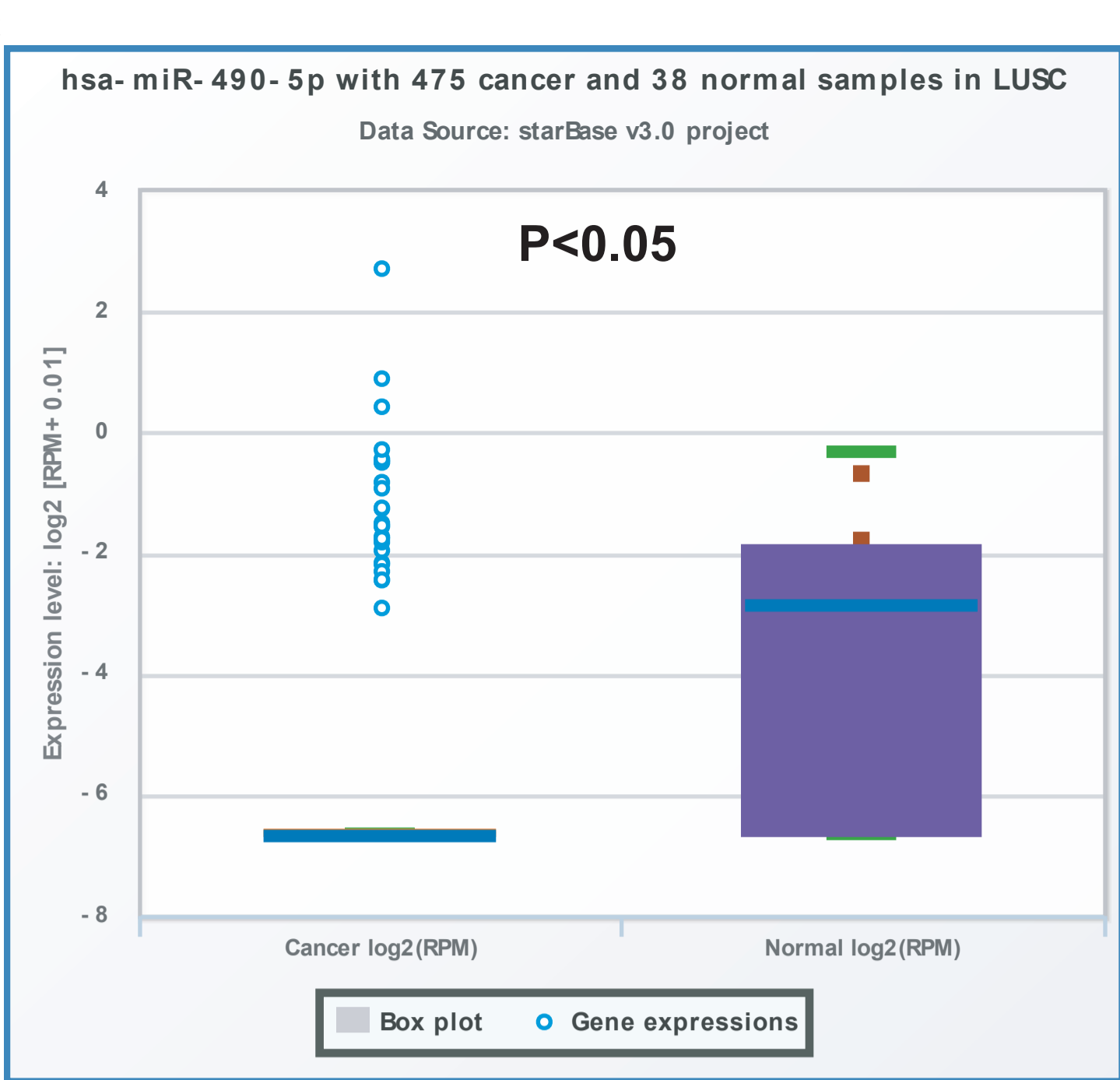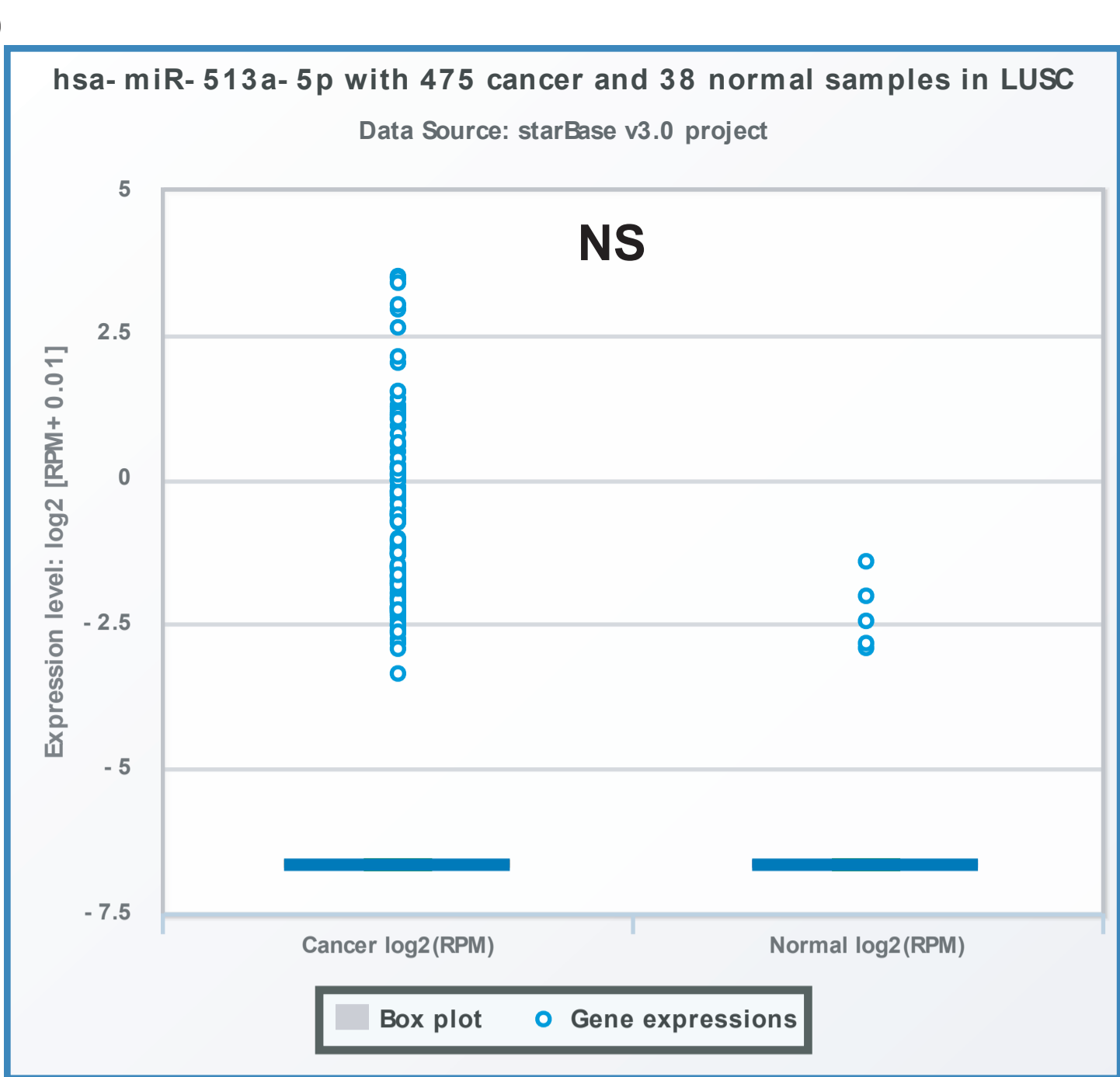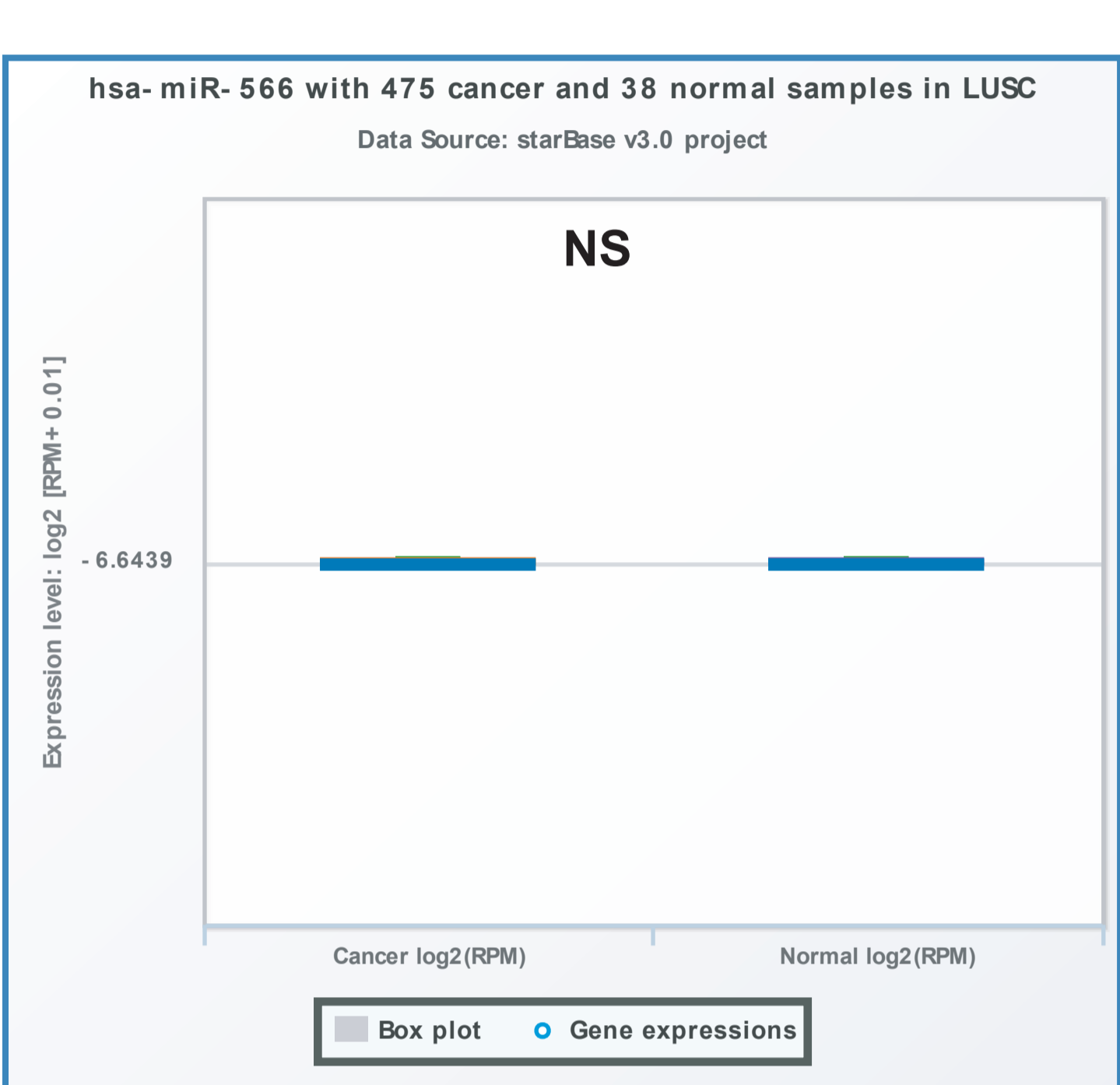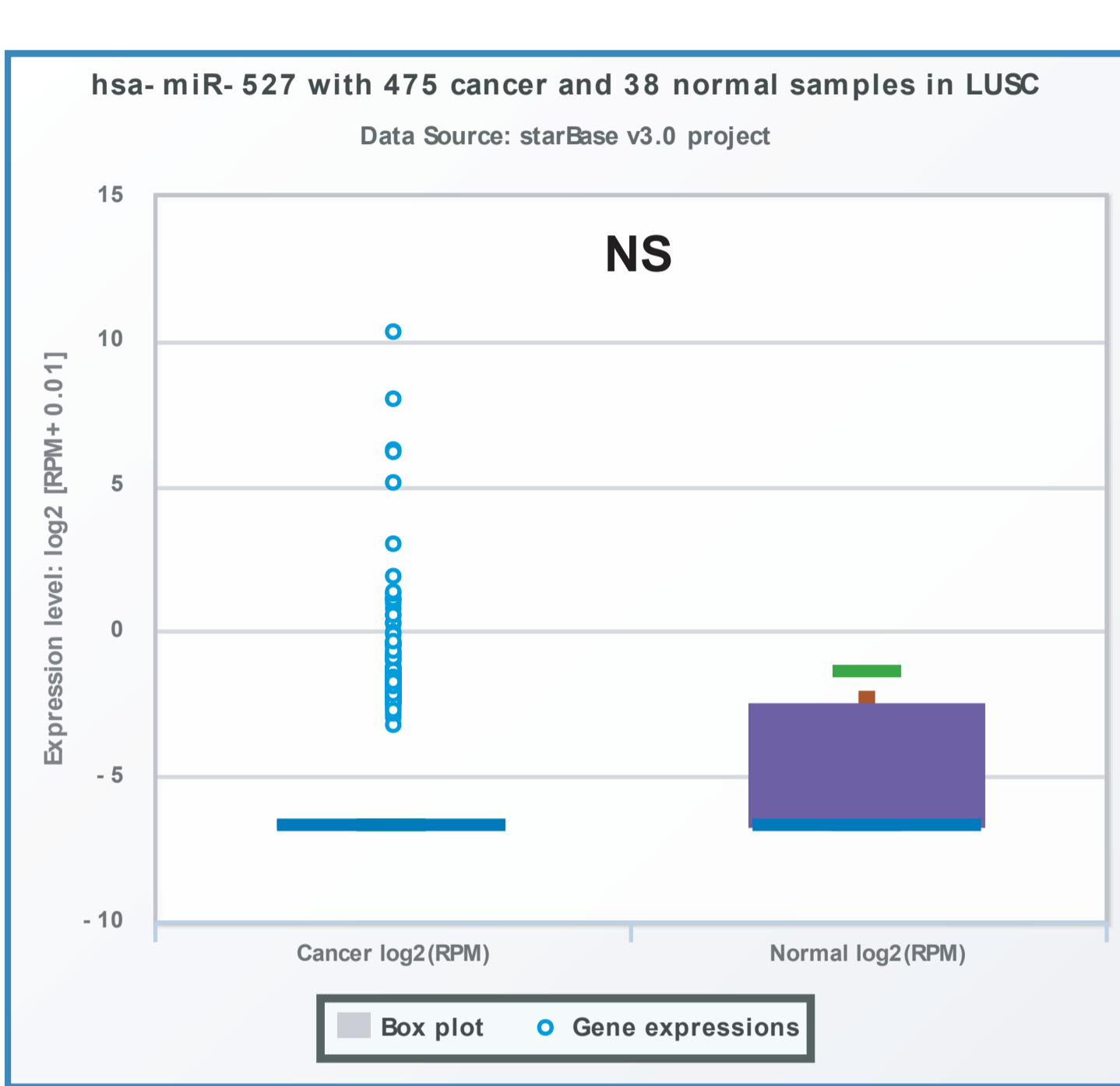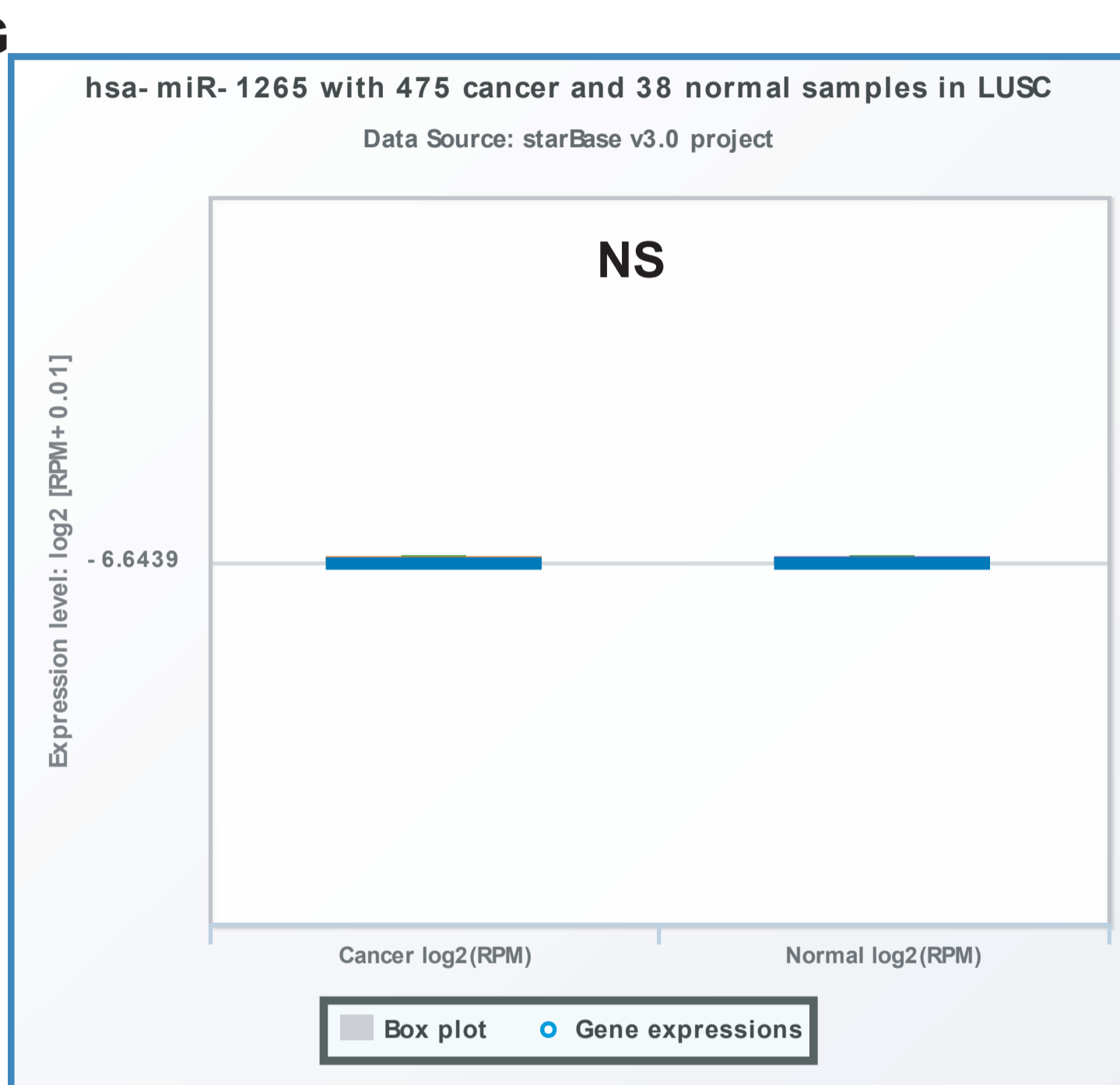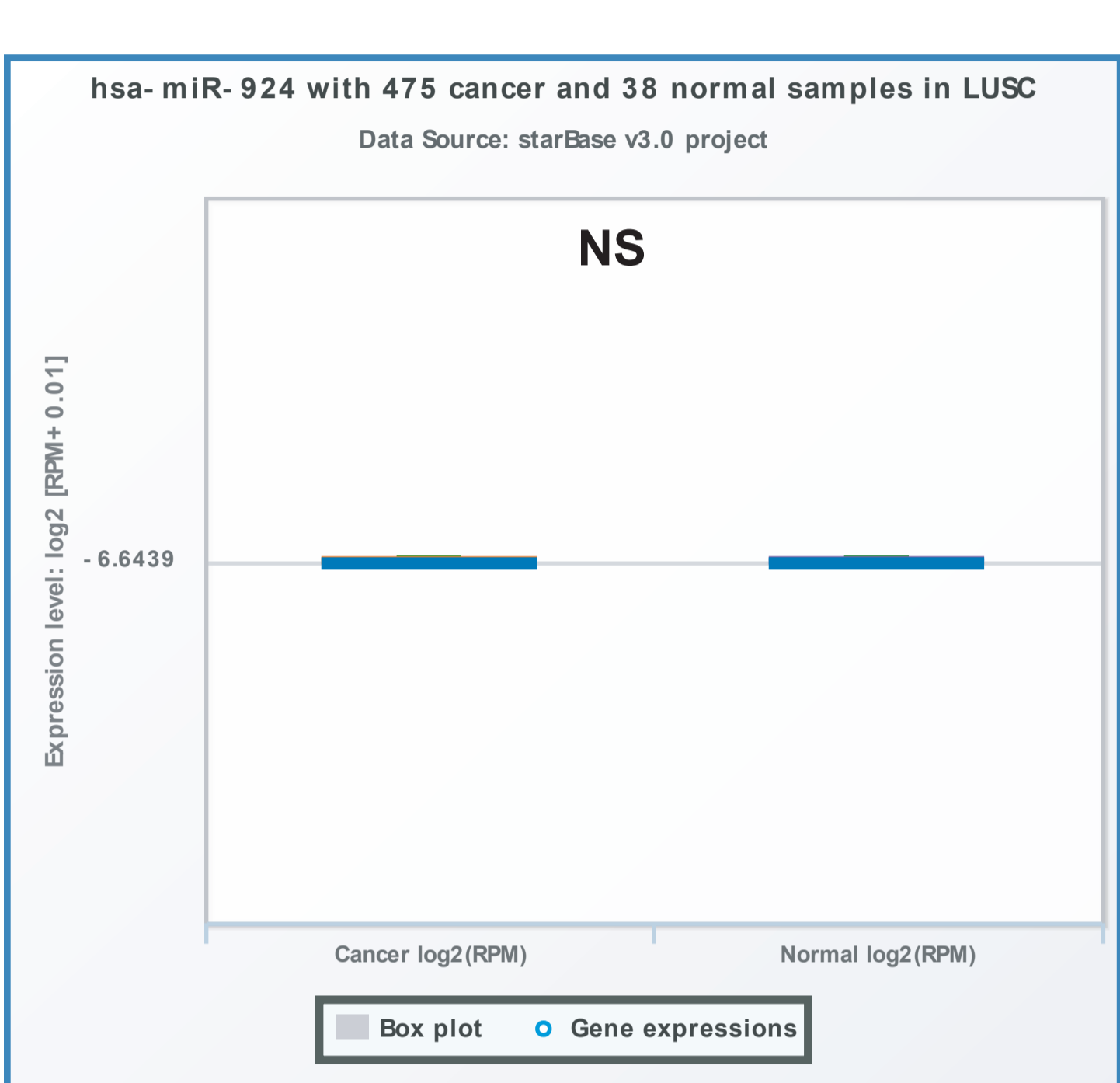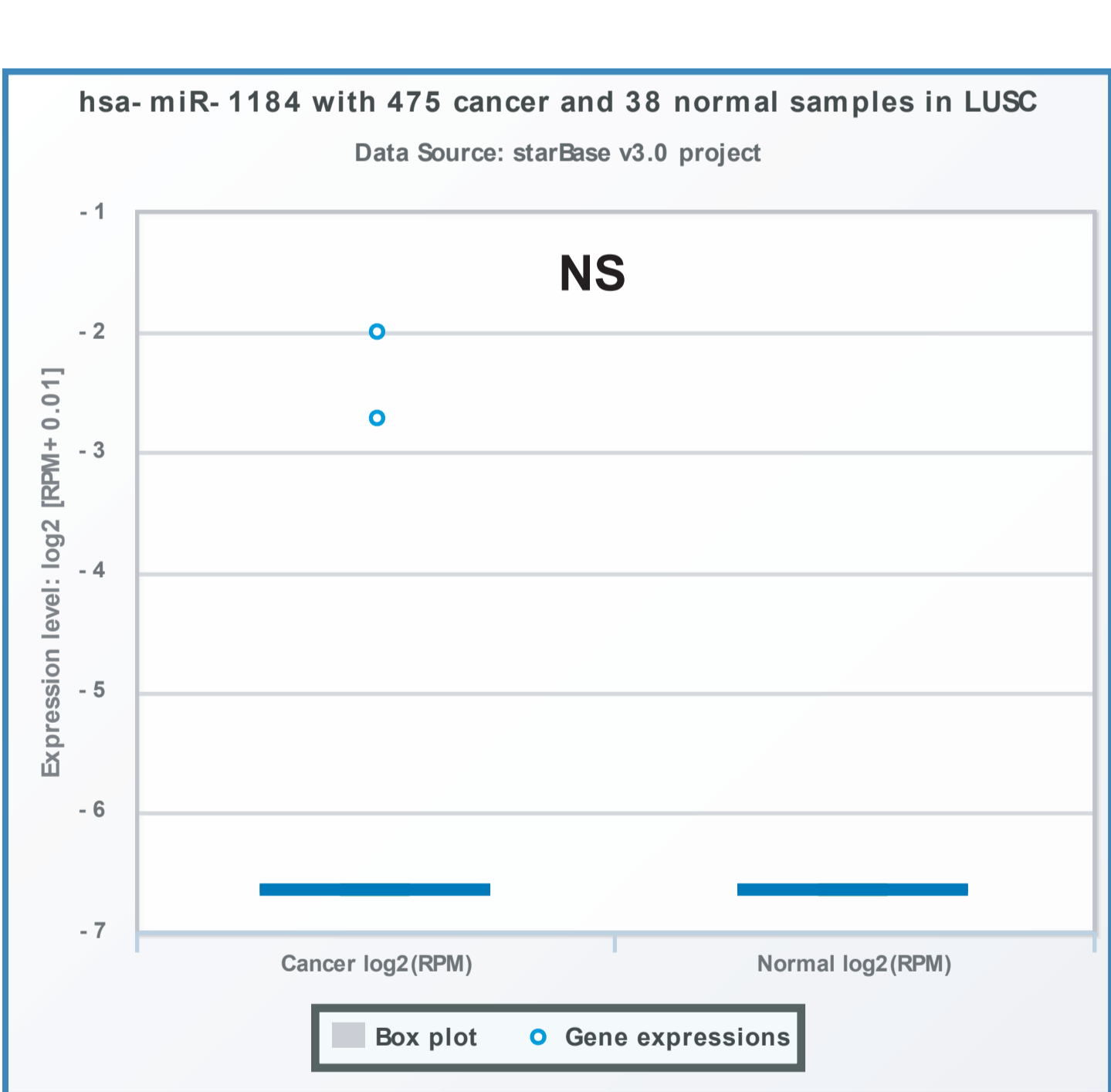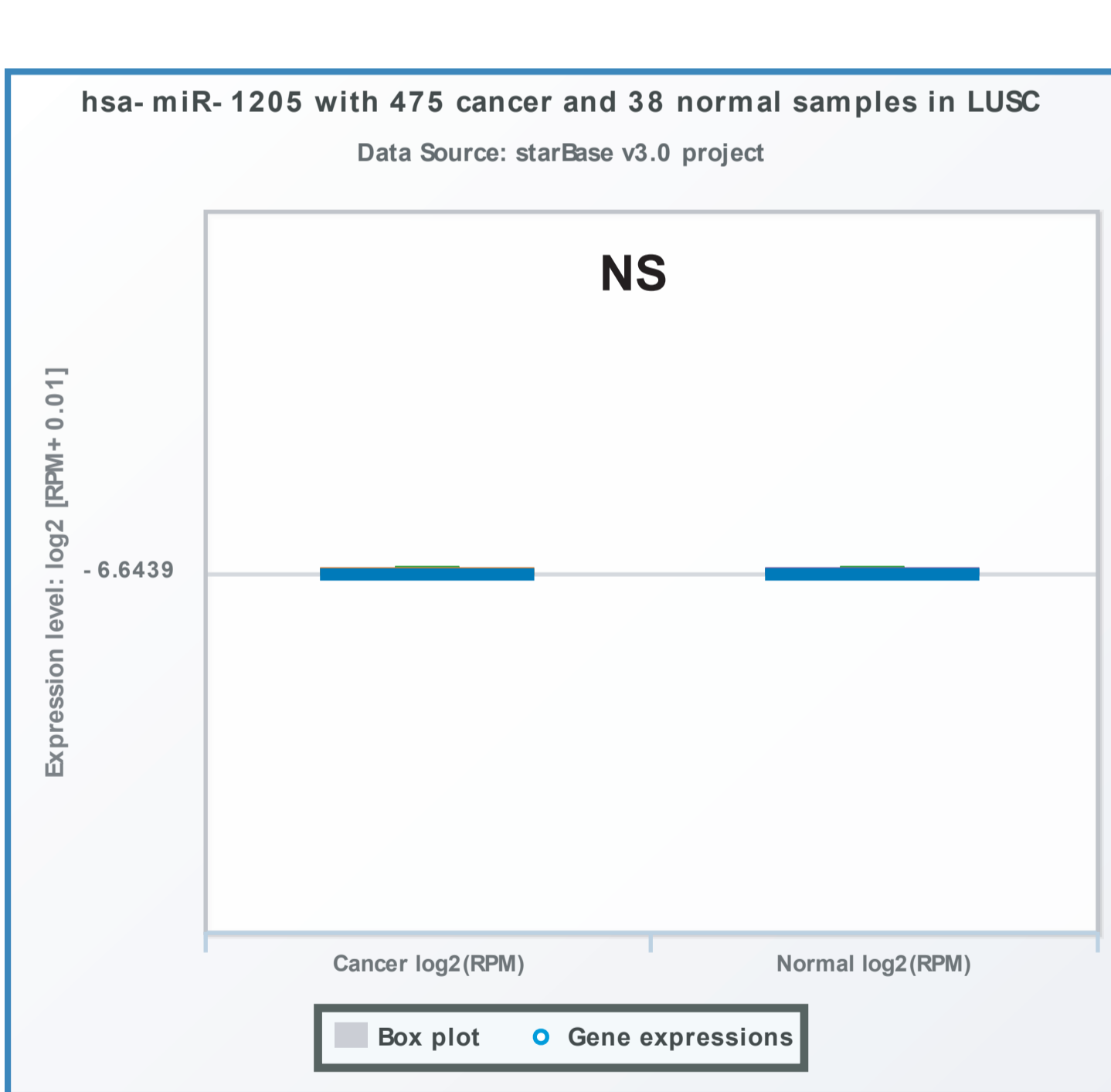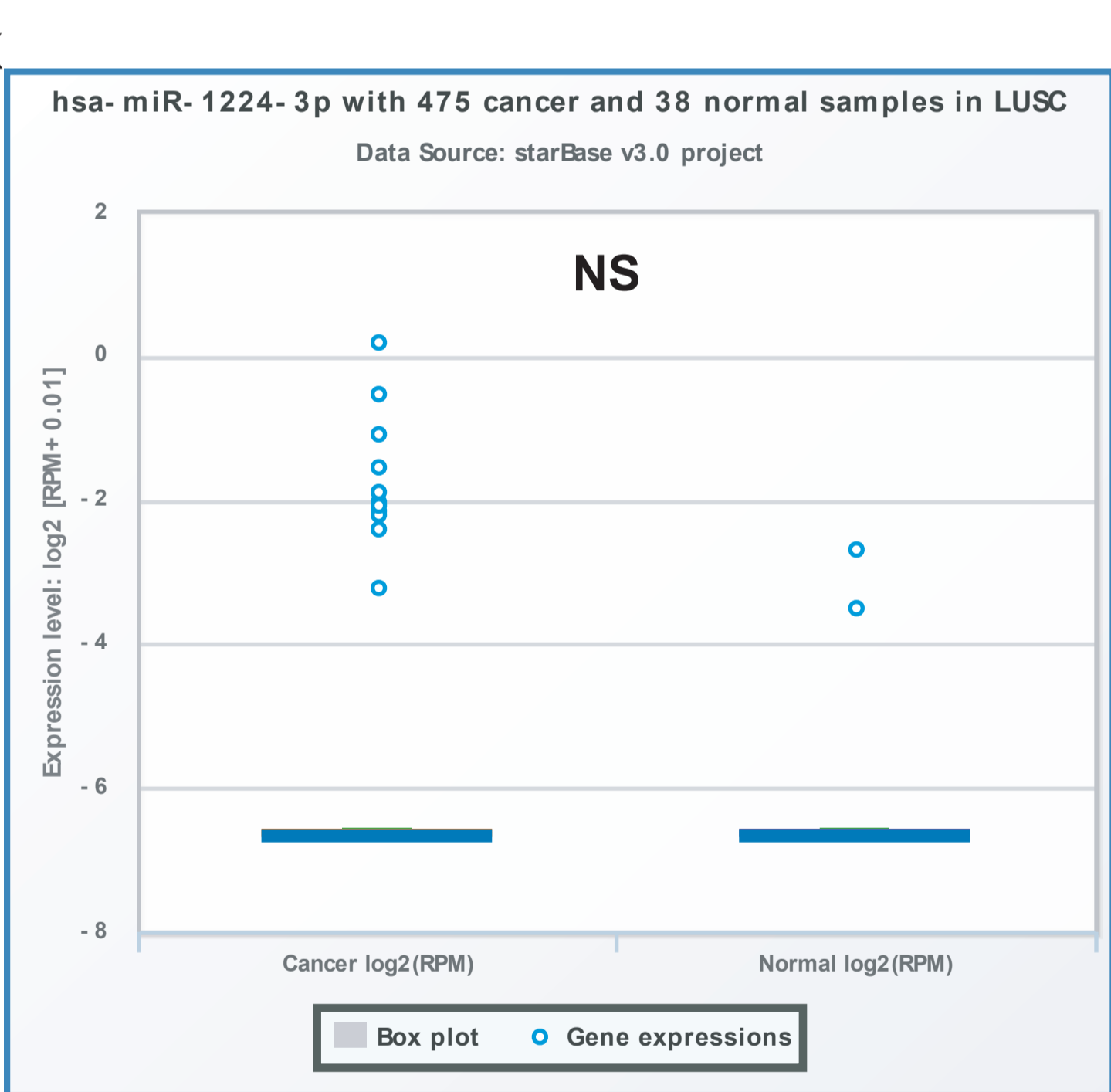

Supplement: Supplementary file 2 — Additional file 2:Fig. S2 Expression level of miRNAs in LUSC (a-k). The expression of miRNAs was analyzed by using starBase in LUSC. LUSC, lung squamous cell carcinoma. [file 12935_2021_2278_MOESM2_ESM.pdf]

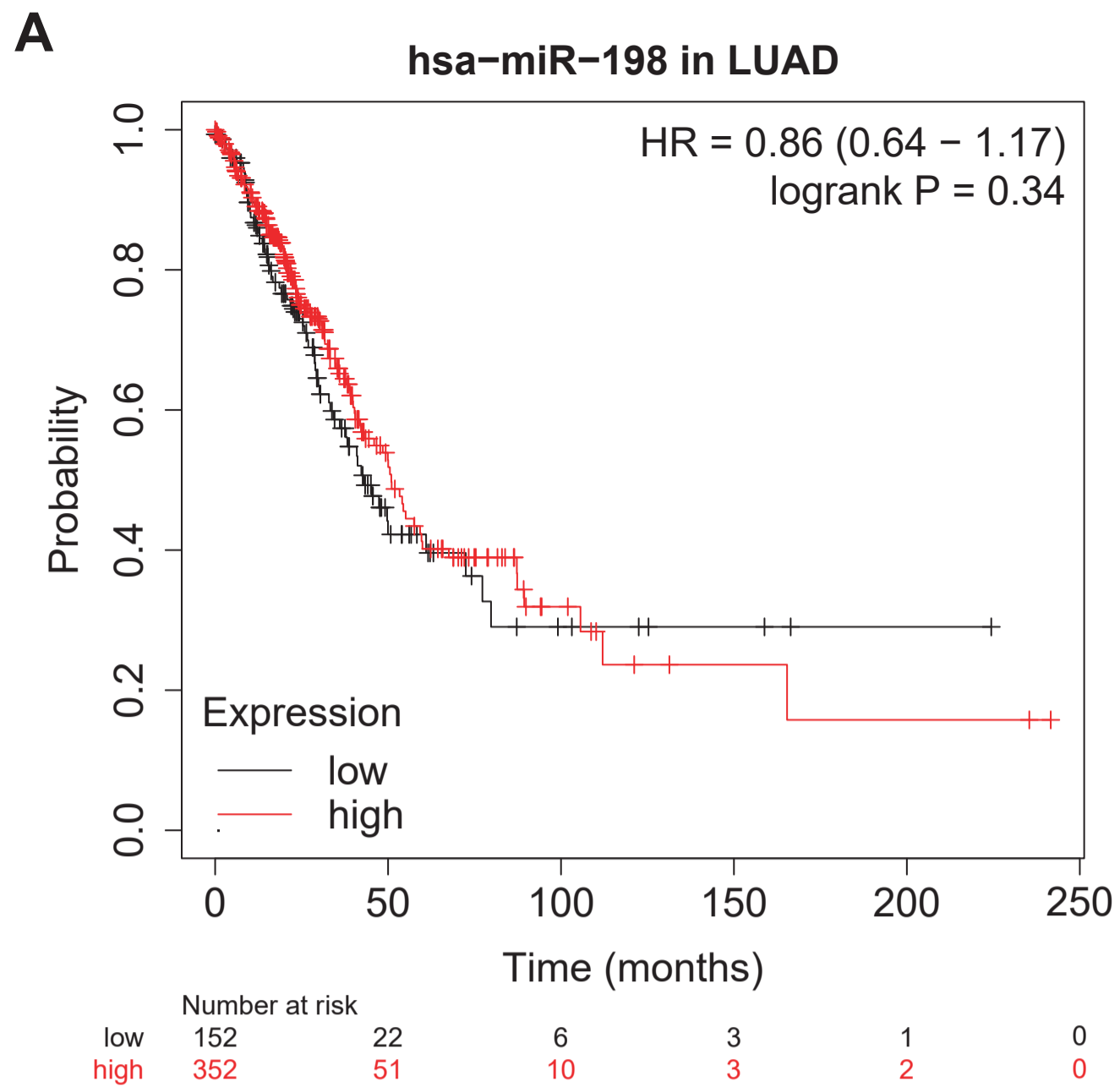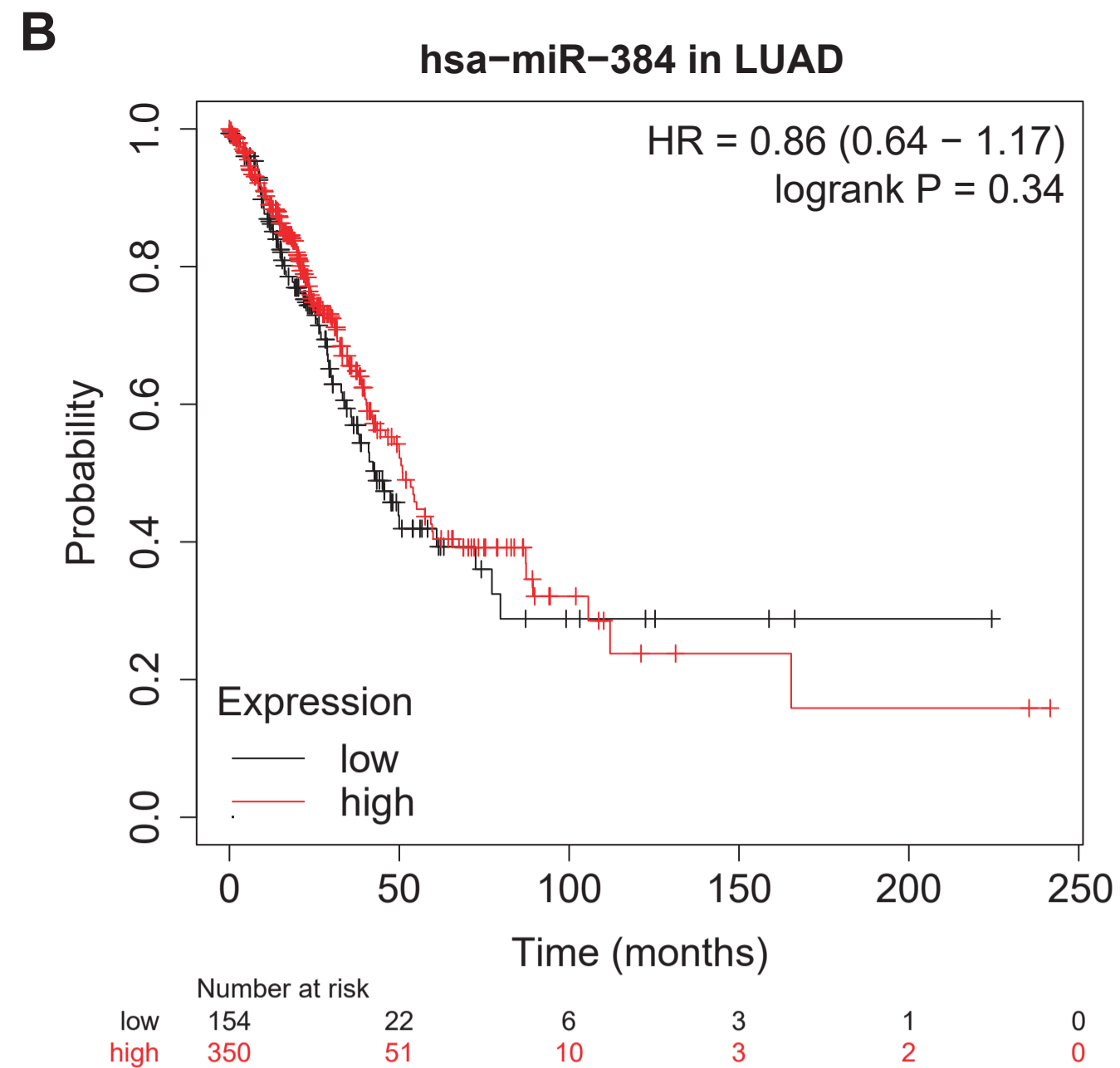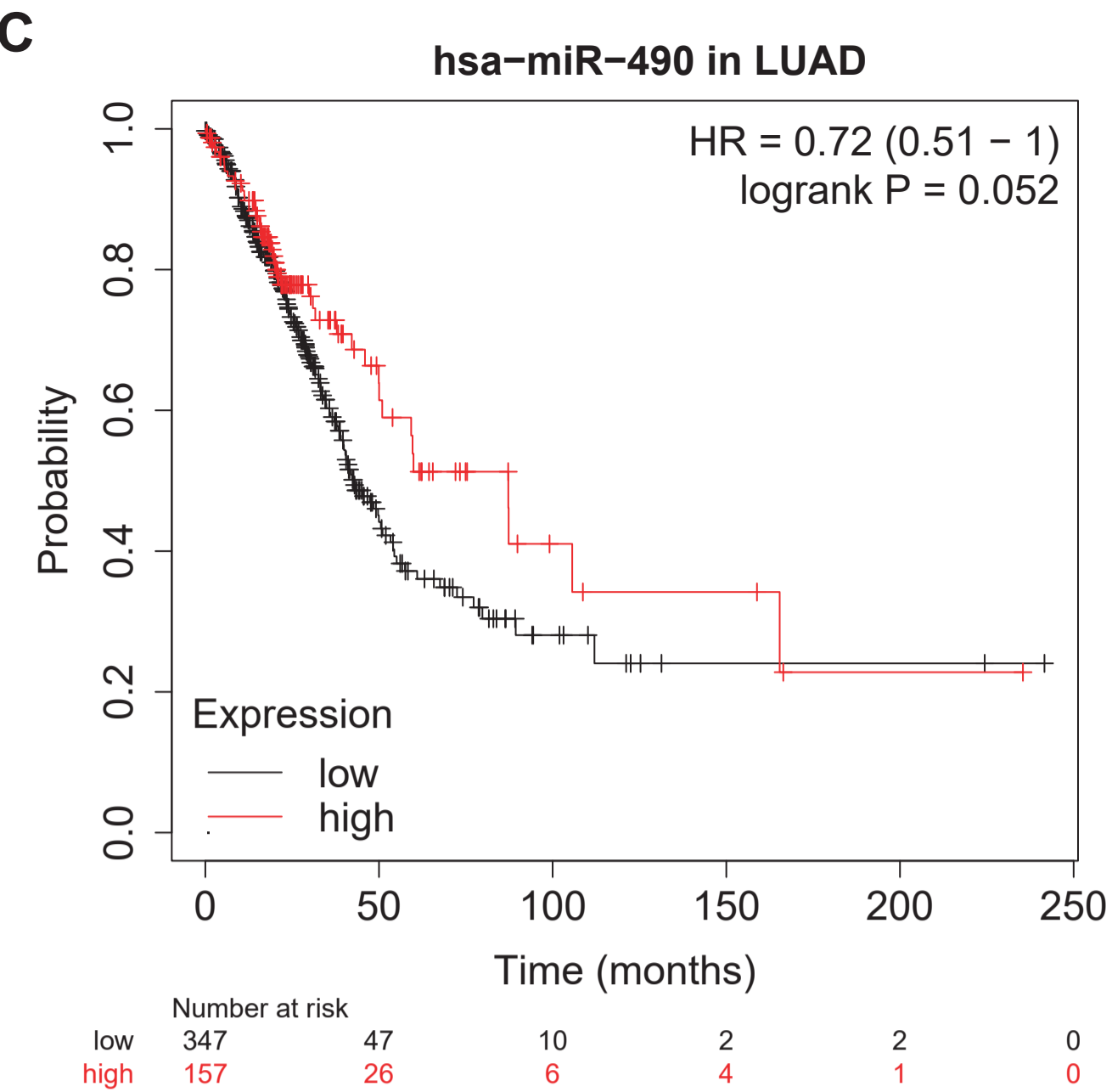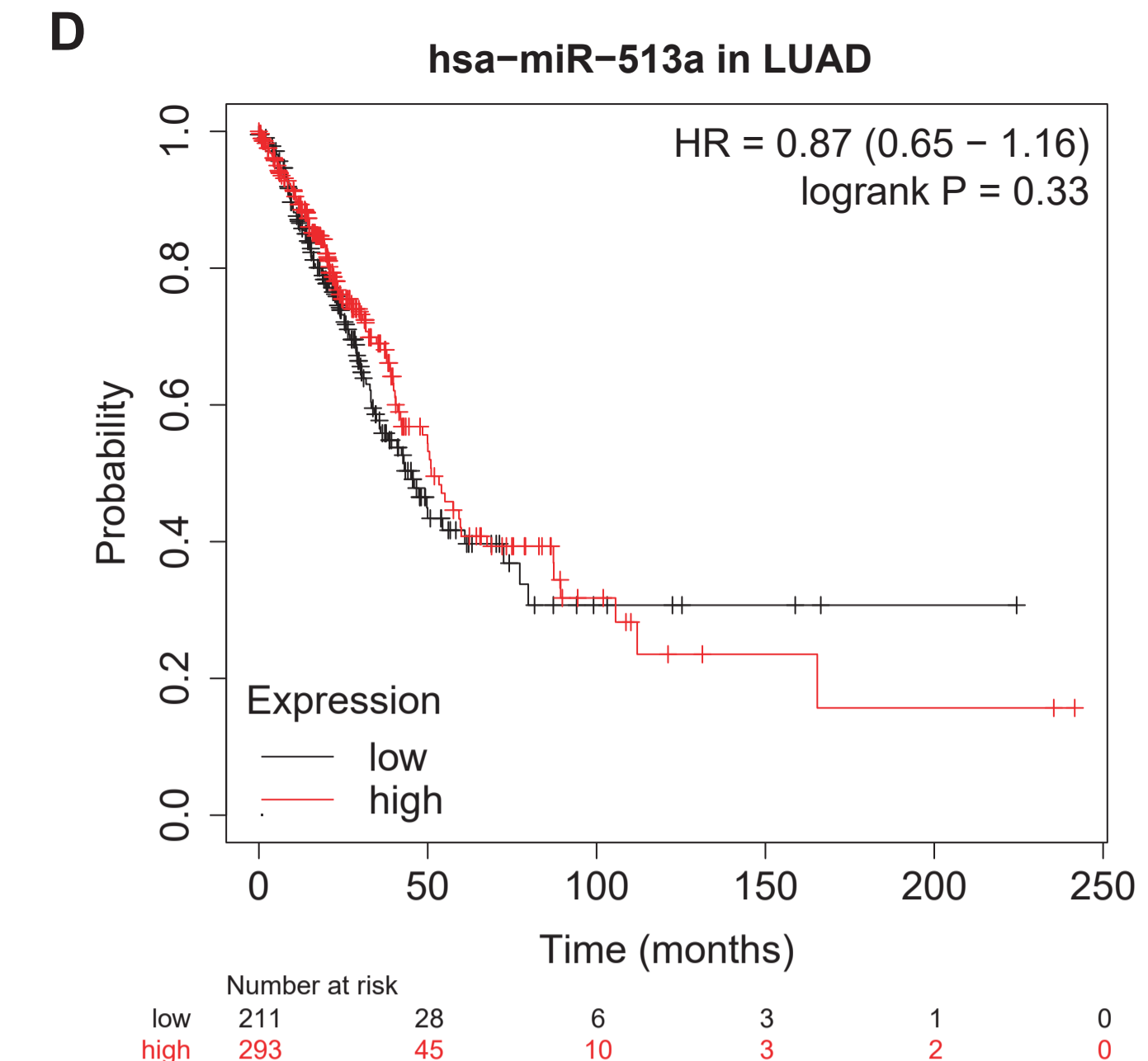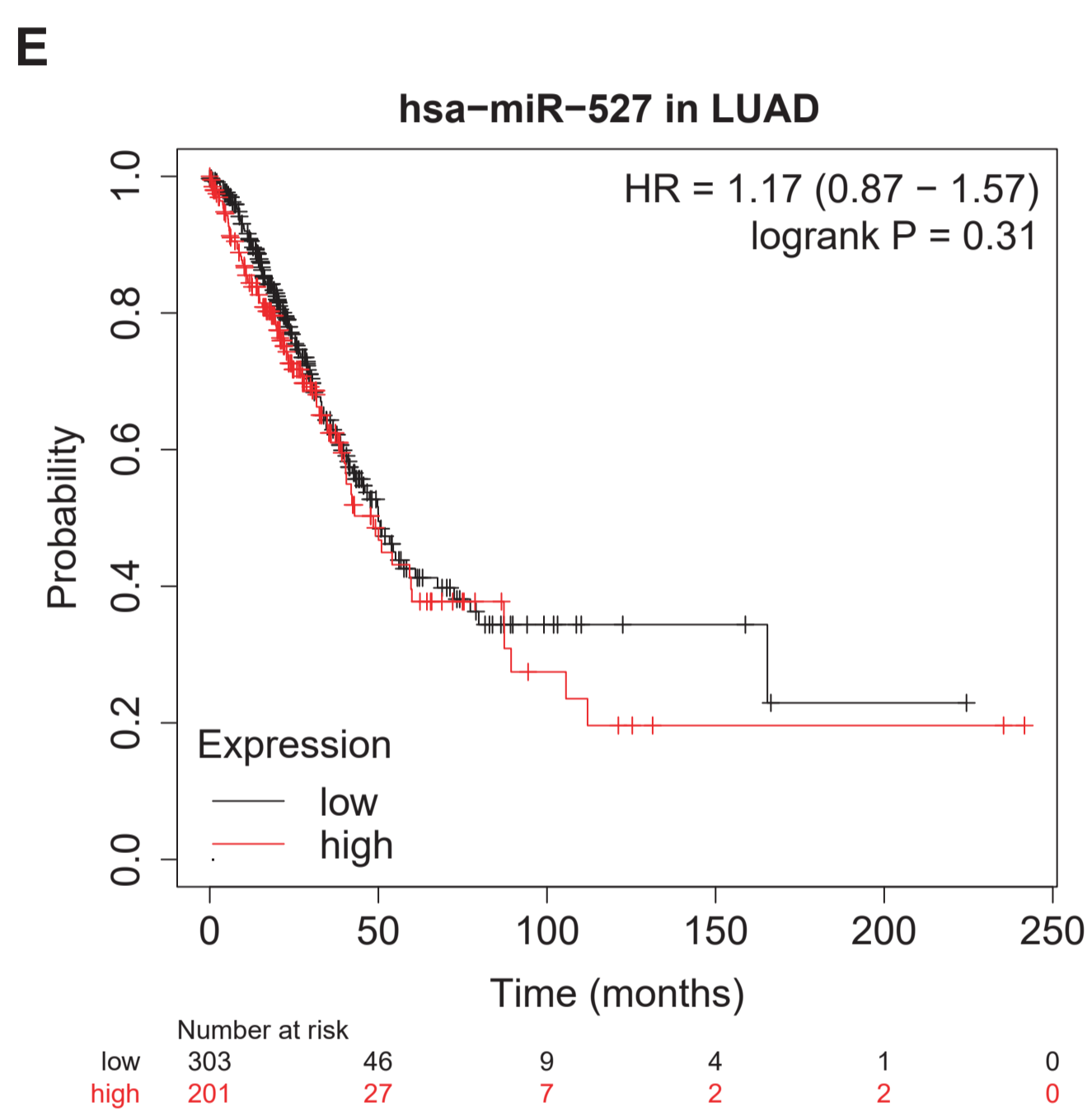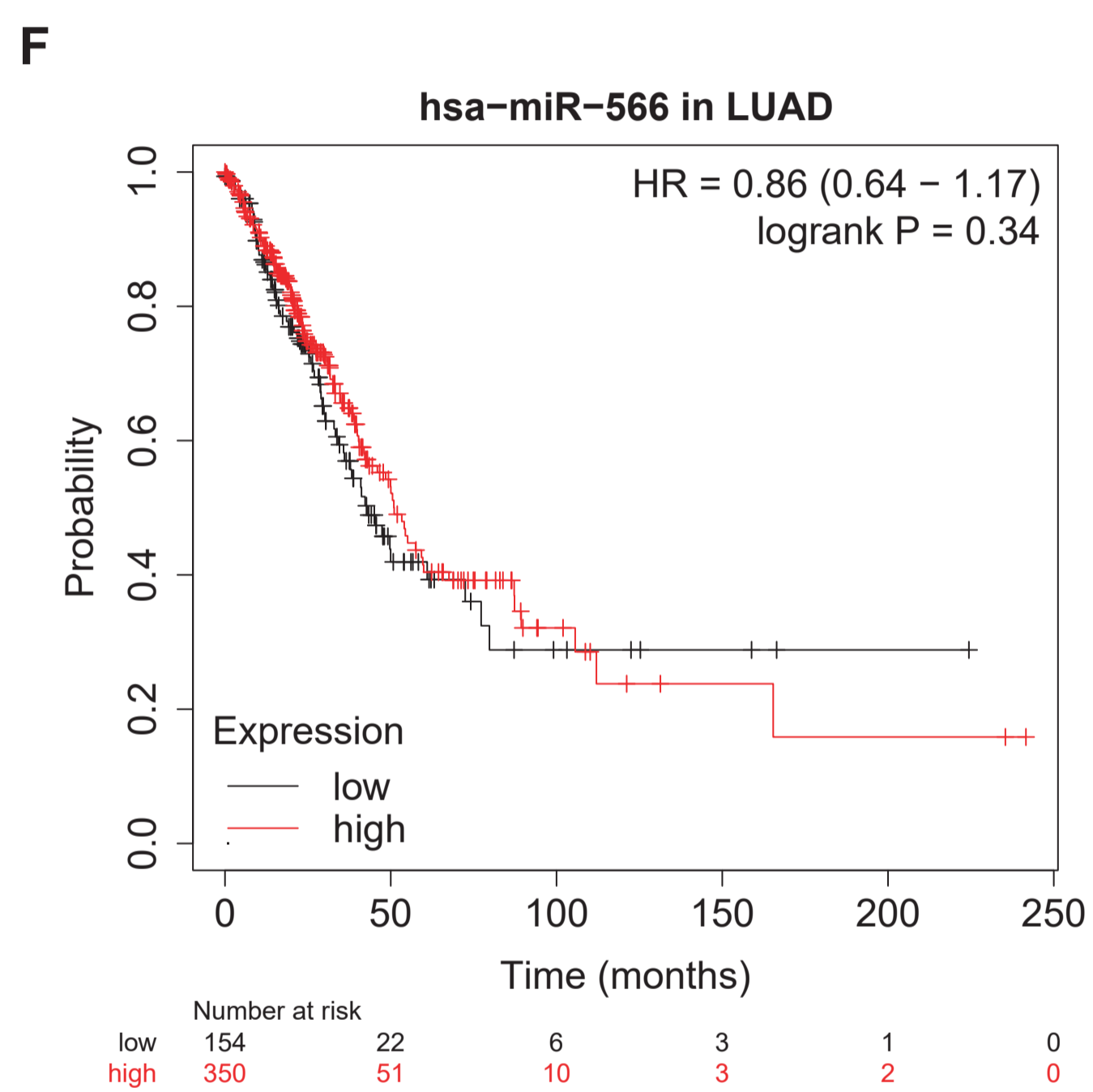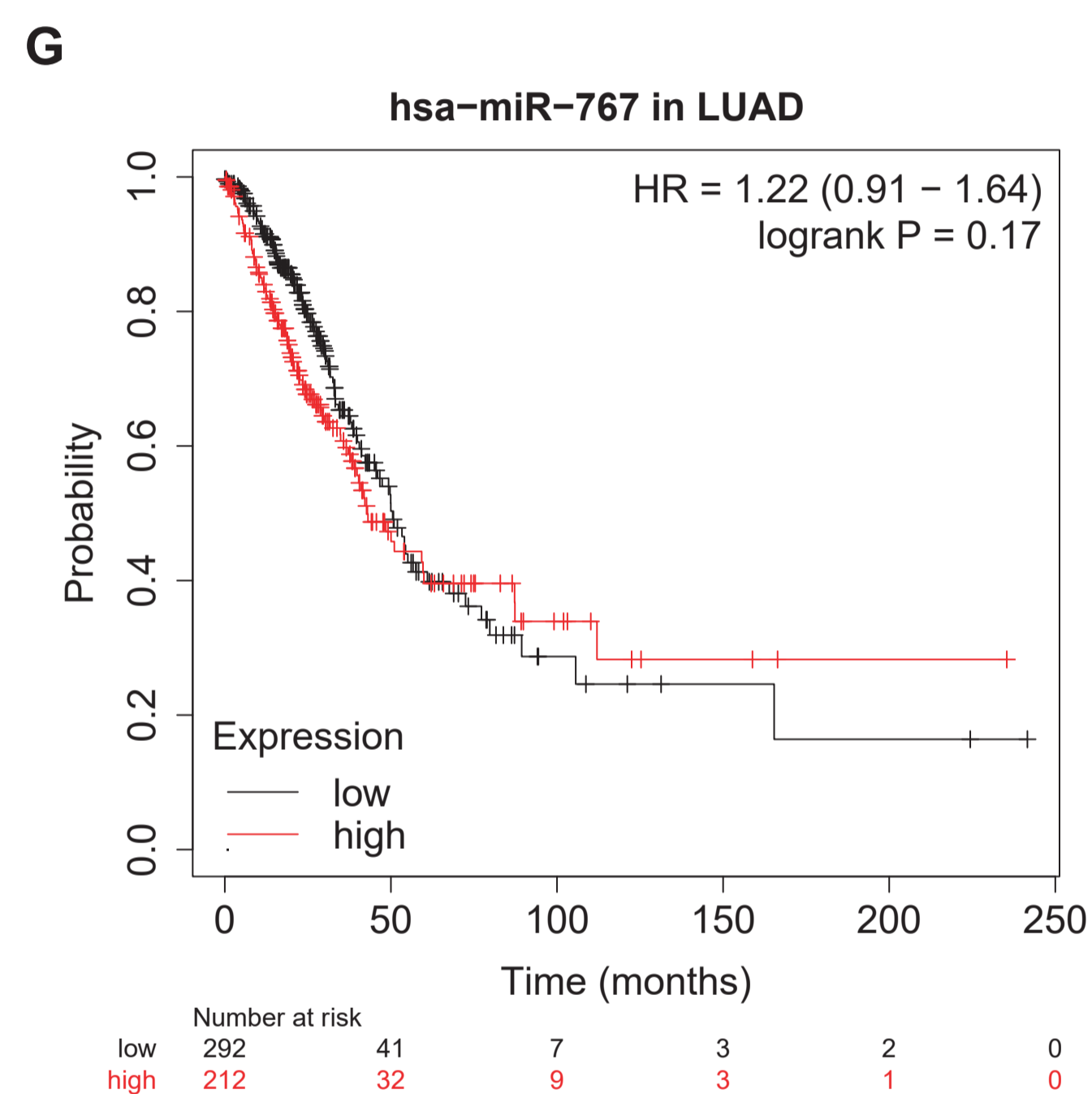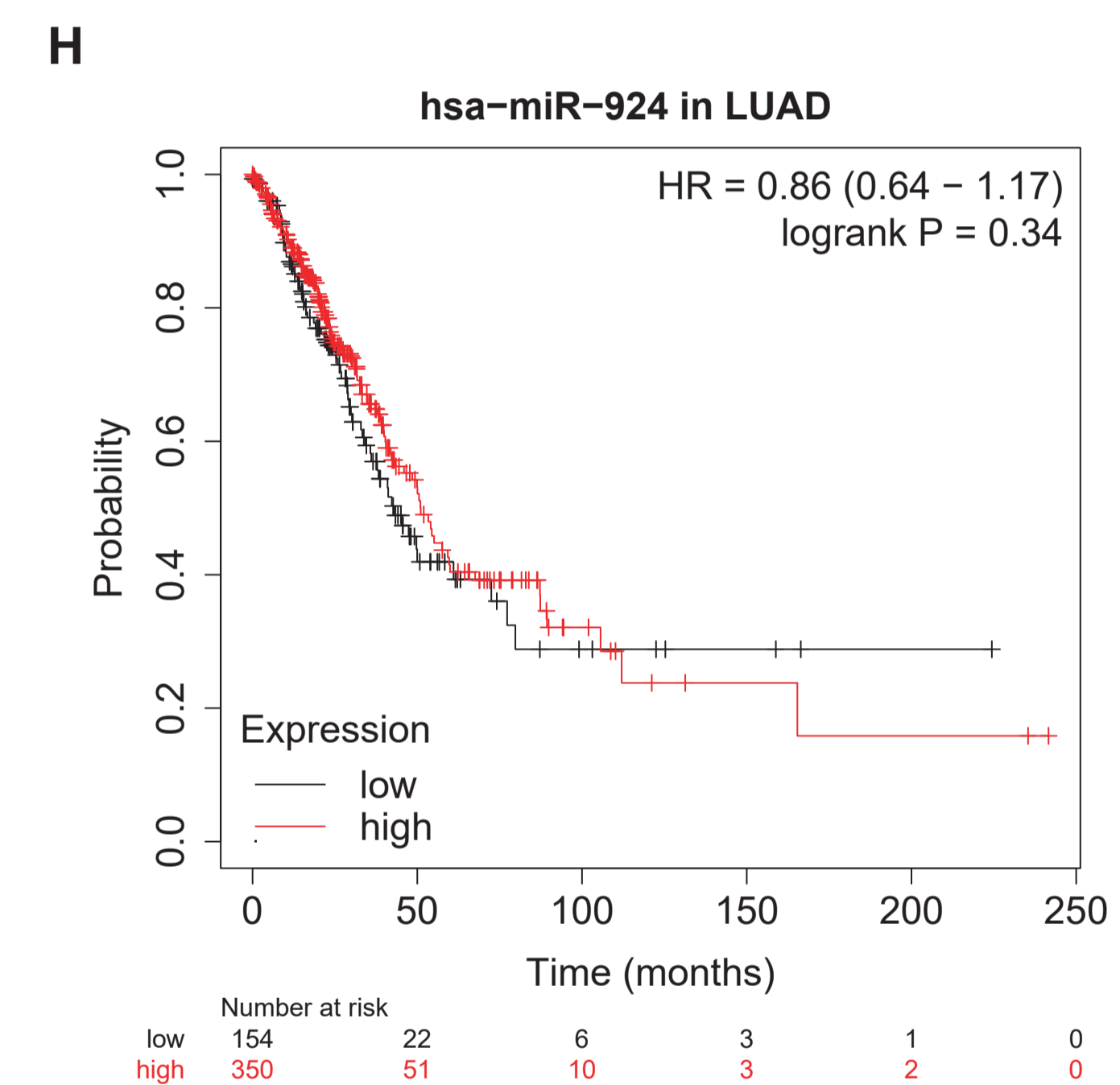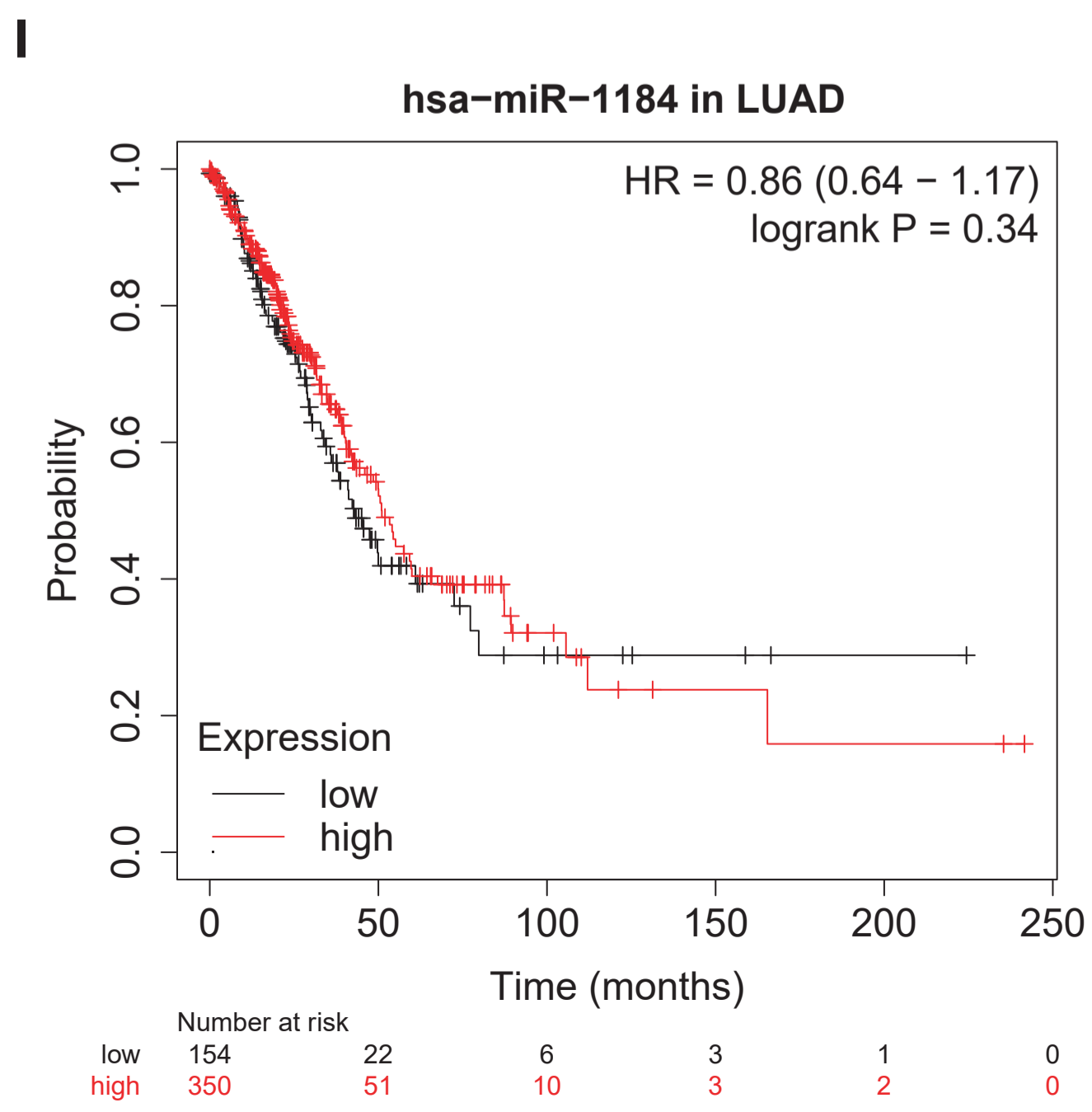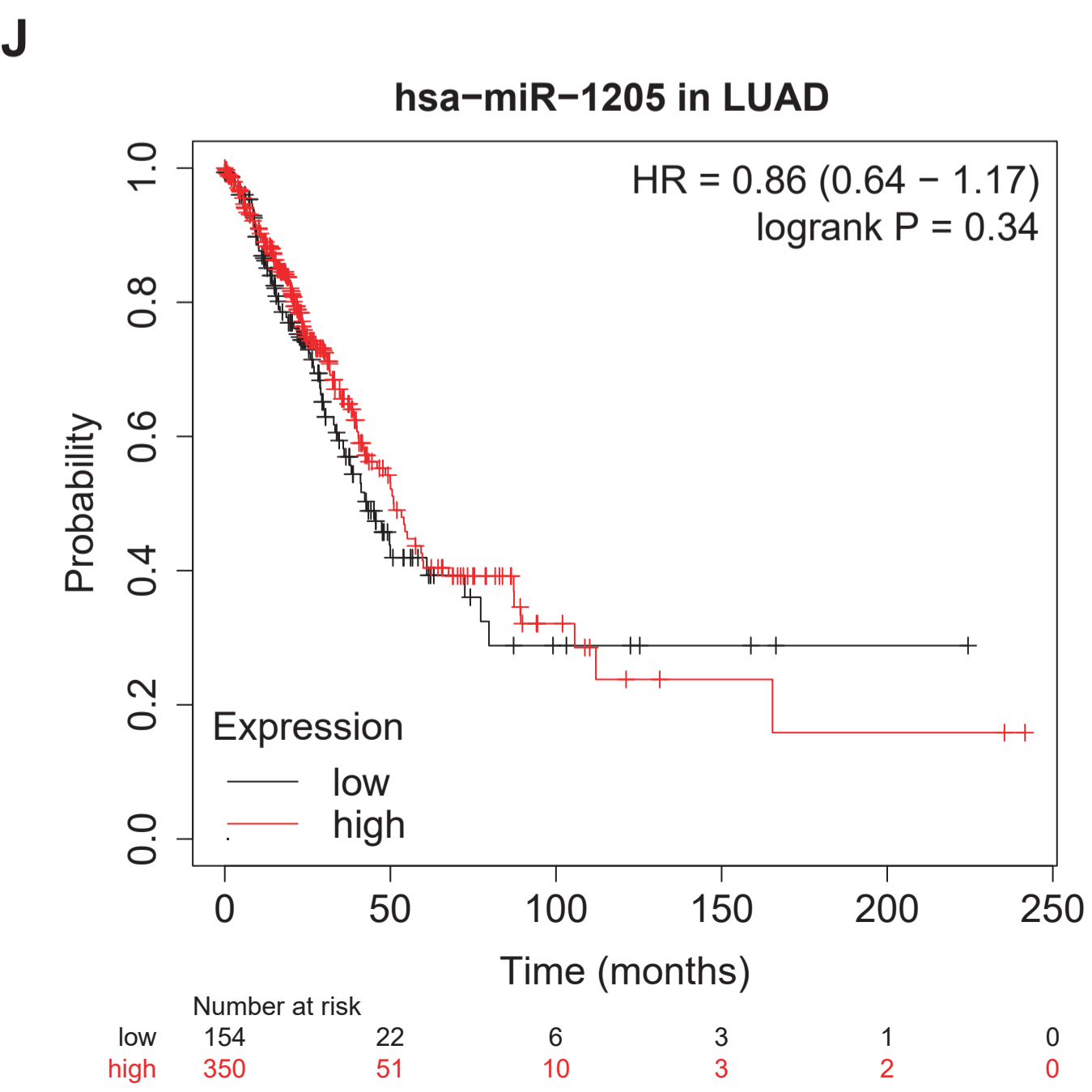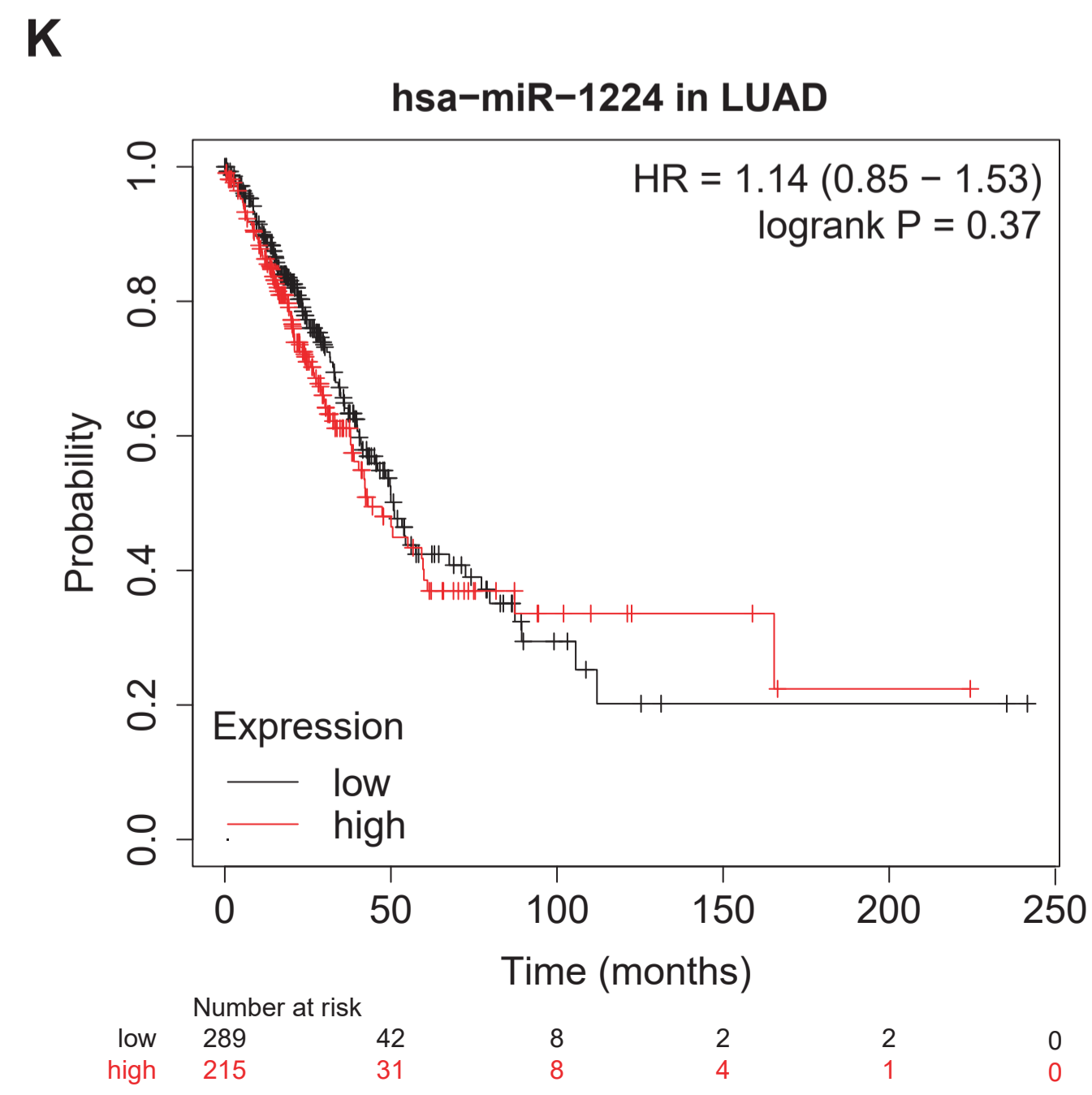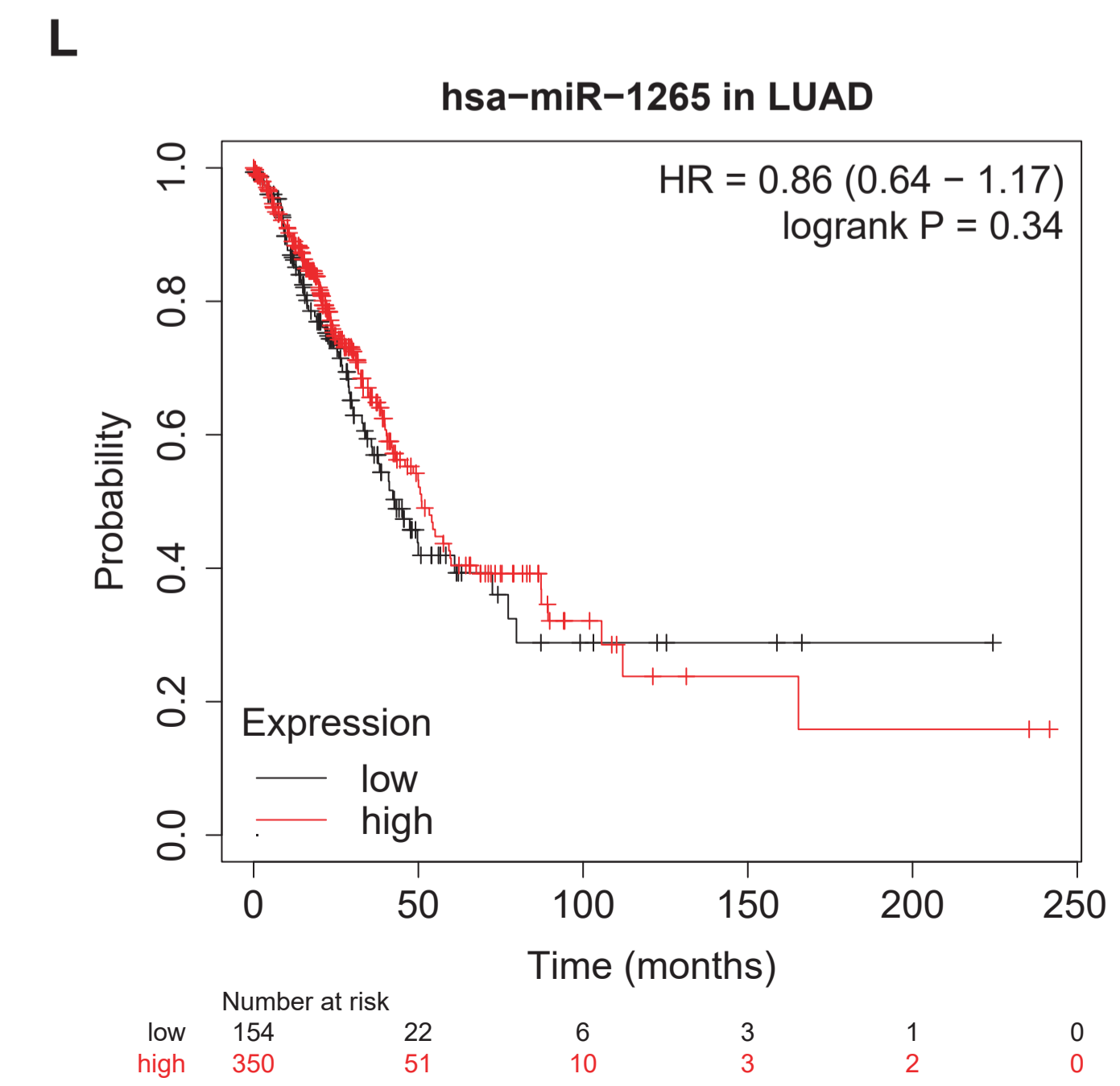

Supplement: Supplementary file 3 — Additional file 3:Fig. S3 The overall survival (OS) curves of miRNAs in LUAD. LUAD, lung adenocarcinoma. [file 12935_2021_2278_MOESM3_ESM.pdf]

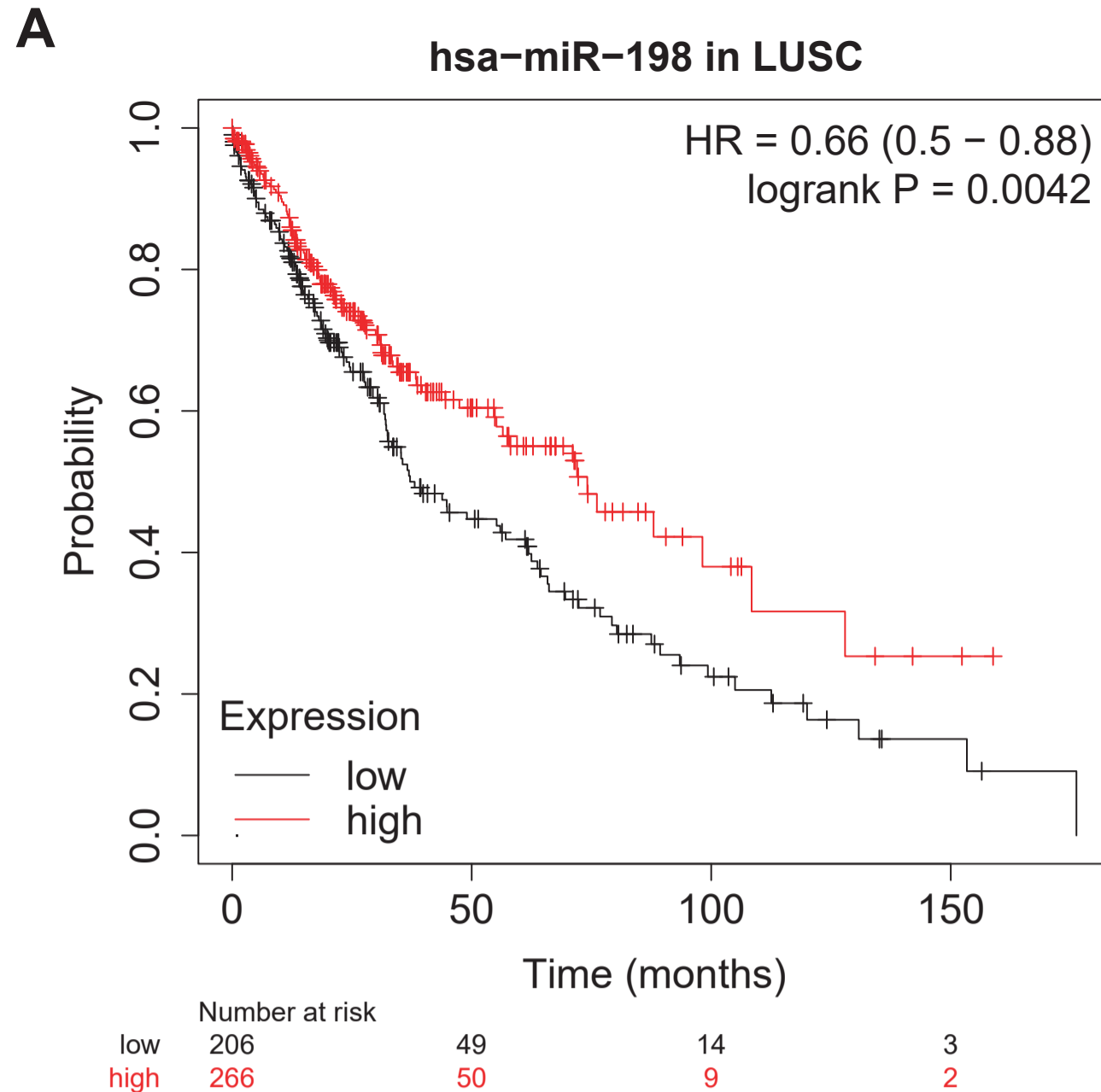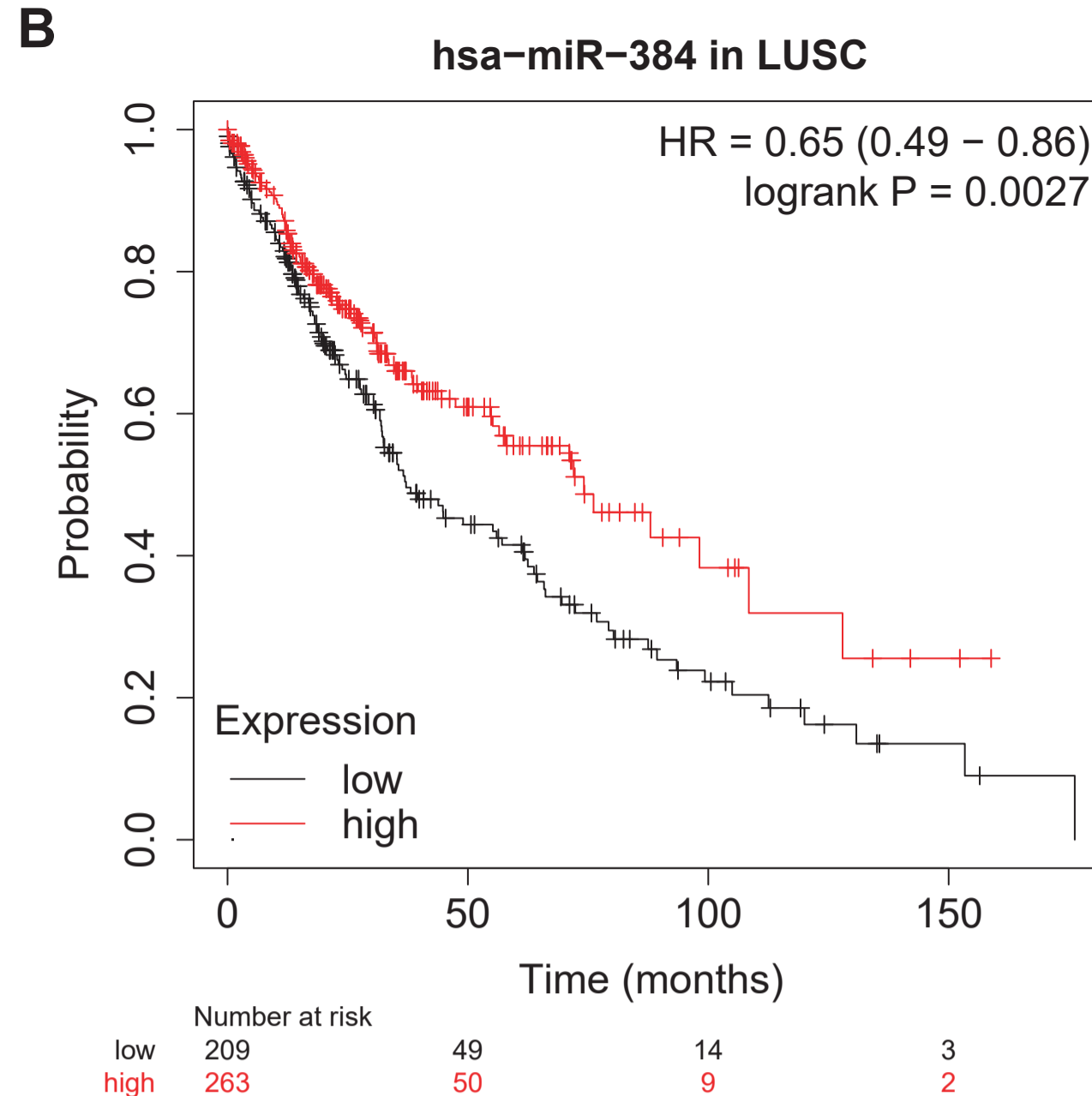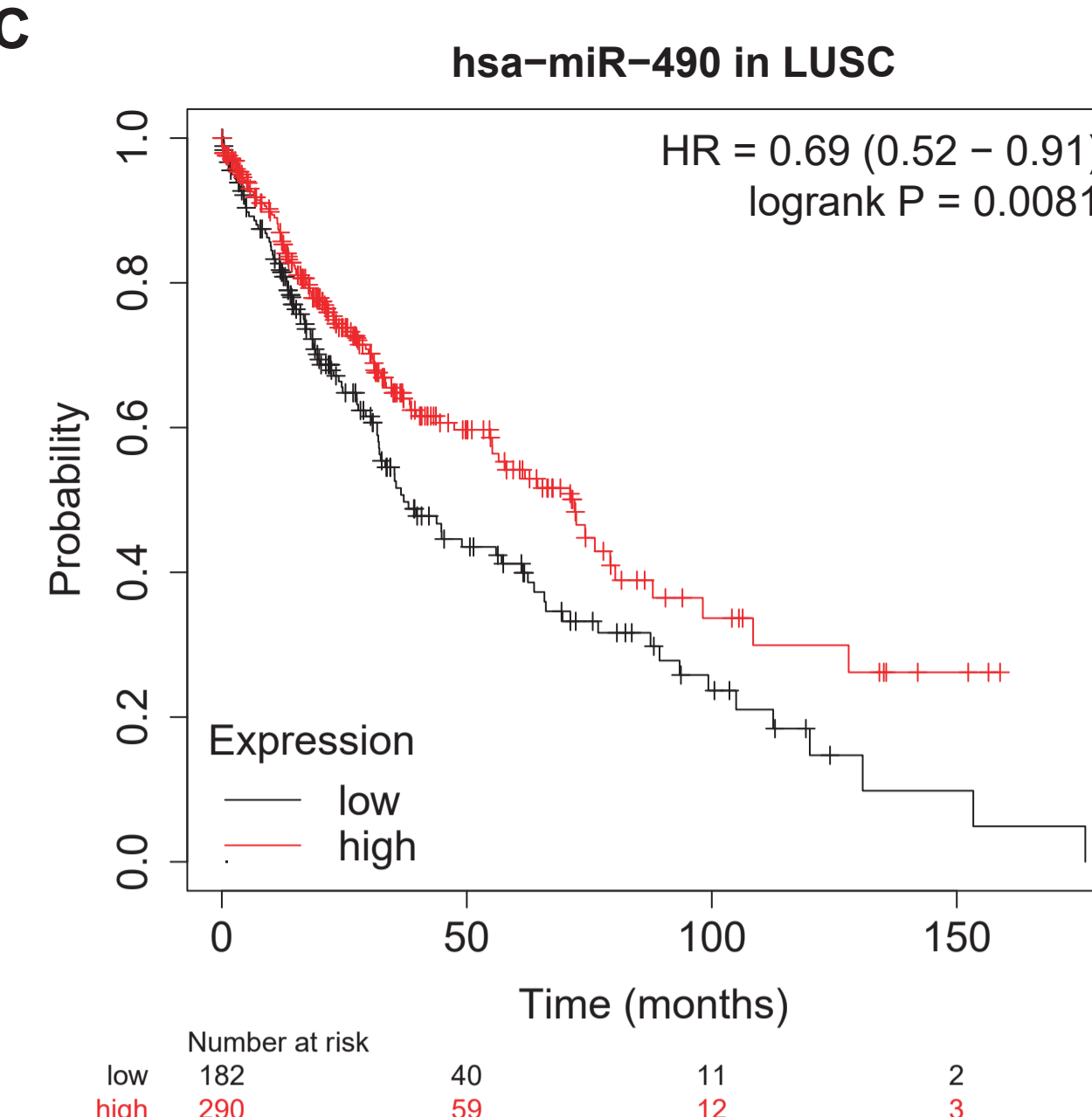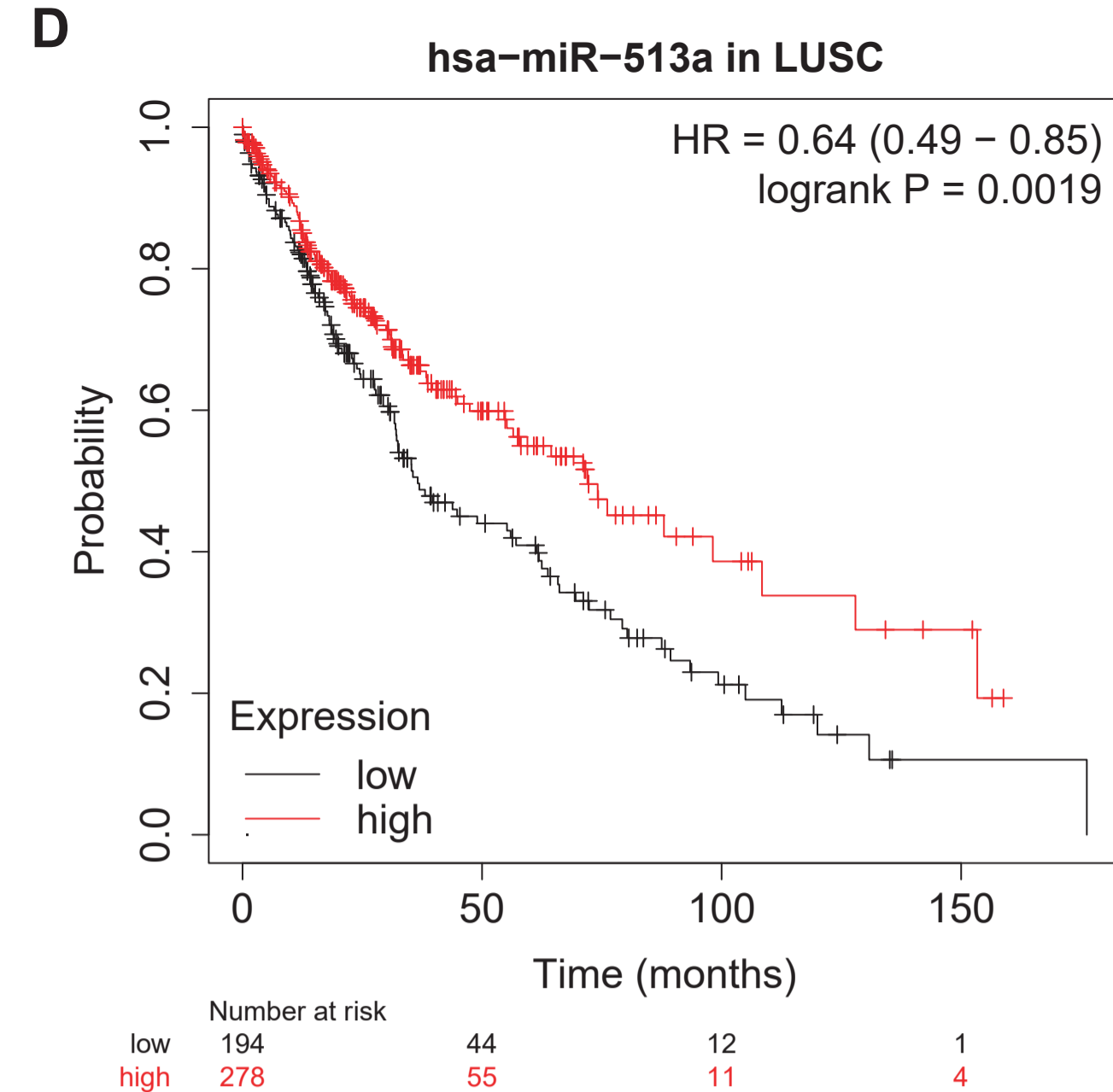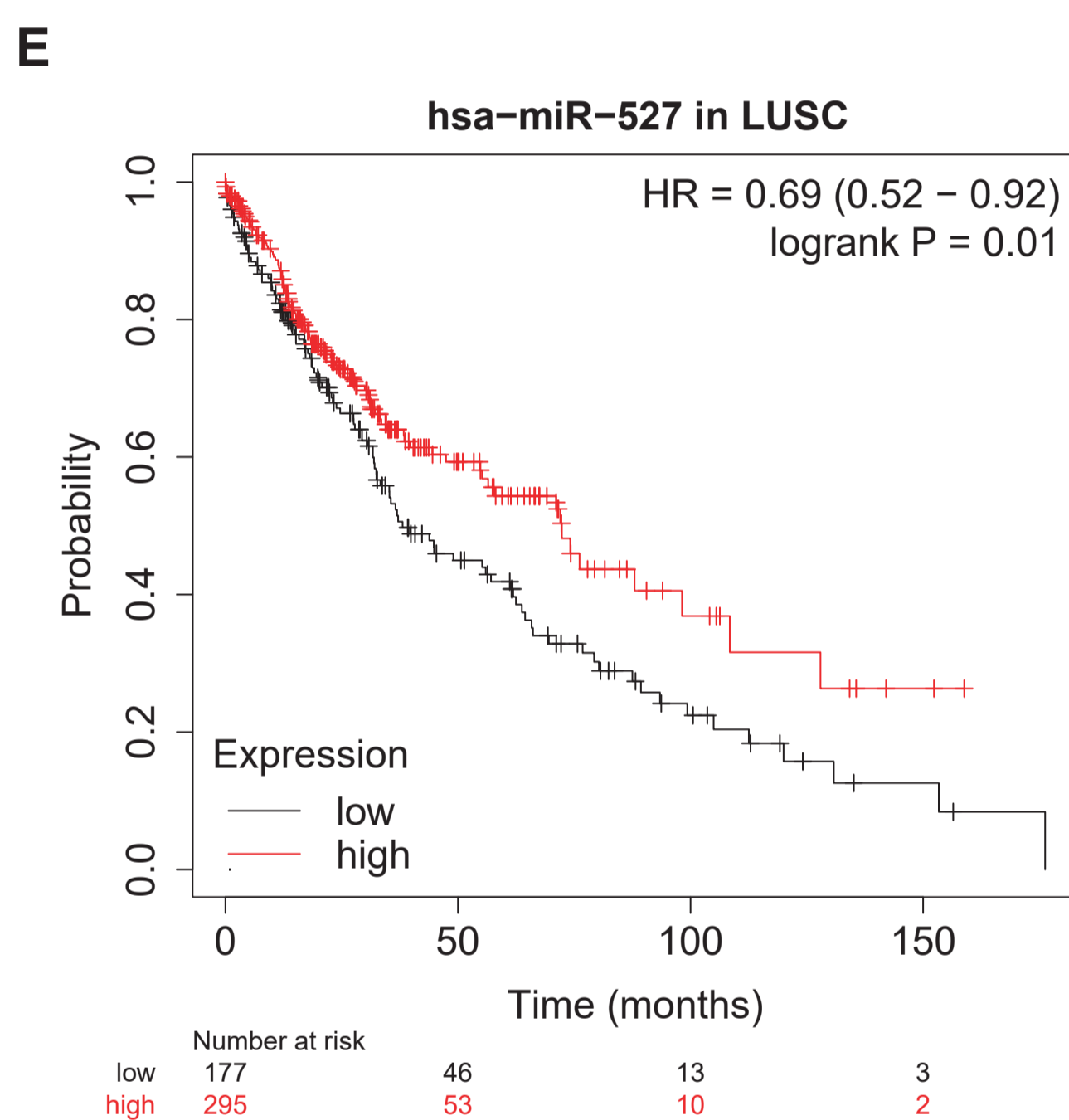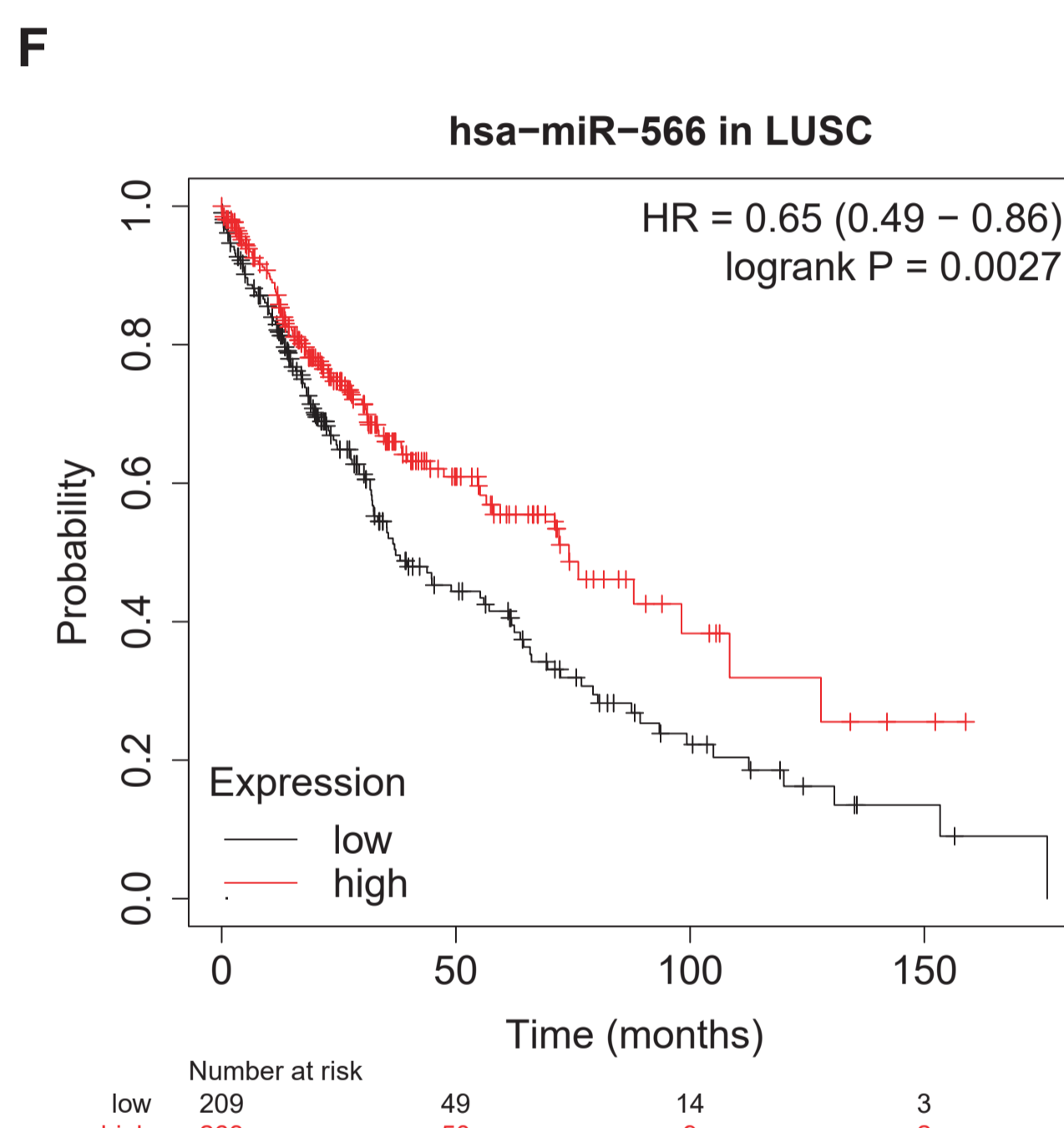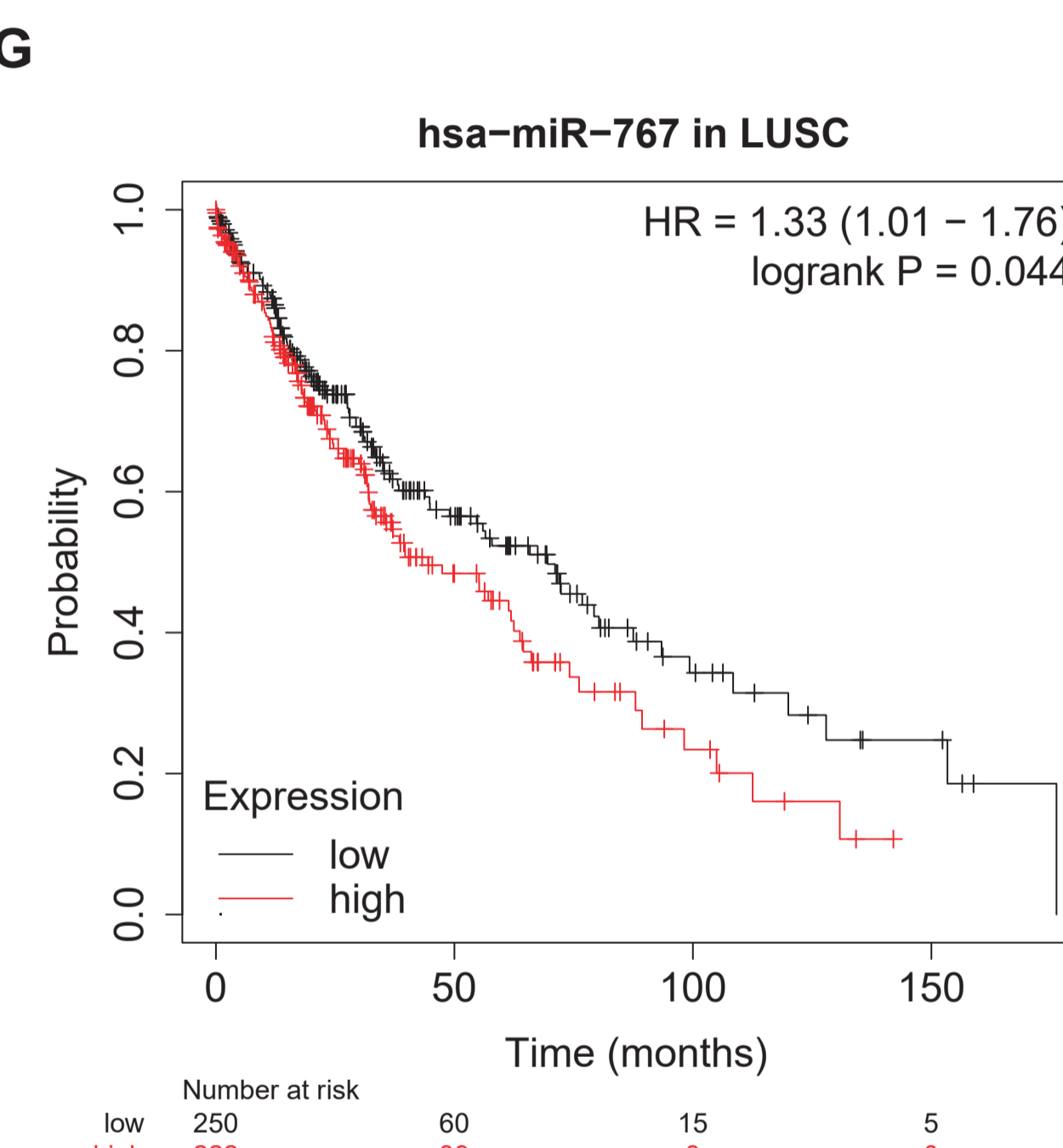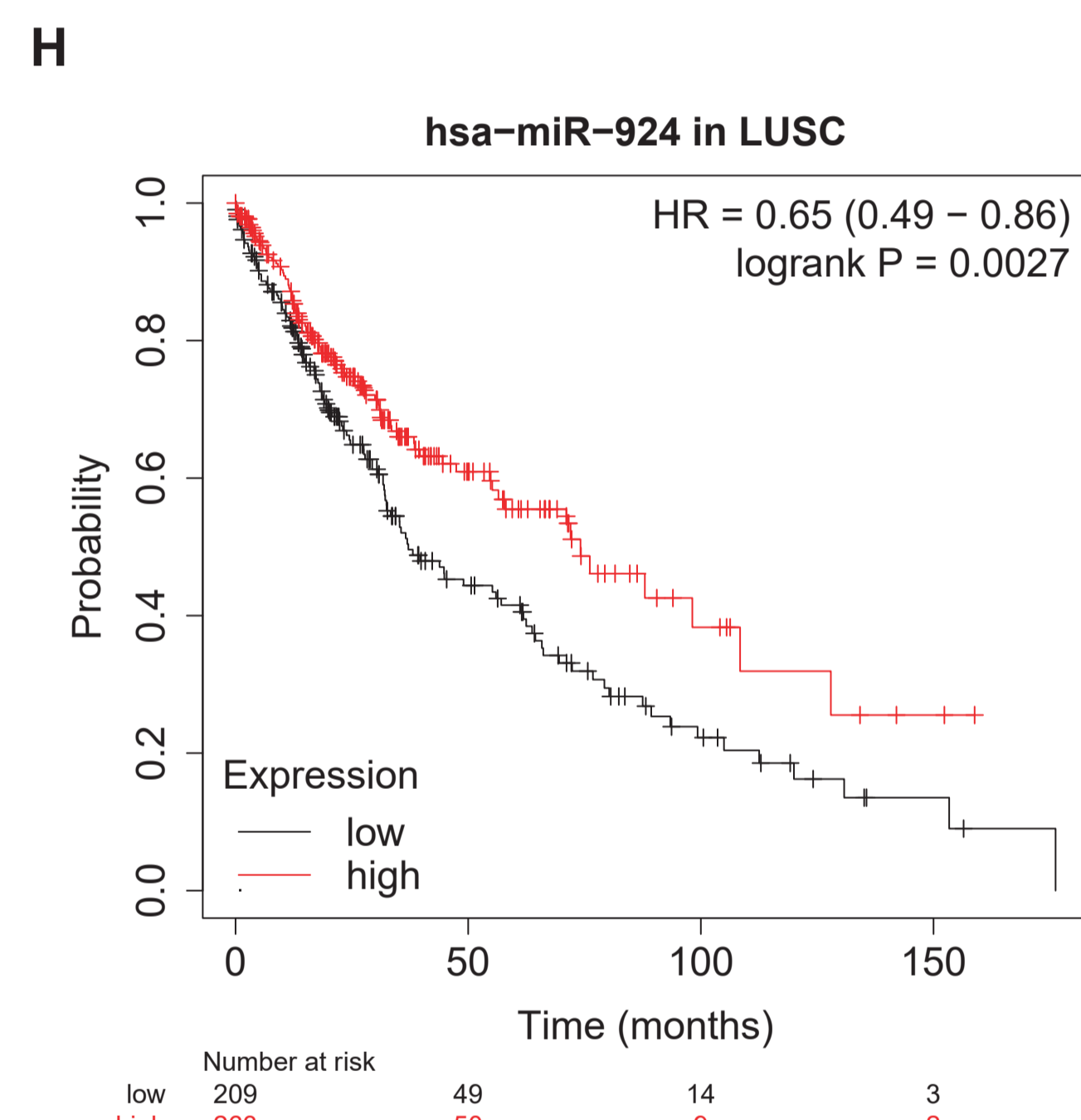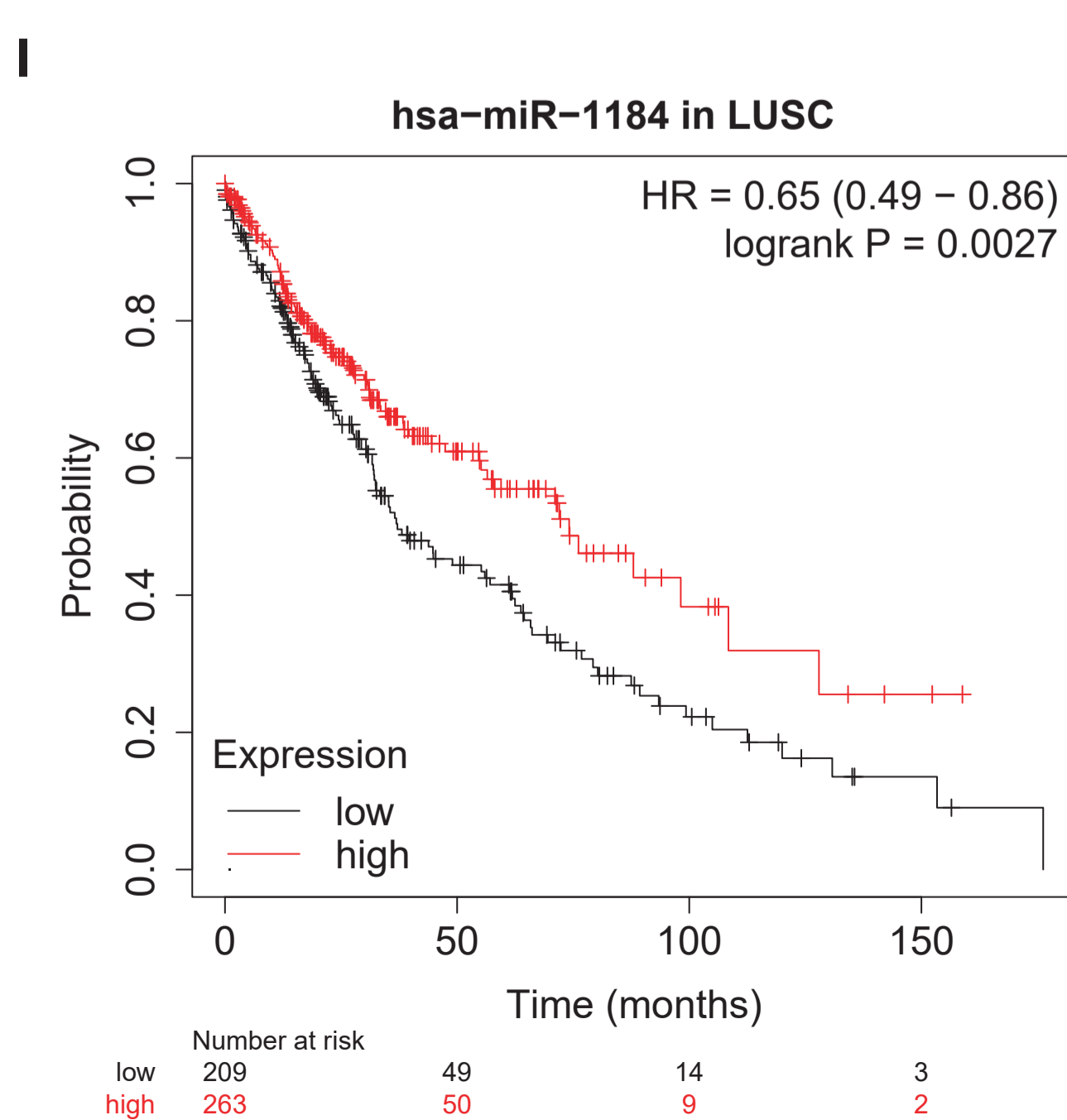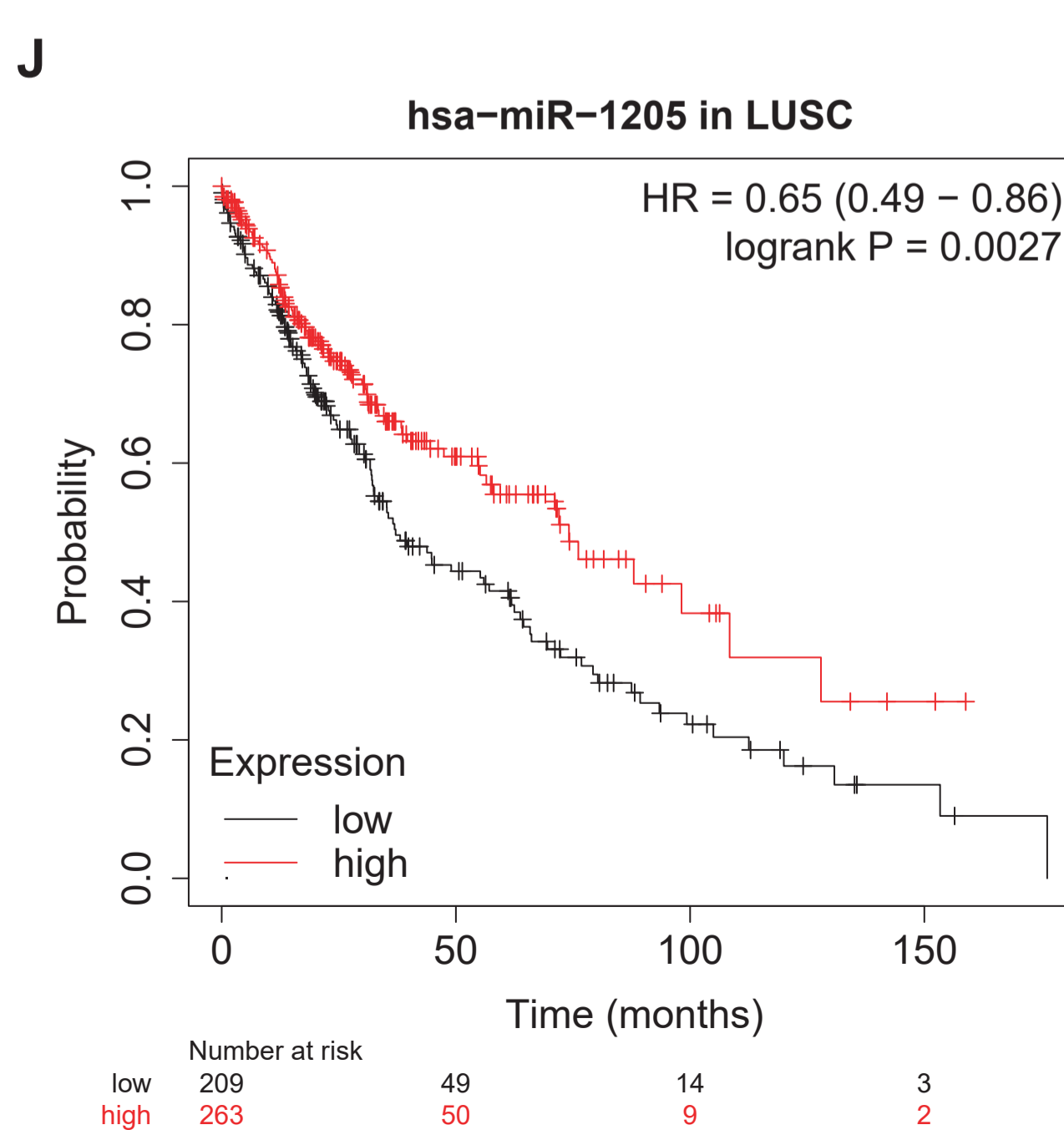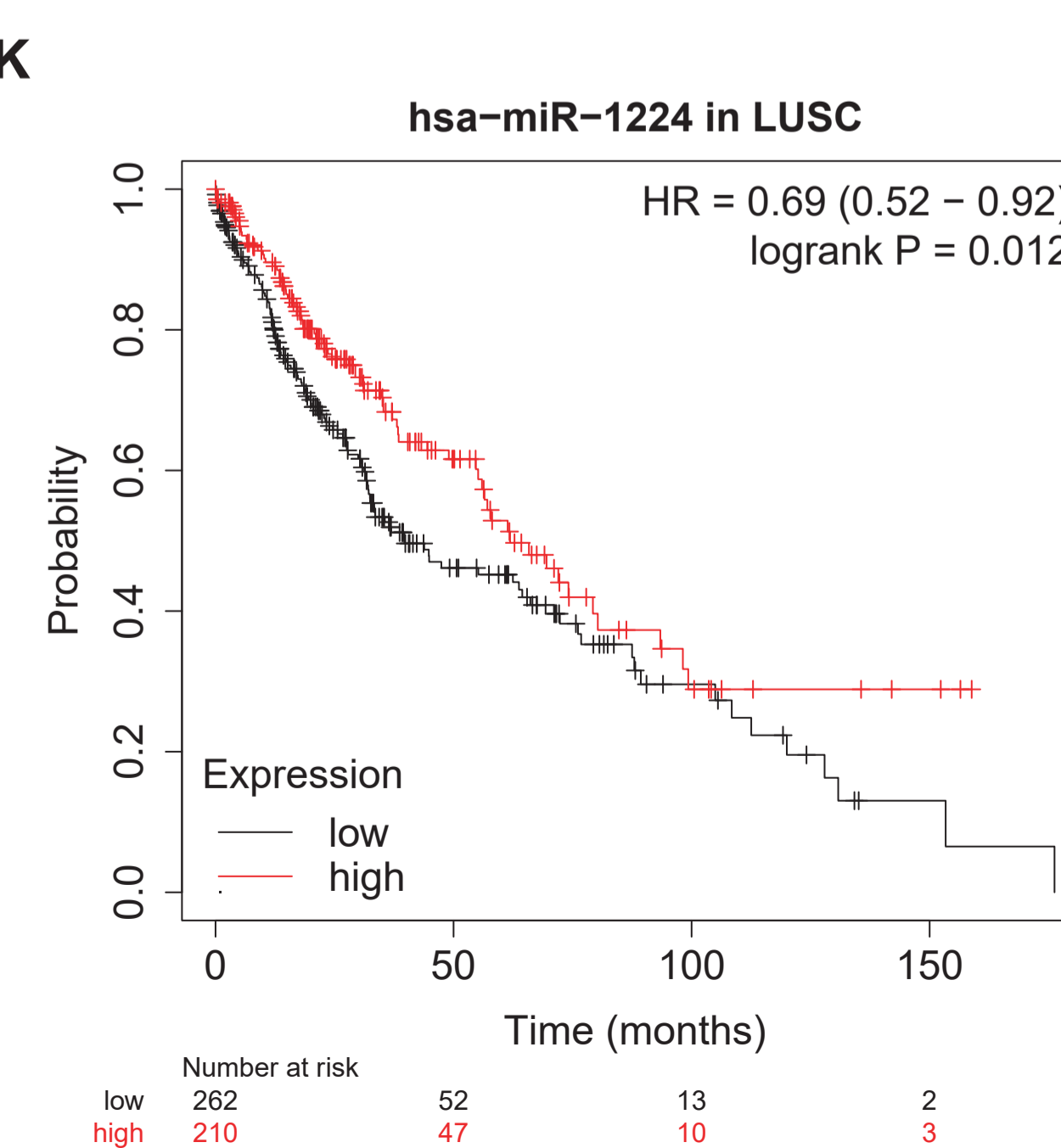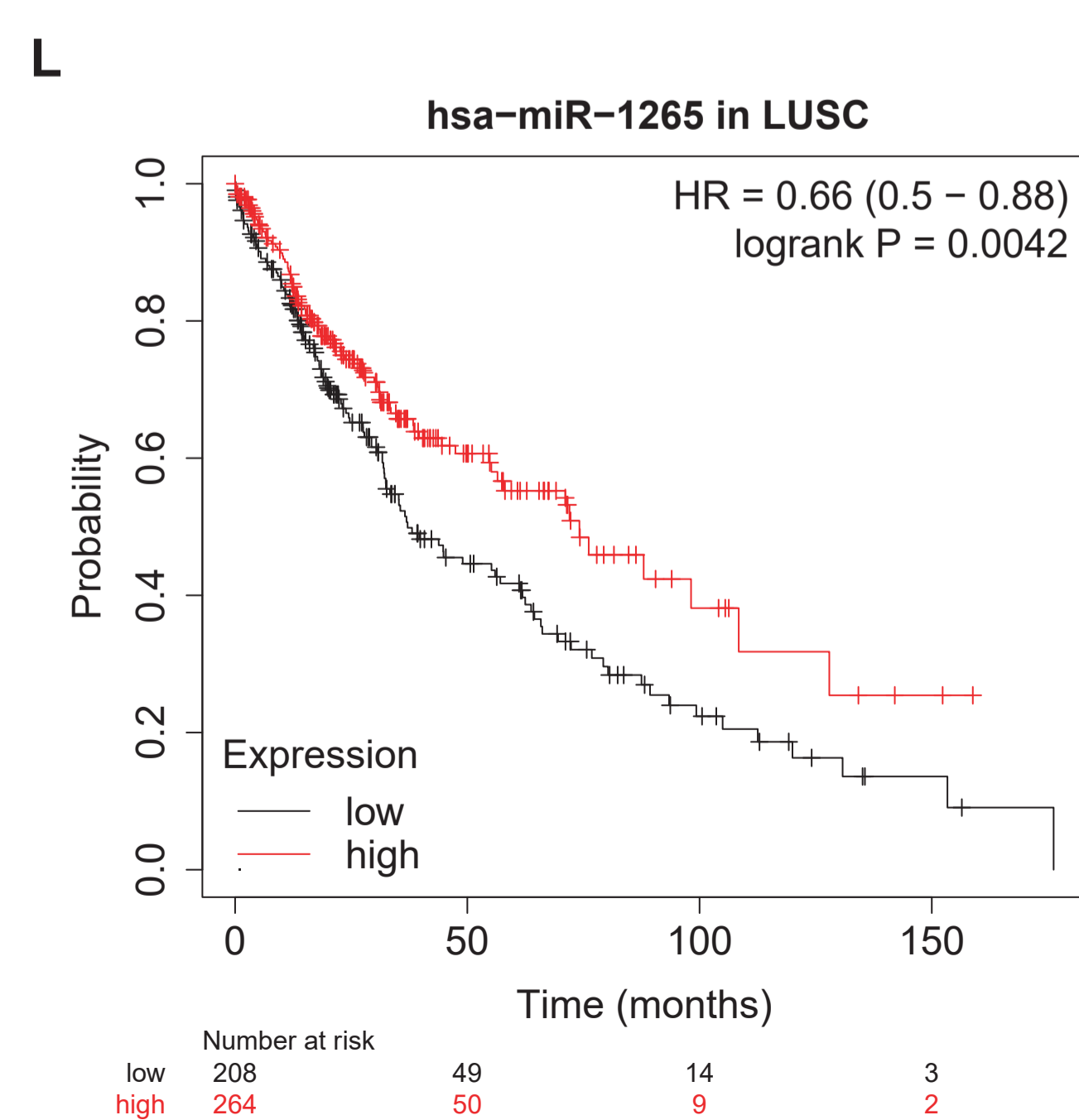

Supplement: Supplementary file 4 — Additional file 4: Fig. S4 The overall survival (OS) curves of miRNAs in LUSC. LUSC, lung squamous cell carcinoma. [file 12935_2021_2278_MOESM4_ESM.pdf]

# B

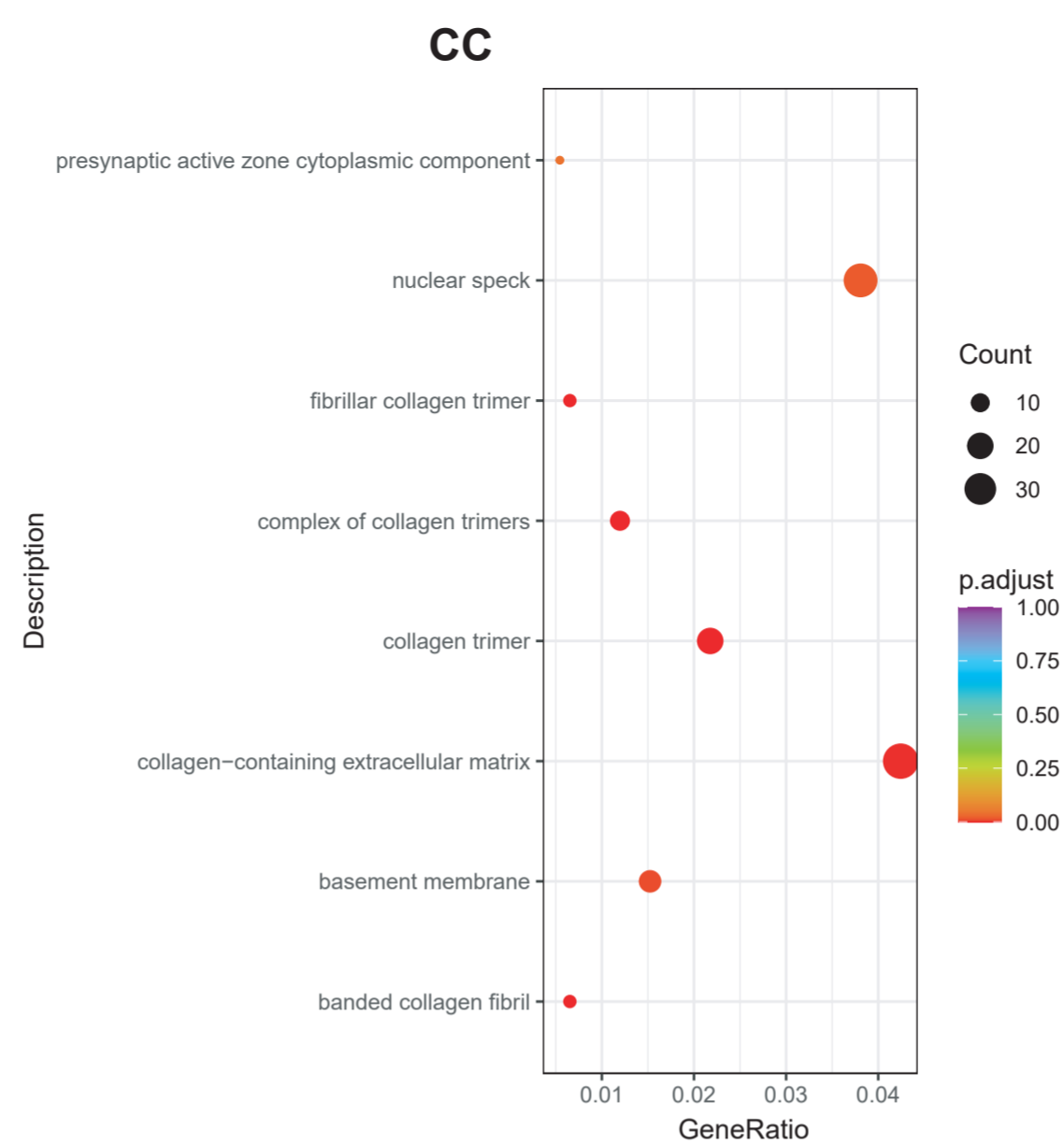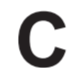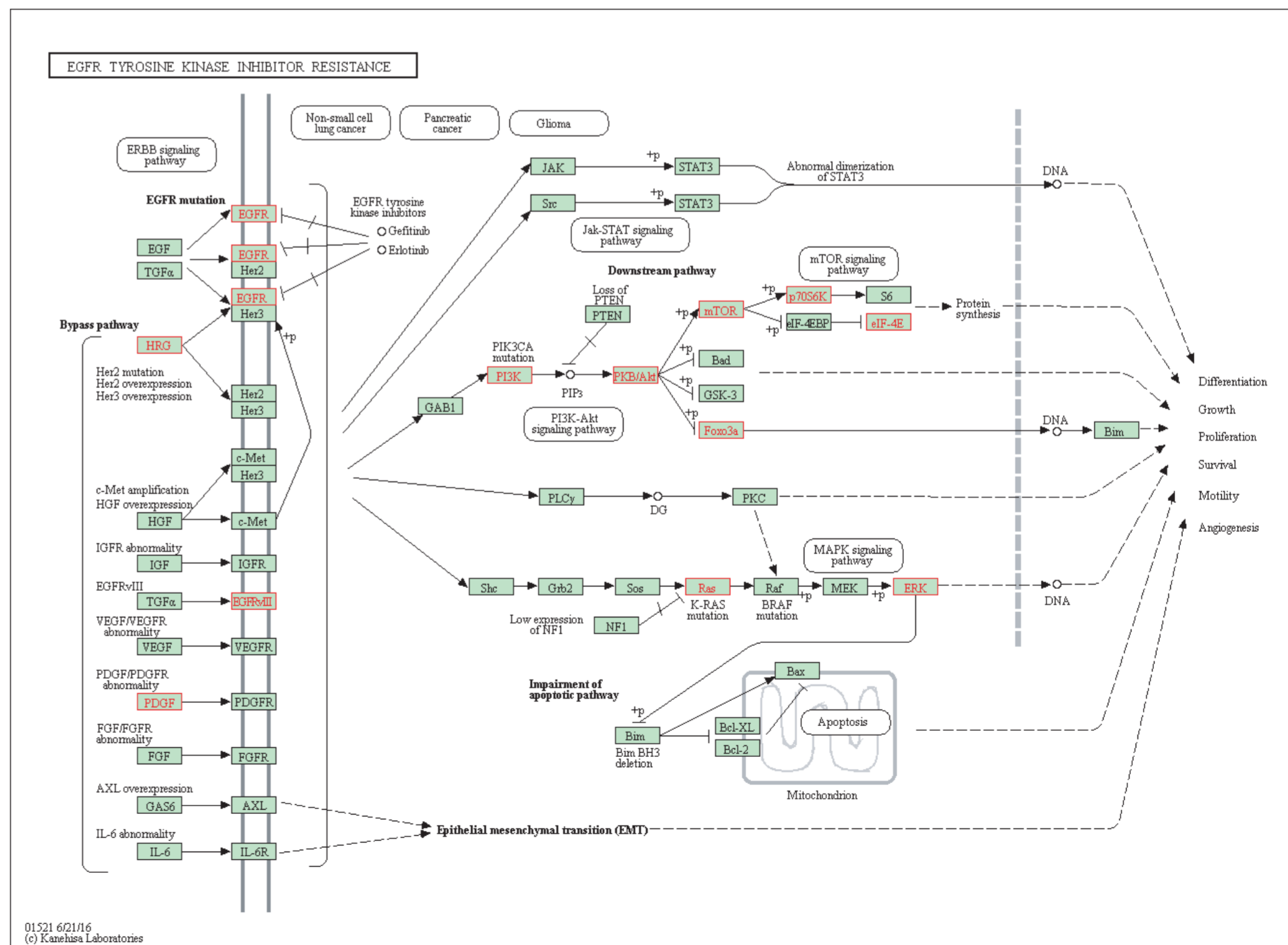

Supplement: Supplementary file 5 — Additional file 5: Fig. S5 Funrichment analysis of top 100 hub-genes in NSCLC. GO (a) and KEGG (b) pathway were performed to explore the potential biological mechanisms, and EGFR signaling pathway was associated with hub-genes (c). NSCLC, non-small cell lung cancer; GO, gene ontology; KEGG, kyoto encyclopedia of genes and genomes. [file 12935_2021_2278_MOESM5_ESM.pdf]

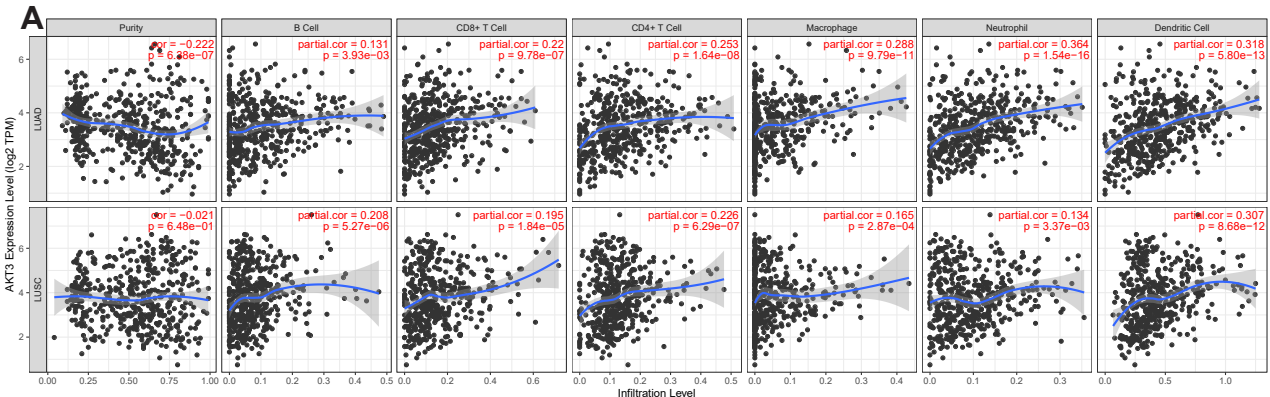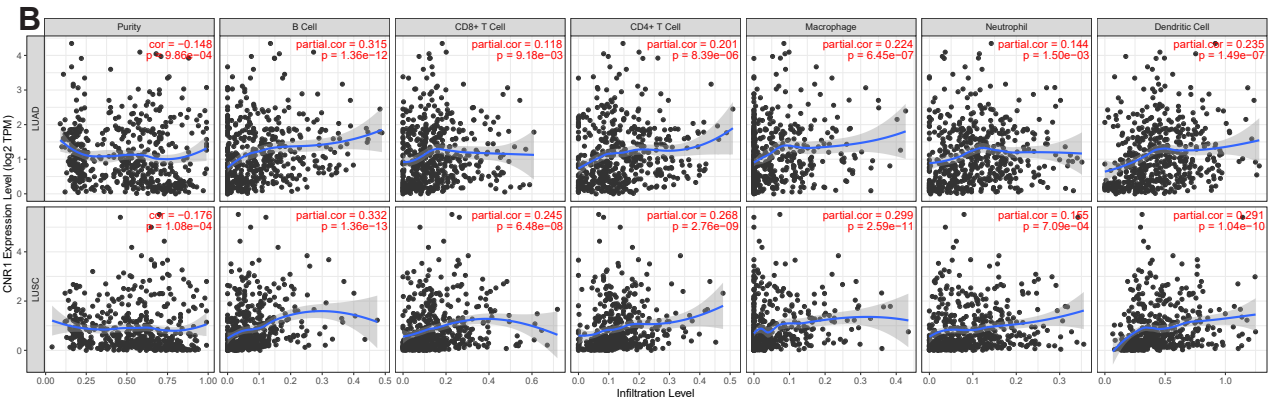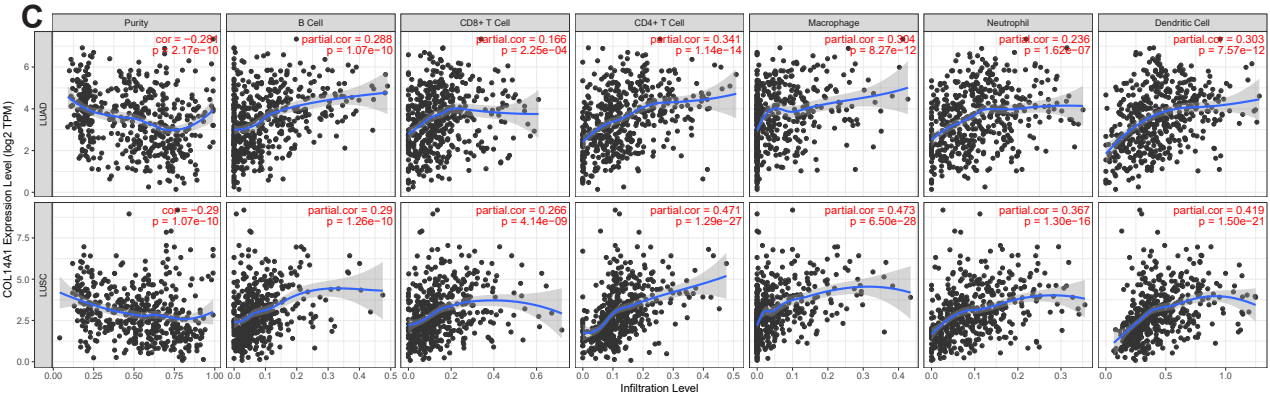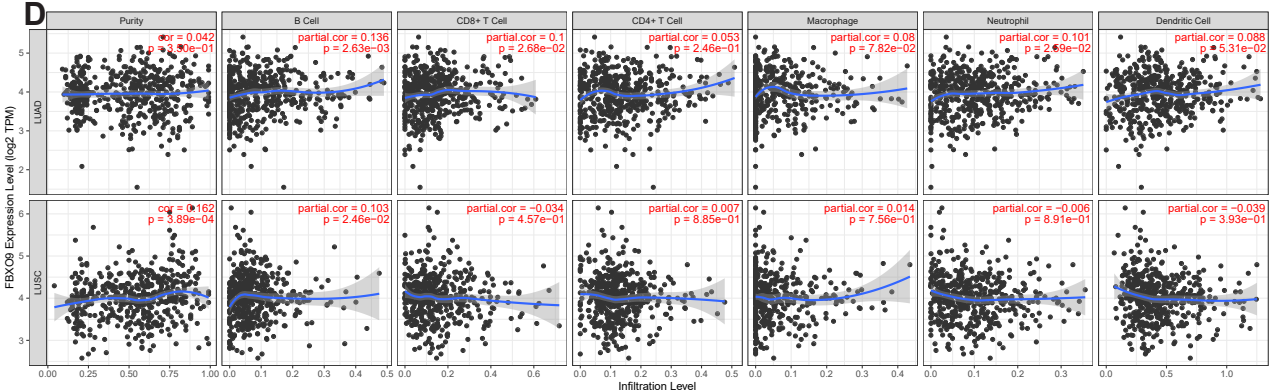

Supplement: Supplementary file 6 — Additional file 6: Fig. S6 Correlation between miRNA-target genes and immune infiltration in NSCLC. The correlation between AKT3 (a), CNR1 (b), COL14A1 (c), FBXO9 (d) and immune infiltration in NSCLC was analyzed by using the TIMER database. NSCLC, non-small cell lung cancer. [file 12935_2021_2278_MOESM6_ESM.pdf]

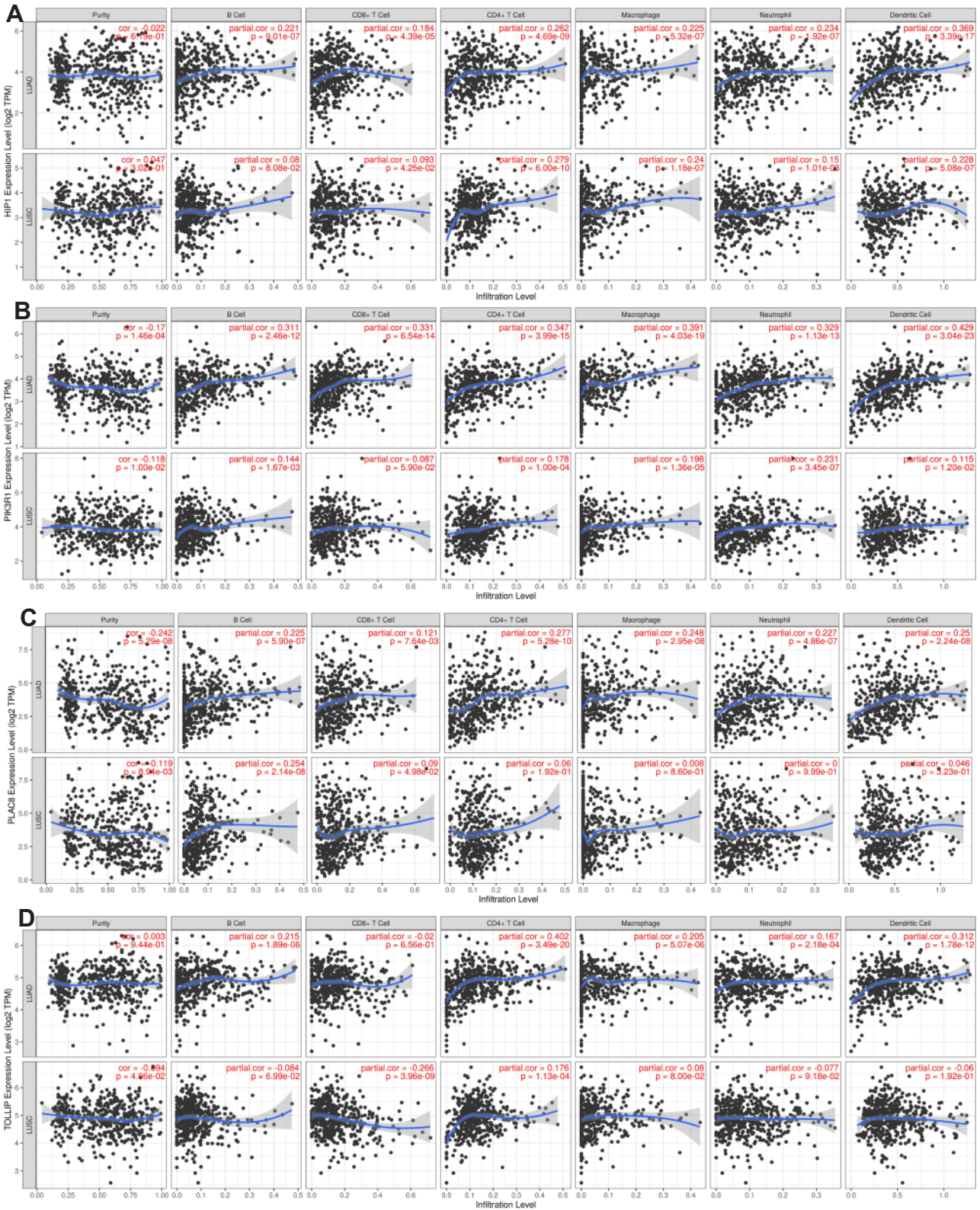

Supplement: Supplementary file 7 — Additional file 7: Fig. S7 Correlation between miRNA-target genes and immune infiltration in NSCLC. The correlation between HIP1 (a), PIK3R1 (b), PLAC8 (c), TOLLIP (d) and immune infiltration in NSCLC was analyzed by using the TIMER database. NSCLC, non-small cell lung cancer. [file 12935_2021_2278_MOESM7_ESM.pdf]

A

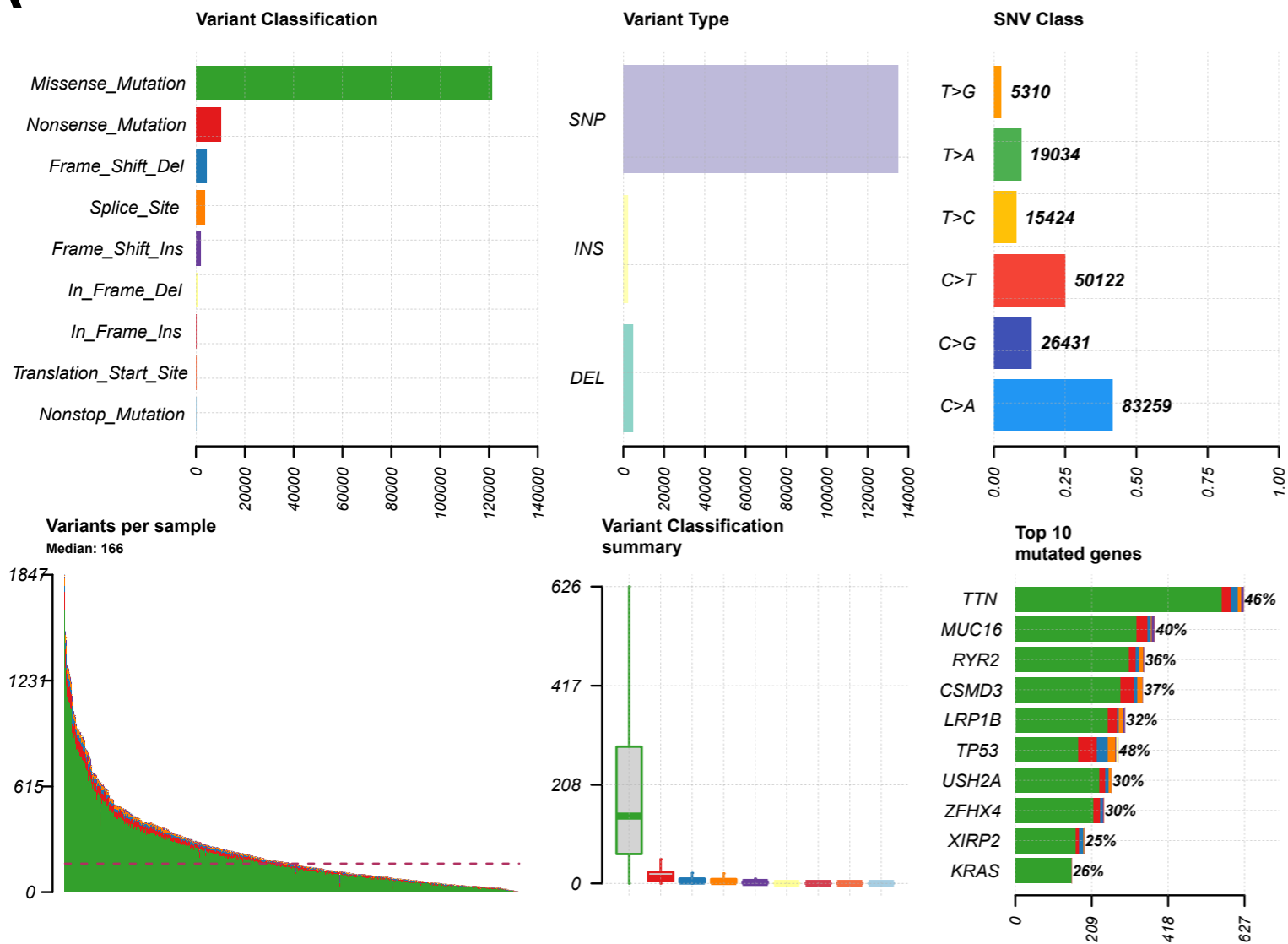

B

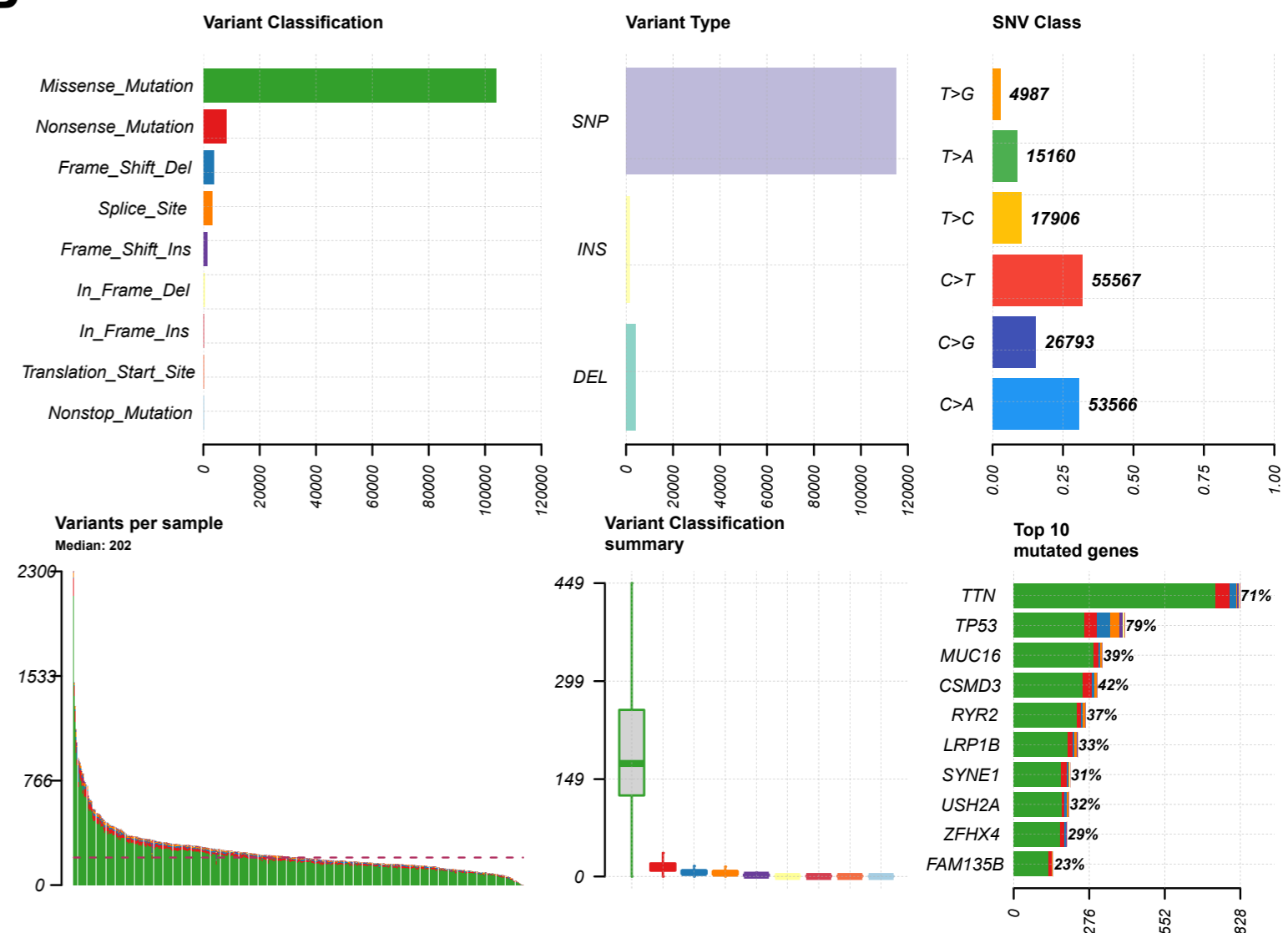

C

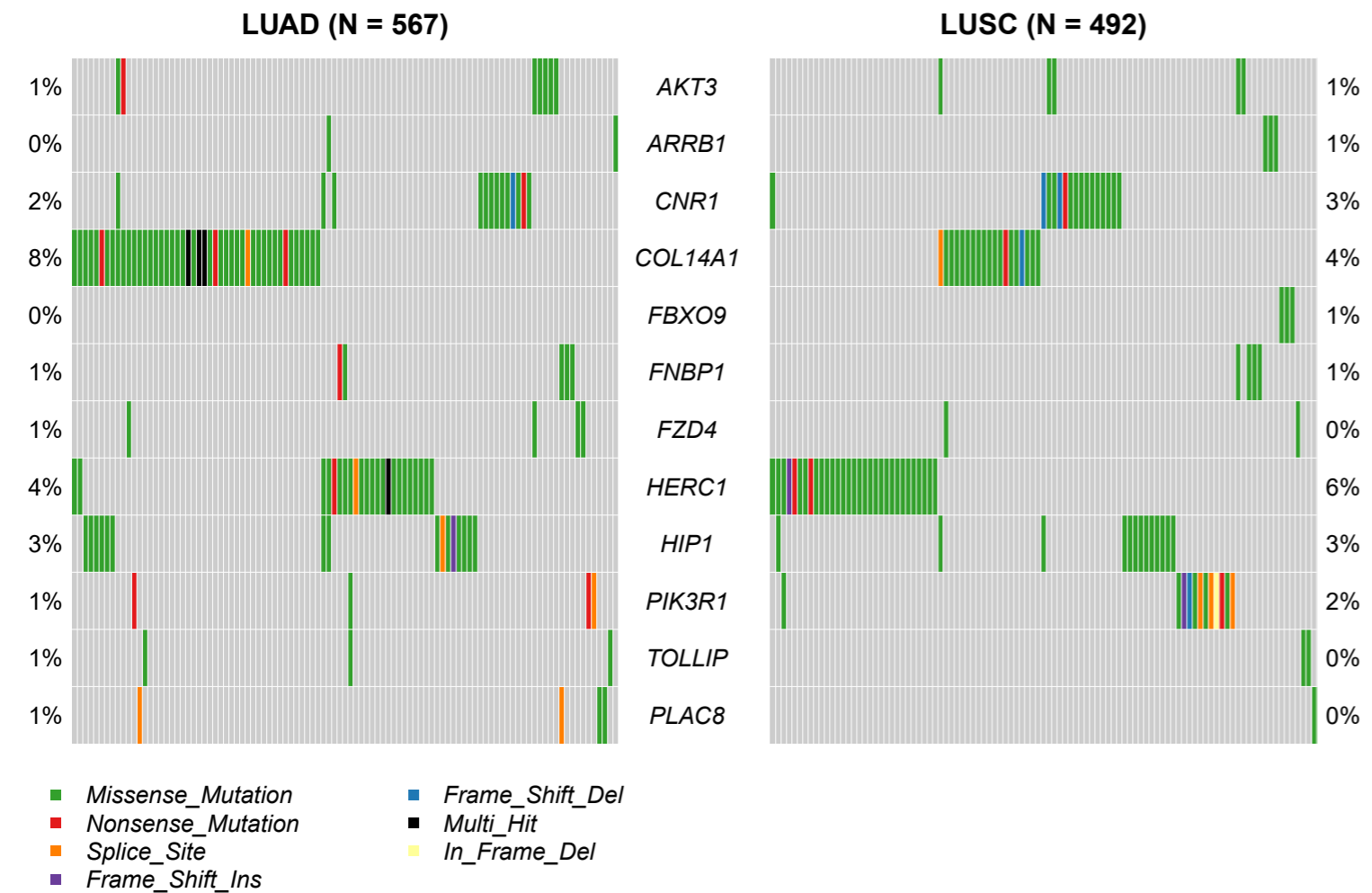

Supplement: Supplementary file 8 — Additional file 8: Fig. S8 Frequencies of mutations in miRNA-target gene. l. The overall situation of mutations in LUAD patients in TCGA. b. The overall situation of mutations in LUSC patients in TCGA. C. The mutation frequency of 12 target genes in NSCLC. NSCLC, non-small cell lung cancer; LUAD, lung adenocarcinoma; LUSC, lung squamous cell carcinoma. [file 12935_2021_2278_MOESM8_ESM.pdf]

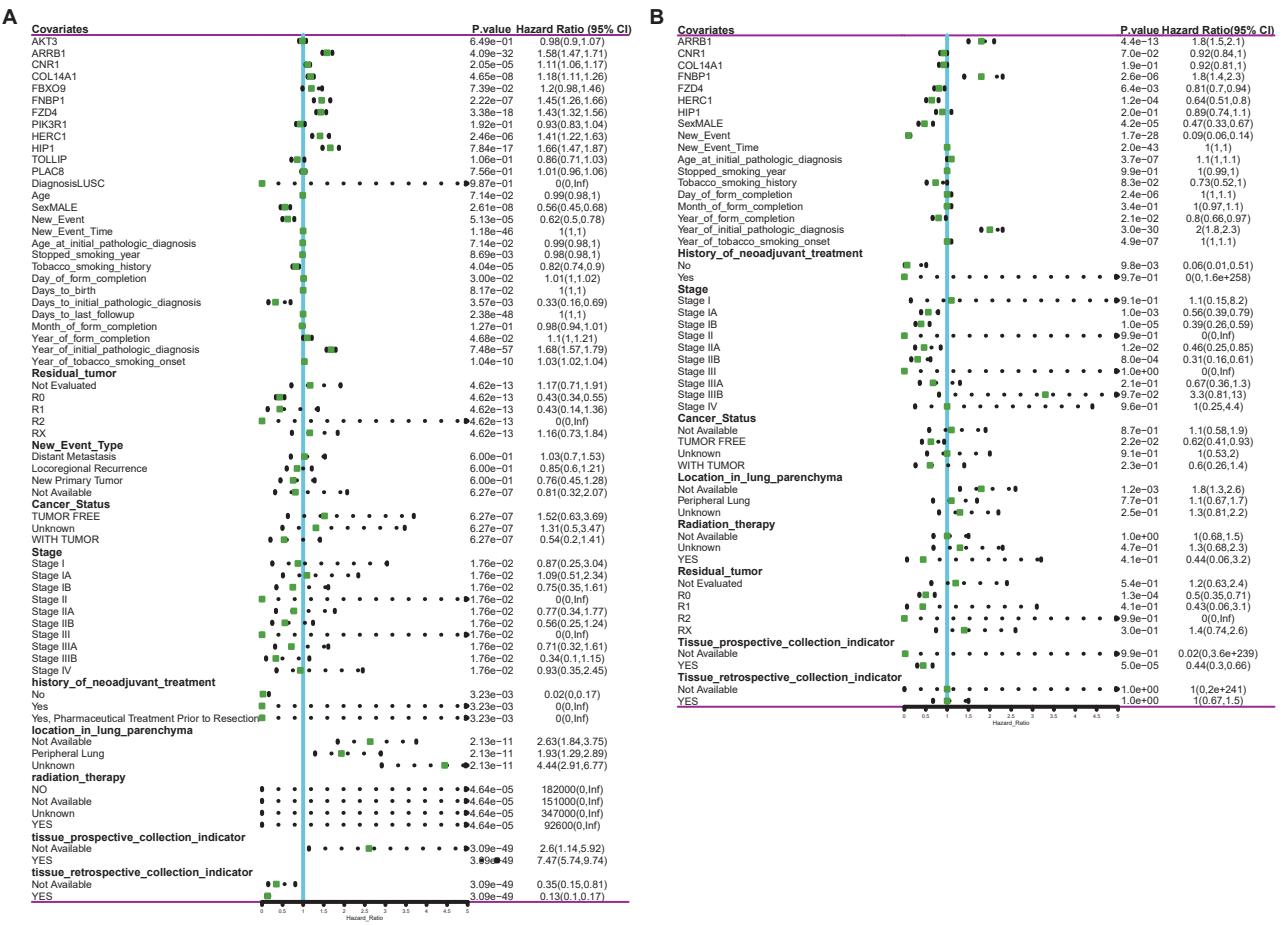

Supplement: Supplementary file 9 — Additional file 9: Fig. S9 Cox regression analyses of miRNA-target gene. The univariate COX regression (a) and multivariate COX regression (b) were used to evaluate the independent prognostic value of the twelve target genes in NSCLC patients with clinical features. NSCLC, non-small cell lung cancer. [file 12935_2021_2278_MOESM9_ESM.pdf]
