# Supplementary material for: Construction of circRNA-miRNA-mRNA network and identification of novel potential biomarkers for non-small cell lung cancer
Source: Cancer Cell Int. 2021 Nov 20;21:611. doi: 10.1186/s12935-021-02278-z (PMC8605517; doi:10.1186/s12935-021-02278-z)
Supplement: Supplementary file 10 — Additional file 10: Table S1. Results of GO enrichment. Table S2. Results of KEGG enrichment. [file 12935_2021_2278_MOESM10_ESM.docx]

**Table S1** Results of GO enrichment

| GO enrichment | ID | Description | *P*-value |
| --- | --- | --- | --- |
| BP | GO:0098742 | cell-cell adhesion via plasma-membrane adhesion molecules | 2.17E-06 |
| BP | GO:0007179 | transforming growth factor beta receptor signaling pathway | 2.47E-05 |
| BP | GO:0030199 | collagen fibril organization | 3.68E-05 |
| BP | GO:0016358 | dendrite development | 4.48E-05 |
| BP | GO:0007156 | homophilic cell adhesion via plasma membrane adhesion molecules | 4.59E-05 |
| BP | GO:0071560 | cellular response to transforming growth factor beta stimulus | 5.32E-05 |
| BP | GO:0021953 | central nervous system neuron differentiation | 5.57E-05 |
| BP | GO:0007178 | transmembrane receptor protein serine/threonine kinase signaling pathway | 5.62E-05 |
| BP | GO:0006024 | glycosaminoglycan biosynthetic process | 5.89E-05 |
| BP | GO:0071230 | cellular response to amino acid stimulus | 7.33E-05 |
| BP | GO:0098693 | regulation of synaptic vesicle cycle | 7.35E-05 |
| BP | GO:0050773 | regulation of dendrite development | 7.94E-05 |
| BP | GO:0071559 | response to transforming growth factor beta | 8.00E-05 |
| BP | GO:0006023 | aminoglycan biosynthetic process | 0.000101 |
| BP | GO:0021952 | central nervous system projection neuron axonogenesis | 0.000117 |
| BP | GO:0018209 | peptidyl-serine modification | 0.000146 |
| BP | GO:0030198 | extracellular matrix organization | 0.000158 |
| BP | GO:0043062 | extracellular structure organization | 0.000166 |
| BP | GO:0021955 | central nervous system neuron axonogenesis | 0.000183 |
| CC | GO:0098644 | complex of collagen trimers | 1.14E-10 |
| CC | GO:0005581 | collagen trimer | 2.34E-09 |
| CC | GO:0005583 | fibrillar collagen trimer | 3.81E-06 |
| CC | GO:0098643 | banded collagen fibril | 3.81E-06 |
| CC | GO:0062023 | collagen-containing extracellular matrix | 1.65E-05 |
| CC | GO:0005604 | basement membrane | 0.000125 |
| CC | GO:0016607 | nuclear speck | 0.00024 |
| CC | GO:0098831 | presynaptic active zone cytoplasmic component | 0.000618 |
| MF | GO:0030020 | extracellular matrix structural constituent conferring tensile strength | 3.29E-11 |
| MF | GO:0005201 | extracellular matrix structural constituent | 4.02E-07 |
| MF | GO:0070851 | growth factor receptor binding | 9.99E-05 |
| MF | GO:0048407 | platelet-derived growth factor binding | 0.000102 |
| MF | GO:0046332 | SMAD binding | 0.000133 |

**Table S2** Results of KEGG enrichment

| ID | Description | *P*-value |
| --- | --- | --- |
| hsa04974 | Protein digestion and absorption | 1.40E-06 |
| hsa05231 | Choline metabolism in cancer | 2.82E-06 |
| hsa05210 | Colorectal cancer | 3.62E-05 |
| hsa04926 | Relaxin signaling pathway | 4.10E-05 |
| hsa04917 | Prolactin signaling pathway | 6.07E-05 |
| hsa04012 | ErbB signaling pathway | 0.000125 |
| hsa04933 | AGE-RAGE signaling pathway in diabetic complications | 0.000214 |
| hsa05224 | Breast cancer | 0.000244 |
| hsa05165 | Human papillomavirus infection | 0.000284 |
| hsa04211 | Longevity regulating pathway | 0.000723 |
| hsa01521 | EGFR tyrosine kinase inhibitor resistance | 0.000804 |
| hsa05211 | Renal cell carcinoma | 0.000872 |
| hsa05230 | Central carbon metabolism in cancer | 0.000986 |
| hsa05167 | Kaposi sarcoma-associated herpesvirus infection | 0.001215 |
| hsa05218 | Melanoma | 0.001253 |
| hsa04213 | Longevity regulating pathway - multiple species | 0.001353 |
| hsa04068 | FoxO signaling pathway | 0.001375 |
| hsa05214 | Glioma | 0.001763 |
| hsa05212 | Pancreatic cancer | 0.001966 |
| hsa05226 | Gastric cancer | 0.00209 |
| hsa04140 | Autophagy - animal | 0.002201 |
| hsa04910 | Insulin signaling pathway | 0.002201 |
| hsa05216 | Thyroid cancer | 0.002788 |
| hsa04921 | Oxytocin signaling pathway | 0.002971 |
| hsa05213 | Endometrial cancer | 0.003062 |
| hsa04550 | Signaling pathways regulating pluripotency of stem cells | 0.003406 |
